# Synthesis, Structure and Biological Activity of 2-Methyl-5-nitro-6-phenylnicotinohydrazide-Based Hydrazones

**DOI:** 10.3390/molecules30010169

**Published:** 2025-01-04

**Authors:** Oralgazy A. Nurkenov, Anel Z. Mendibayeva, Serik D. Fazylov, Tulegen M. Seilkhanov, Saule K. Kabieva, Ardak K. Syzdykov, Ilya I. Kulakov, Aleksandr V. Iashnikov, Alexey S. Vasilchenko, Larisa E. Alkhimova, Ivan V. Kulakov

**Affiliations:** 1 Institute of Organic Synthesis and Coal Chemistry of the Republic of Kazakhstan, 1 Alikhanov St., Karaganda 100008, Kazakhstan; nurkenov_oral@mail.ru (O.A.N.); iosu8990@mail.ru (S.D.F.); ardak.syzdykov.96@inbox.ru (A.K.S.); 2 Department of Chemical Technology and Ecology, Karaganda Industrial University, 30 Republic Ave., Temirtau 101400, Kazakhstan; kabieva.s@mail.ru; 3 Laboratory of Engineering Profile of NMR Spectroscopy, Sh. Ualikhanov Kokshetau University, 76 Abay St., Kokshetau 020000, Kazakhstan; 4 School of Natural Sciences, University of Tyumen, 15a Perekopskaya St., Tyumen 625003, Russia; i.i.kulakov@utmn.ru (I.I.K.); l.e.alkhimova@utmn.ru (L.E.A.); 5 Laboratory of Antimicrobial Resistance, Institute of Environmental and Agricultural Biology (X-Bio), University of Tyumen, 23 Lenina St., Tyumen 625003, Russia; a.v.yashnikov@utmn.ru (A.V.I.); a.s.vasilchenko@utmn.ru (A.S.V.)

**Keywords:** nicotinic acid, hydrazides, hydrazones, NMR spectroscopy, DFT calculations, conformational analysis, antibacterial activity

## Abstract

The synthetic availability and wide range of biological activity of hydrazides and hydrazones make them attractive subjects for investigation. In this study, we focused on synthesis of 2-methyl-5-nitro-6-phenylnicotinohydrazide-based hydrazones derived from the corresponding substituted aldehydes. The structure of the obtained compounds was studied using NMR spectroscopy and DFT calculations. After repeated recrystallization, all the synthesized compounds remained as mixtures of isomers. As a result of a detailed analysis, we found that the duplication and bifurcation of signals in the ^1^H NMR spectra for some atoms is a consequence of the existence of four isomers, namely *Z*-I, *Z*-II, *E*-I and *E*-II. Duplicate proton signals with a chemical shift difference of 0.1–0.2 ppm and in a ratio of about 2:1 were noticed in the experimental data. By modeling the structures of individual configurations and conformations, Gibbs free energy values were obtained, which allowed us to estimate the approximate content of rotamers for the *E*-isomer equal to 3:2, which coincided with experimental data. We also tested the antibacterial and antifungal activity of the synthesized compounds.

## 1. Introduction

**Scheme 1:**
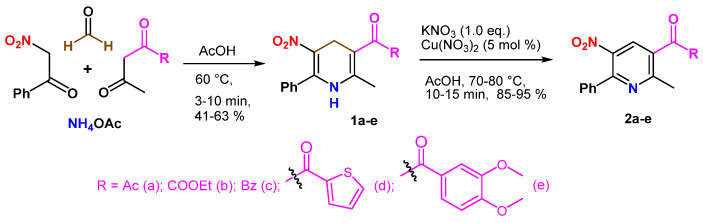
Synthesis of unsymmetrical 5-nitropyridines **2a**–**e**.

Despite the large number of publications on the synthesis, properties and structure of various derivatives of hydrazides and hydrazones, further study and improvement remain promising [1,2,3,4,5]. For instance, hydrazones based on well-known isonicotinic acid hydrazide are used as anti-tuberculosis drugs and antidepressants [6,7,8,9]. At the same time, highly effective biologically active compounds have been found among nicotinic acid derivatives, including antimicrobial, anti-inflammatory and anticonvulsant agents [10,11,12]. This may be due to the high prevalence of nicotinic acid cores in the molecules of many vital natural products, such as alkaloids, vitamins, coenzymes, pigments, etc. [6,7,8,9,10]. In this regard, the further search for pharmacologically active compounds among the hydrazides of pyridine carboxylic acids and compounds similar to them in structure is relevant. For inspiration, we turned to our previous work [13], where we presented an original multicomponent method for the synthesis of asymmetric 5-nitropyridines containing an acetyl or ester group in the C-3 position (Scheme 1).

**Scheme 2:**
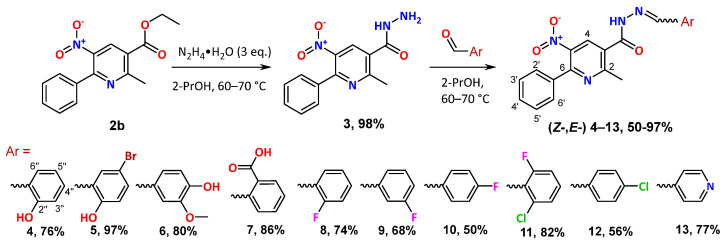
Synthetic route to the **3**-based hydrazones **4**–**13** obtained from corresponding aromatic aldehydes.

One of the compounds obtained during the synthesis, namely 1-(2-methyl-5-nitro-6-phenylpyridin-3-yl)ethan-1-one, was further used by us to obtain new structural analogues of natural integrastatins A, B [14]. In this paper, it became interesting for us to study new compounds based on ethyl ester of 2-methyl-5-nitro-6-phenylnicotinic acid **2**. The corresponding synthesis can be easily performed through conversion of **2b** to hydrazide **3** and its further condensation with corresponding aldehydes as a source of new pharmacophore groups (Scheme 2).

## 2. Results and Discussion

### 2.1. Study of NMR Spectra

All compounds were synthesized in good yields as white crystals (Scheme 1). At the same time, after repeated recrystallization processes, duplicate signals remained in the ^1^H NMR spectra of **4**–**13,** with a difference in chemical shifts of about 0.1–0.2 ppm. Moreover, the peak integration showed that the ratio of the areas of duplicate peaks remained approximately constant for all hydrazones and was equal to 2:1 (Figures S2–S11 in Supplementary Materials). The very first and obvious assumption to explain the observed was the presence of a mixture of *Z*- and *E*-isomers. Additionally, *E*-configuration prevails because this arrangement of atoms made it possible to achieve a more stable geometry of molecules (see the discussion of the DFT calculation results). This was also observed in the signals of the ylidene proton (=CH) in the ^1^H NMR spectra, which were shifted to a downfield due to the influence of the oxygen atom of the carbonyl group in the case of the *E*-isomer (Table 1).

Another marker of a mixture of two isomers was the signal of the H-4 proton from the substituted pyridine core. Thus, in the spectrum of the initial hydrazide, the H-4 proton was registered by a singlet in the region of 8.35 ppm (Table 1). In all the obtained hydrazones, the H-4 proton of the major *E*-isomer was registered by a singlet in the region of 8.53–8.64 ppm, while for the minor *Z*-isomer, the signal of the H-4 proton was slightly shifted to the region of 8.37–8.53 ppm (Table 1). The change of substituents had little effect on the position of H-4 proton singlet; the largest signal displacement was observed in the case of compound **13** (Table 1). As for the =CH proton, the chemical shifts were in a range from 8.17 to 9.07 ppm for the *E*-isomer and from 7.99 to 8.87 ppm for the *Z*-isomer (Table 1). However, **9**, **10**, **12** and **13**, which had substituents at positions 3 and 4 relative to the imine fragment, had the same chemical shift values for both the *E*- and *Z*-isomers, while **4**, **8** and **11** had minimal differences in the values of the chemical shifts, which can be explained by the presence of substituents at position C-2″ relative to the imine substituent (Table 1). Compound **6** had the lowest chemical shift values, which was the result of the presence of two strong donor substituents at positions C-3″ and C-4″ relative to the imine fragment (Table 1).

**Figure 1:**
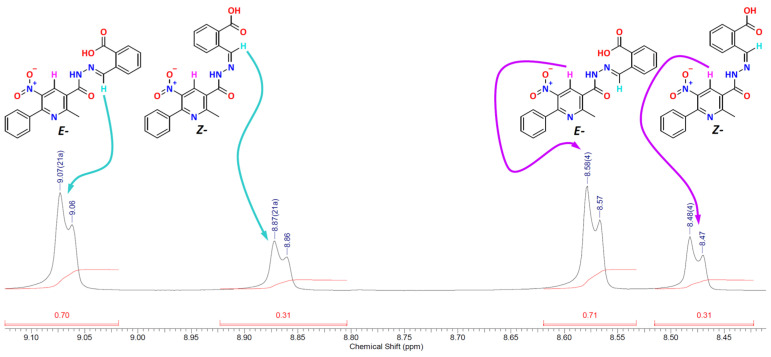
Fragment of ^1^H NMR spectrum of **7**.

A significant shift in the downfield of the =CH proton signal was observed for compound **6** (Figure 1). The chemical shifts for the *E*- and *Z*-isomers were 9.07 and 8.87 ppm, respectively (Figure 1, Table 1), which was 0.7 ppm more in comparison with **9**, **10**, **12** and **13** (Table 1). The COOH group at position C-2″ relative to the imine substituent was an acceptor substituent and additionally provided opportunities for the formation of an intramolecular hydrogen bond with the C=N nitrogen atom in the same way as in the case of **4** and **5** (Table 1).

**Figure 2:**
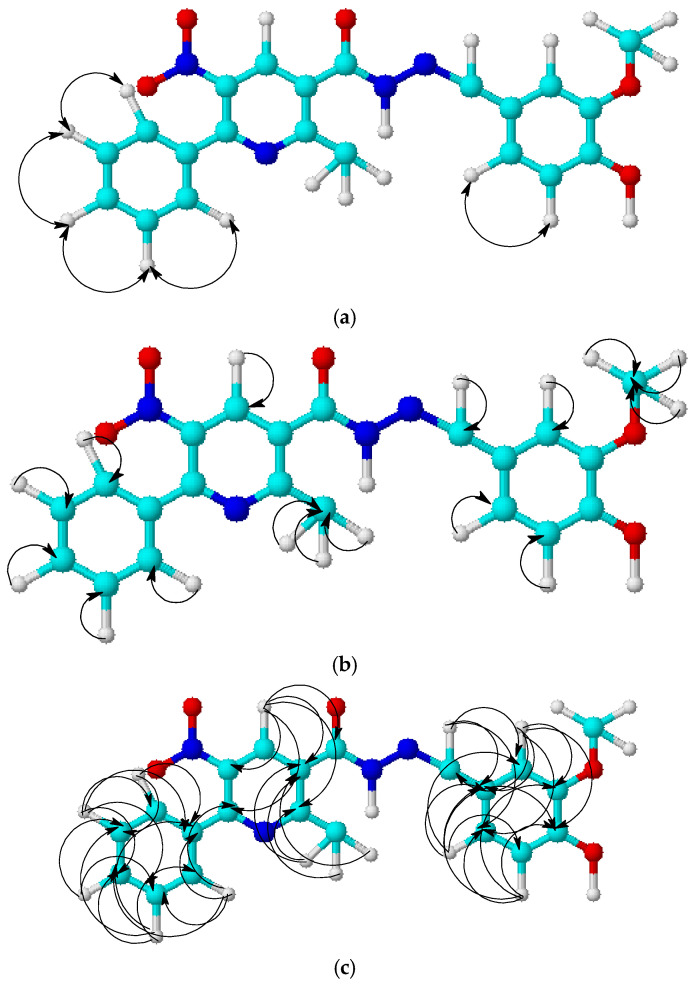
Correlation diagram in the COSY (**a**), HMQC (**b**) and HMBC (**c**) spectra of compound **6**.

For a more complete analysis of the structure of the obtained hydrazones, we analyzed the spectra of two-dimensional NMR spectroscopy COSY (^1^H–^1^H), HMQC (^1^H–^13^C) and HMBC (^1^H–^13^C) using the structure of compound 6 as an example, which allowed us to establish spin-spin interactions of homo- and heteronuclear nature. Some of the observed correlations in the molecule are shown in Figure 2.

In the ^1^H–^1^H COSY spectra of compound **6**, spin-spin correlations were observed through three bonds of protons of neighboring methine–methine protons of aromatic groups: H-5″–H-6″ (6.85, 7.10), H-2′,6′–H-3′,5′ (7.50, 7.50), H-3′,5′–H-4′ (7.50, 7.50) ppm.

Heteronuclear interactions of protons with carbon atoms through one bond were established by ^1^H–^13^C HMQC spectroscopy for the following pairs present in the compound: 2-CH_3_–C-2 (2.68, 23.2), OCH_3_–OCH_3_ (3.81, 55.8), H-5″–C-5″ (6.85, 115.7), H-6″–C-6″ (7.10, 123.0), H-2′,6′–C-2′,6′ (7.50, 128.3), H-3′,5′–C-3′,5′ (7.50, 129.0), N=CH–HC=N (8.17, 149.7), H-2″–C-2″ (7.38, 109.3), H-4–C-4 (8.53, 132.5) ppm.

Heteronuclear interactions of protons with carbon atoms through two or more bonds were established using ^1^H–^13^C HMBC spectroscopy for the following pairs present in the compound: 2-CH_3_–C-3 (2.68, 129.5), 2-CH_3_–C-2 (2.68, 160.3); OCH_3_–C-3″ (3.81, 148.4); H-5″–C-1″ (6.85, 125.5), H-5″–C-3″ (6.85, 148.4); H-6″–C-2″ (7.00, 109.70), H-6″–C=N (7.00, 149.7); H2″–C6″ (7.26, 122.84), H-2″–C=N (7.38, 149.7); N=CH–C-2″ (8.17, 109.3), N=CH–C6″ (8.17, 123.0), N=CH–C-1″ (8.17, 125.5); H-4–C-5 (8.53, 143.6), H-4–C-6 (8.53, 151.9), H-4–C-2 (8.53, 167.3); NH–C=N (11.98, 149.7, NH–C=O (11.98, 161.9) ppm. (See Figures S4.2–S4.4 in Supplementary Materials).

### 2.2. Isomer Modeling

Upon detailed analysis of ^1^H NMR spectra, it was noticed that most of the signals were bifurcated (Figure 1). Our assumption about this phenomenon was the existence of two rotamers, namely I and II, the structures of which were stabilized due to the difficulty of rotation of the substituted pyridine core. The “I” and “II” notation indicates a co-directional arrangement of a hydrogen atom or methyl group with an oxygen atom in a carbonyl group. To test our hypothesis, we performed quantum chemical modeling of four structures, namely *Z*-I, *Z*-II, *E*-I and *E*-II (Figure S12 in Supplementary Materials). The geometries of all structures were energetically minimized; the results are presented in Table 2.

**Figure 3:**
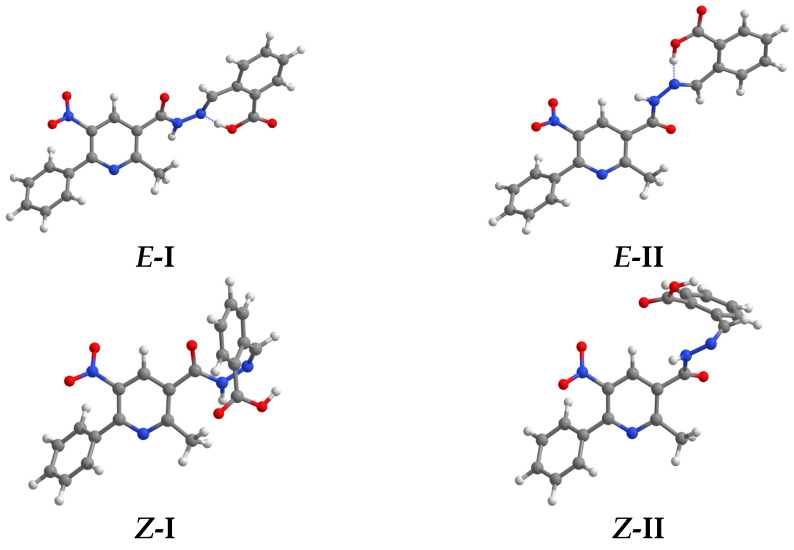
Three-dimensional geometries of suggested configurations and conformations for **7**, obtained using B3LYP-D3(BJ)/def2-TZVPP level of theory in DMSO with the help of the CPCM continuum solvation model.

According to the calculation results, *E*-II isomer is the most energetically stable among all possible configurations and conformations (Table 2). Moreover, II rotamer is more available than the I rotamer with energy differences of 0.3–0.4 kcal/mol for the *E*-isomer and 0.4–1.5 kcal/mol for the *Z*-isomer (Table 2). Compounds **4** and **5** demonstrate the greatest decrease in energy among all other substances, which can be explained by the formation of a pseudo-aromatic ring due to intramolecular hydrogen bonding (Figures S13 and S14 in Supplementary Materials). A similar explanation applies to compound **7**, the COOH group that is capable of forming a seven-membered ring stabilized by a hydrogen bond (Figure 3). When the configuration is changed, this hydrogen bond is destroyed due to the orientation of the corresponding aromatic fragment perpendicular to the plane of the substituted pyridine core (Figure 3). However, **4**, **5** and **7** are still sensitive to conformational transitions, as evidenced by the high absolute values of Gibbs free energy (Table 2).

Since rotation is an equilibrium process in a solvent medium, we can approximately calculate the rotational equilibrium constant and determine the content of each of the two forms (Table S2 in Supplementary Materials). For *E*-isomers, a 3:2 ratio of forms was calculated, which is applicable to all hydrazones with small deviations (Table S2 in Supplementary Materials). It is noteworthy that the ^1^H NMR spectrum of compounds **7**, **11**, **12** also shows a split of signals in the ratio 2:1 (compnd. **7**) (Figure 1) or 1:1 (compnd. **11**, **12**). A less realistic picture is observed for the content of *Z*-isomer rotamers. The ratio of II and I isomers is approximately 7:3 for **6**, **8**–**10**, **12** and **13** (Table S2 in Supplementary Materials). The content of rotamer II for other compounds increases in series **11** < **5** < **4** < **7**, where, for the last compound, it reaches 96% (Table S2 in Supplementary Materials).

We also performed calculations of chemical shifts for all configurations and conformations to verify our early reasoning about NMR spectra. Full data are presented in Tables S3–S12 in Supplementary Materials. The calculated chemical shifts for the H-4 and =CH protons do not exactly match the actual values obtained; however, they allow us to trace some patterns (Table 3). It is obvious that the =CH proton of the *E*-isomer is influenced by an oxygen atom of the carbonyl group in the likeness of a classical intramolecular hydrogen bond, which will lead to a peak shift towards a downfield. It is noticeable that the signals of the =CH proton of the *E*-isomer have greater values than for the *Z*-isomer (Table 1 and Table 3). During the analysis of the chemical shifts of the rotamers themselves, no clear dependencies were revealed. Only compounds **4** and **13** have differences of no more than 0.1 ppm for all isomers, whereas for other substances, a similar dependence is characteristic only for the =CH proton, and for the H-4 proton, the changes can be in the range from 0.2 to 0.3 ppm (Table 3). It is noteworthy that significant changes in the position of chemical shifts for two rotamers are noticeable mostly for the 4-H proton of *E*-isomer (Table 3).

### 2.3. Antibacterial and Antifungal Properties

Despite the mixture of isomers, general antibacterial and antifungal properties of **3**–**13** were further studied. All data are collected in Table 4 and Table 5. In almost all cases, the concentration of 5 mg/mL was insufficient to suppress bacterial growth. However, hydrazide **2** showed activity against *B. cereus* and *P. carotovorum* (Table 4). Compound **5** also demonstrated the same inhibitory properties and additionally showed activity against *S. aureus* 209P (Table 4). The growth inhibition of *B. cereus* and quorum quenching of *C. substugae*, which is only one case of a positive result among all tested substances, was caused by **6** (Table 4). According to the overall results of the study, hydrazone **7** can be called the most effective chemical agent, since it suppresses the growth of *S. aureus*, *B. cereus*, *P. carotovorum* and *C. substugaev* (Table 4). In the case of yeast and mold fungi, **3**–**13** did not show growth inhibition in all tests (Table 5).

## 3. Materials and Methods

### 3.1. Instrumentation and Synthetic Procedures

The ^1^H and ^13^C NMR spectra were acquired on a Jeol JNM-ECA 400 spectrometer (Tokyo, Japan) (400 and 100 MHz, respectively) and Magritek spinsolve 80 carbon ultra (Aachen, Germany) (81 and 20 MHz, respectively) in DMSO-*d*_6_, using TMS or residual solvent signals as internal standard. FTIR spectra were obtained with an Agilent Cary 630 spectrophotometer (Agilent Technologies, California, USA) in a thin sample layer on a crystal attachment. The elemental analysis (C,H,N) was performed on the EuroVector Elemental Analyser device (Pavia, Italy). Melting points were determined using the SMP 10 device (Analog Devices, Wilmington, MA, USA). TLC analysis was performed on Silufol UV-254 plates (Merck, Darmstadt, Germany) manifested with iodine vapor.

2-Methyl-5-nitro-6-phenylnicotinohydrazide (**3**). To a solution of 2.86 g (10 mmol) ethyl ester of 2-methyl-5-nitro-6-phenylnicotinic acid (**1**) in 50 mL of 2-PrOH, 5 mL of 80% hydrazine hydrate is added and heated with intensive stirring at a temperature of 60–70 °C for about an hour. Upon cooling, white lamellar crystals fall out of the flask, which are filtered, washed with cold 2-PrOH and dried. White crystals (2.67 g, 98%), mp 222–224 °C. IR spectrum, ν, cm^−1^: 664; 694; 760; 926; 980; 1180; 1319; 1346, 1516 (–NO_2_); 1366; 1443; 1555; 1597; 1647(C=O); 1744; 2855; 2924; 3052; 3187; 3275 (N–H). ^1^H NMR (80 MHz, DMSO-*d*_6_) *δ* 9.81 (br s, 1H, NH), 8.35 (s, 1H, H-4), 7.51 (s, 5H, Ph), 4.61 (br s, 2H, NH_2_), 2.66 (s, 3H, CH_3_); ^13^C NMR (20 MHz, DMSO-*d*_6_) *δ* 164.7 (C=O), 159.7 (C-2), 151.1 (C-6), 143.3 (C-5), 135.9 (C-3), 132.0 (C-4), 129.7 (C-4′), 129.6 (C-1′), 128.6 (C-3′, C-5′), 128.0 (C-2′, C-6′), 22.9 (CH_3_). Anal. calcd for C_13_H_12_N_4_O_3_: C, 57.35; H, 4.44; N, 20.58; found: C, 57.02; H, 4.72; N, 20.75.

General synthetic procedure for compounds **4**–**13**. To a mixture of 0.5 g (1.8 mmol) of **2** in 10 mL of EtOH, 1.8 mmol of the corresponding aromatic aldehyde is added with stirring. The reaction mixture is stirred at a temperature of 60–70 °C for 6 h, then cooled to room temperature. The raw product is filtered, washed with cold alcohol and dried. The precipitate is recrystallized several times from 2-PrOH.

(*E*-,*Z*-)-*N*′-(2-hydroxybenzylidene)-2-methyl-5-nitro-6-phenylnicotinohydrazide (3:1) (**4**). White crystals (0.54 g, 76%), mp 272–273 °C. IR spectrum, ν, cm^−1^: 694; 760; 856; 964; 1034; 1150; 1204; 1273; 1300; 1365, 1508 (–NO_2_); 1439; 1551; 1636 (C=N); 1659 (C=O); 2851; 2920; 3052; 3229 (N–H); 3420 (–OH). ^1^H NMR (400 MHz, DMSO-*d*_6_) *δ* 12.28 (br s, 1H, NH), 11.00 (br s, 1H, OH), 8.65 (s, 0.75H, =CH (*E*-)), 8.56 (s, 0.75H, H-4 (*E*-)), 8.52 (s, 0.25H, =CH (*Z*-)), 8.45 (s, 0.25H, H-4 (*Z*-)), 7.45–7.65 (m, 5H, Ph), 7.17–7.40 (m, 2H, H-4″,6″ Ar (*E*- + *Z*-)), 6.90–7.00 (m, 2H, H-3″,5″ Ar (*E*- + *Z*-)), 2.74 (s, 2.2H, CH_3_ (*E*-)), 2.60 (s, 0.8H, CH_3_ (*Z*-)); ^13^C NMR (100 MHz, DMSO-*d*_6_) *δ* 167.1 (C=O), 161.4, 160.3, 157.5 (C-2″ Ar), 151.7 (C-6 (*E*-)), 150.9 (C-6 (*Z*-)), 148.6 (CH=), 143.5 (C-5 (*Z*-)), 143.3 (C-5 (*E*-)), 136.0 (C-3 (*Z*-)), 135.9 (C-3 (*E*-)), 132.5 (C-4 (*E*-)), 132.4 (C-4 (*Z*-)), 131.8 (C-4″ Ar (*E*-)), 131.5 (C-4″ Ar (*Z*-)), 129.9 (C-1′ Ph (*E*-)), 129.7 (C-1′ Ph (*Z*-)), 129.1 (C-4′ Ph), 128.7 (C-3′,5′ Ph), 128.1 (C-2′,6′ Ph), 119.5 (C-5″ Ar), 118.7 (C-1″ Ar), 116.4 (C-3″ Ar), 23.1 (CH_3_ (*E*-)), 22.9 (CH_3_ (*Z*-)). Anal. calcd for C_20_H_16_N_4_O_4_: C, 63.82; H, 4.28; N, 14.89; found: C, 63.31; H, 4.14; N, 14.77.

(*E*-,*Z*-)-*N*′-(5-bromo-2-hydroxybenzylidene)-2-methyl-5-nitro-6-phenylnicotinohydrazide (7:3) (**5**). White crystals (0.85 g, 97%), mp 286–288 °C. IR spectrum, ν, cm^−1^: 628 (C-Br); 694; 760; 834; 910; 976; 1030; 1115; 1154; 1188; 1273; 1343, 1551 (–NO_2_); 1443; 1474; 1508; 1601 (C=N); 1659 (C=O); 2855; 2924; 3036; 3198; 3333 (–OH). ^1^H NMR (400 MHz, DMSO-*d*_6_) *δ* 10.13 (br s, 1H, NH), 9.64 (br s, 1H, OH), 8.58 (s, 0.7H, H-4 (*E*-)), 8.48 (s, 0.3H, H-4 (*Z*-)), 8.48 (s, 0.7H, =CH (*E*-)), 8.32 (s, 0.3H, =CH (*Z*-)), 7.81 (s, 0.7H, H-6″ Ar (*E*-)), 7.48–7.55 (m, 5H Ph, 0.3H, H-6″ Ar (*Z*-)), 7.42 (d, 0.7H, *J* = 7.8 Hz, H-4″ Ar (*E*-)), 7.35 (d, 0.3H, *J* = 8.0 Hz, H-4″ Ar (*Z*-)), 6.89 (d, 0.7H, *J* = 8.7 Hz, H-3″ Ar (*E*-)), 6.80 (d, 0.3H, *J* = 8.9 Hz, H-3″ Ar (*Z*-)), 2.70 (s, 2H, CH_3_ (*E*-)), 2.57 (s, 1H, CH_3_ (*Z*-)); ^13^C NMR (100 MHz, DMSO-*d*_6_) *δ* 167.9 (C=O), 162.0, 160.6, 152.1, 146.4 (*Z*-), 143.5 (*Z*-), 139.1 (*Z*-), 136.1 (*E*-), 134.4 (*Z*-), 132.8 (*E*-), 130.3 (*Z*-), 129.0 (2C), 128.4 (2C), 128.3, 125.3, 121.5, 119.0 (*E*-), 110.9 (*Z*-), 23.4 (CH_3_). Anal. calcd for C_20_H_15_BrN_4_O_4_: C, 52.76; H, 3.32; N, 12.31; found: C, 53.04; H, 3.14; N, 12.24.

(*E*-,*Z*-)-*N*′-(4-hydroxy-3-methoxybenzylidene)-2-methyl-5-nitro-6-phenylnicotinohydrazide (7:3) (**6**). White crystals (0.62 g, 80%), mp 260–262 °C. IR spectrum, ν, cm^−1^: 694; 756; 899; 868; 1026; 1165 (–OCH_3_); 1208; 1277; 1346, 1512 (–NO_2_); 1555; 1601 (C=N); 1655 (C=O); 2361; 2855 (–OCH_3_); 2928; 3059; 3210 (N–H); 3433 (–OH). ^1^H NMR (400 MHz, DMSO-*d*_6_) *δ* 11.98 (br s, 1H, NH), 9.81 (br s, 1H, OH), 8.53 (s, 0.7H, H-4 (*E*-)), 8.48 (s, 0.3H, H-4 (*Z*-)), 8.17 (s, 0.7H, =CH (*E*-)), 7.99 (s, 0.3H, =CH (*Z*-)), 7.38 (s, 1H, H-2′′ Ar), 7.42–7.59 (m, 5H, Ph), 6.93–7.17 (m, 1H, H-6′′, Ar (*E*- + *Z*-)), 6.85 (br. s, 0.7H, H-5″ (*E*-) Ar), 6.75 (br.s, 0.3H, H-5″ (*Z*-) Ar), 3.81 (s, 3H, OCH_3_), 2.68 (s, 2H, CH_3_ (*E*-)), 2.58 (s, 1H, CH_3_ (*Z*-)); ^13^C NMR (100 MHz, DMSO-*d*_6_) *δ* 167.3 (C=O (*E*-)), 167.2 (C=O (*Z*-)), 161.9, 160.3, 151.9, 149.7 (*E*-), 149.6 (*Z*-), 148.4 (*E*-), 148.2 (*Z*-), 143.6 (*E*-), 143.5 (*Z*-), 136.3 (*Z*-), 136.2 (*E*-), 133.1 (*Z*-), 132.5 (*E*-), 130.2 (*E*-), 130.1 (*Z*-), 129.5, 129.0 (2C), 128.3 (2C), 125.5, 123.0, 115.7, 109.3 (*E*-), 109.0 (*Z*-), 55.8 (OCH_3_ (*E*-)), 55.3 (OCH_3_ (*Z*-)), 23.2 (CH_3_). Anal. calcd for C_21_H_18_N_4_O_5_: C, 62.06; H, 4.46; N, 13.79; found: C, 62.49; H, 4.23; N, 13.96.

(*E*-,*Z*-))-2-((2-(2-methyl-5-nitro-6-phenylnicotinoyl)hydrazono)methyl)benzoic acid (7:3) (**7**). White crystals (0.66 g, 86%), mp 235–237 °C. IR spectrum, ν, cm^−1^: 691; 752; 833; 922; 945; 1034; 1076; 1154; 1281; 1350, 1520 (–NO_2_); 1393; 1451; 1551; 1601 (C=N); 1678 (C=O); 2508; 2635; 2928; 2962; 3052; 3437 (–OH). ^1^H NMR (400 MHz, DMSO-*d*_6_) *δ* 12.36 (br s, 0.35H, NH (*Z*-)), 12.32 (br s, 0.65H, NH (*E*-)), 9.07 (s, 0.7H, =CH (*E*-)), 8.87 (s, 0.3H, =CH (*Z*-)), 8.58 (s, 0.7H, H-4 (*E*-)), 8.48 (s, 0.3H, H-4 (*Z*-)), 8.10 (d, 0.7H, *J* = 6.8 Hz, H-3′′ Ar (*E*-)), 7.84 (d, 0.3H, *J* = 6.7 Hz, H-3′′ Ar (*Z*-)), 7.91 (d, 0.7H, *J* = 7.4 Hz, H-6′′ Ar (*E*-)), 7.66 (d, 0.3H, *J* = 6.9 Hz, H-6′′ Ar (*Z*-)), 7.64 (t, 0.7H, *J* = 5.9 Hz, H-5′ Ar (*E*-)), 7.46–7.56 (m, 5H, Ph, 1H, H-4′′ Ar (*E*- + *Z*-), 0.3H, H-5′′ Ar (*Z*-)), 2.70 (s, 2H, CH_3_ (*E*-)), 2.59 (s, 1H, CH_3_ (*Z*-)); ^13^C NMR (100 MHz, DMSO-*d*_6_) *δ* 168.4 (*E*-), 168.3 (*Z*-), 162.4, 160.5, 152.0 (*E*-), 151.3 (*Z*-), 148.2, 143.7 (*Z*-), 143.6 (*E*-), 136.2 (*Z*-), 136.1 (*E*-), 134.4 (*E*-), 134.2 (*Z*-), 132.9 (*Z*-), 132.8 (*E*-), 132.5 (*E*-), 132.4 (*Z*-), 131.1 (*E*-), 131.0 (*Z*-), 130.8 (*E*-), 130.7 (*Z*-), 130.5, 130.3 (*E*-), 130.2 (*Z*-), 129.2, 129.1 (1C (*E*-)), 129.0 (3C (*Z*-)), 128.43 (1C (*Z*-)), 128.37 (3C (*E*-)), 127.1 (*E*-), 126.6 (*Z*-, 23.3 (CH_3_ (*E*-)), 23.2 (CH_3_ (*Z*-)). Anal. calcd for C_21_H_16_N_4_O_5_: C, 62.37; H, 3.99; N, 13.86; found: C, 62.28; H, 4.15; N, 13.95.

(*E*-,*Z*-)-*N*′-(2-fluorobenzylidene)-2-methyl-5-nitro-6-phenylnicotinohydrazide (3:2) (**8**). White crystals (0.52 g, 74%), mp 283–285 °C. IR spectrum, ν, cm^−1^: 698; 760; 772; 837; 887; 937; 961; 1061; 1096 (C–F); 1300; 1343, 1520 (–NO_2_); 1370; 1451; 1481; 1551; 1598 (C=N); 1651 (C=O); 1709; 2855; 2924; 2924; 2967; 3168; 3275 (N–H). ^1^H NMR (400 MHz, DMSO-*d*_6_) *δ* 12.23 (br s, 1H, NH), 8.65 (br s, 0.6H, =CH (*E*-)), 8.53 (br s, 1 H, H-4 (*E*- + *Z*-)), 8.34 (br s, 0.4H, =CH (*Z*-)), 7.19–7.96 (m, 9H, 5H, Ph, H-2′′,3′′,4′′,5′′ Ar (*E*- + *Z*-)), 2.72 (s, 2H, CH_3_ (*E*-)), 2.59 (s, 1H, CH_3_ (*Z*-)); ^13^C NMR (100 MHz, DMSO-*d*_6_) *δ* 167.4 (C=O), 160.9 (d, 1*J*_13*C−F*_ = 145 Hz, C-2″), 159.7, 158.5, 151.7, 143.4, 141.5, 138.4, 135.8, 132.75 (d, 3*J*_13*C−F*_ = 8.6 Hz, C-4″ Ar), 132.5 (*E*- +*Z*-), 129.95 (d, 3*J*_13*C−F*_ = 9.6 Hz, C-6″ Ar), 128.9, 128.7 (C-3′,5′ Ph), 128.1 (C-2′,6′ Ph), 125.1 (*Z*- + *E*-), 121.4 (d, 2*J*_13*C−F*_ = 26 Hz, C-1″), 116.14 (d, 2*J*_13*C−F*_ = 22 Hz, C-3′′), 23.1 (CH_3_). Anal. calcd for C_20_H_15_FN_4_O_3_: C, 63.49; H, 4.00; N, 14.81; found: C, 63.72; H, 4.27; N, 14.65.

(*E*-,*Z*-)-*N*′-(3-fluorobenzylidene)-2-methyl-5-nitro-6-phenylnicotinohydrazide (6.5:3.5) (**9**). White crystals (0.48 g, 68%), mp 257–259 °C. IR spectrum, ν, cm^−1^: 691; 760; 837; 868; 980; 1134 (C–F); 1269; 1300; 1343, 1555 (–NO_2_); 1443; 1516; 1612 (C=N); 1663 (C=O); 1740; 2338; 2361; 2859; 2924; 3063; 3198; 3275 (N–H). ^1^H NMR (400 MHz, DMSO-*d*_6_) *δ* 12.24 (br s, 1H, NH), 8.58 (s, 0.65H, H-4 (*E*-)), 8.49 (s, 0.35H, H-4 (*Z*-)), 8.31 (s, 0.65H, =CH (*E*-)), 8.13 (s, 0.35H, =CH (*Z*-)), 7.18–7.57 (m, 9H, 5H Ph, H-2′,3′,4′,6′ Ar (*E*- + *Z*-)), 2.71 (s, 2H, CH_3_ (*E*-)), 2.58 (s, 1H, CH_3_ (*Z*-)); ^13^C NMR (100 MHz, DMSO-*d*_6_) *δ* 167.7 (C=O), 161.4 (C-2 (*E*-)), 161.3 (C-2 (*Z*-)), 161.3 (d, 1*J*_13*C−F*_ = 187 Hz, C-4″), 158.8, 152.0 (*E*-), 151.3 (*Z*-), 147.8, 144.3 (*E*-), 143.5 (*Z*-), 136.58 (d, 3*J*_13*C−F*_ = 8 Hz, C-1′′ Ar), 136.1 (*Z*-), 136.0 (*E*-), 133.0 (C-4 (*Z*-)), 132.6 (C-4 (*E*-)), 131.27 (d, 3*J*_13*C−F*_ = 8 Hz, C-5″ Ar), 130.2 (*E*-), 130.1 (*Z*-), 129.2, 129.0 (C-3′,5′ Ph), 128.3 (C-2′,6′ Ph), 124.0 (*E*-), 123.4 (*Z*-), 117.2 (d, 2*J*_13*C−F*_ = 26 Hz, C-4″), 113.5 (d, 2*J*_13*C−F*_ = 22 Hz, C-2″), 23.2 (CH_3_ (*E*-)), 23.1 (CH_3_ (*Z*-)). Anal. calcd for C_20_H_15_FN_4_O_3_: C, 63.49; H, 4.00; N, 14.81; found: C, 63.82; H, 4.15; N, 15.05.

(*E*-,*Z*-)-*N*′-(4-fluorobenzylidene)-2-methyl-5-nitro-6-phenylnicotinohydrazide (6.5:3.5) (**10**). White crystals (0.35 g, 50%), mp 268–270 °C. IR spectrum, ν, cm^−1^: 610; 698; 760; 845; 880; 968; 1045; 1134 (C–F); 1157; 1235; 1292; 1346, 1512 (–NO_2_); 1447; 1555; 1601 (C=N); 1667 (C=O); 2365; 2855; 2928; 2974; 3055; 3383 (N–H). ^1^H NMR (400 MHz, DMSO-*d*_6_) *δ* 12.16 (br s, 1H, NH), 8.57 (s, 0.65H, H-4 (*E*-)), 8.47 (s, 0.35H, H-4 (*Z*-)), 8.29 (s, 0.65H, =CH (*E*-)), 8.13 (s, 0.35H, =CH (*Z*-)), 7.81 (br s, 1.3H, H-2′′,6′′ Ar (*E*-)), 7.58 (br s, 0.7H, H-2′′,6′′ Ar (*Z*-)), 7.46–7.56 (m, 5H, Ph), 7.29 (br s, 1.3H, H-3′′,5′′ Ar (*E*-)), 7.19 (br s, 0.7H, H-3′′,5′′ Ar (*Z*-)), 2.70 (s, 2H, CH_3_ (*E*-)), 2.58 (s, 1H, CH_3_ (*Z*-)); ^13^C NMR (100 MHz, DMSO-*d*_6_) *δ* 162.5 (C=O), 161.4 (d, 1*J*_13*C−F*_ = 185 Hz, C-4′′), 158.9, 152.1, 148.3, 144.9 (*Z*-), 143.6 (*E*-), 136.3 (*Z*-), 136.2 (*E*-), 132.9 (*Z*-), 132.7 (*E*-), 130.7 (*E*-), 130.6 (*Z*-), 130.3 (*E*-), 130.2 (*Z*-), 130.0 (d, 3*J*_13*C−F*_ = 8 Hz, C-2′′,6′′ Ar), 129.4, 129.1 (C-3′,5′ Ph), 128.4 (C-2′,6′ Ph), 116.4 (d, 2*J*_13*C−F*_ = 24 Hz, C-3′′,5′′), 23.3 (CH_3_). Anal. calcd for C_20_H_15_FN_4_O_3_: C, 63.49; H, 4.00; N, 14.81; found: C, 63.32; H, 4.18; N, 15.12.

(*E*-,*Z*-)-*N*′-(2-chloro-6-fluorobenzylidene)-2-methyl-5-nitro-6-phenylnicotinohydrazide (4.5:5.5) (**11**). White crystals (0.64 g, 82%), mp 230–231 °C. IR spectrum, ν, cm^−1^: 705; 760 (C–Cl); 1090; 1133 (C–F); 1345, 1565 (–NO_2_); 1620 (C=N); 1665 (C=O); 3227 (N–H). ^1^H NMR (400 MHz, DMSO-*d*_6_) *δ* 12.38 (br s, 1H, NH (*E*- + *Z*-)), 8.67 (s, 0.45H, =CH (*E*-)), 8.57 (s, 0.45H, H-4 (*E*-)), 8.47 (s, 0.55H, =CH (*Z*-)), 8.37 (s, 0.55H, H-4 (*Z*-)), 7.15–7.45 (m, 3H, H-3″,4″,5″ Ar (*E*- + *Z*-)), 7.45–7.60 (m, 5H, Ph), 2.73 (s, 1.3H, CH_3_ (*E*-)), 2.60 (s, 1.7H, CH_3_ (*Z*-)); ^13^C NMR (100 MHz, DMSO-*d*_6_) *δ* 167.7 (C=O), 160.1 (d, 1*J*_13*C−F*_ = 315 Hz, C-1″), 160.3 (C-2 (*E*-)), 159.1 (C-2 (*Z*-)), 151.7 (C-6 (*E*-)), 150.9 (C-6 (*Z*-)), 143.3 (CH= (*E*-)), 143.2 (CH= (*Z*-)), 142.4 (C-5 (*E*-)), 142.2 (3*J*_13*C−F*_ = 16.3 Hz, C-5 (*Z*-)), 138.3 (d, C-2″ Ar), 135.9 (d, 3*J*_13*C−F*_ = 17.2 Hz, C-4″ Ar), 134.1 (C-3 (*E*-)), 133.3 (C-3 (*Z*-)), 132.6 (C-4 (*E*-)), 132.5 (C-4 (*Z*-)), 130.0 (C-4′ Ph), 128.7 (C-3′,5′ Ph), 128.0 (C-2′,6′ Ph), 126.3 (C-3″ (*Z*-)), 126.1 (C-3″ (*E*-)), 119.8 (d, 2*J*_13*C−F*_ = 13 Hz, C-1″), 115.66 (d, 2*J*_13*C−F*_ = 22 Hz, C-5″), 23.1 (CH_3_ (*E*-)), 22.8 (CH_3_ (*Z*-)). Anal. calcd for C_20_H_14_ClFN_4_O_3_: C, 58.19; H, 3.42; N, 13.57; found: C, 57.92; H, 3.63 N, 12.97.

(*E*-,*Z*-)-*N*′-(4-chlorobenzylidene)-2-methyl-5-nitro-6-phenylnicotinohydrazide (6.5:3.5) (**12**). White crystals (0.41 g, 56%), mp 270–272 °C. IR spectrum, ν, cm^−1^: 637; 691; 756 (C–Cl); 833; 934; 1092; 1269; 1339, 1555 (–NO_2_); 1400; 1443; 1512; 1597; 1607 (C=N); 1659 (C=O); 2855; 2924; 3059; 3198 (N–H). ^1^H NMR (400 MHz, DMSO-*d*_6_) *δ* 12.31 (br s, 0.3H, NH (*Z*-)), 12.18 (br s, 0.7H, NH (*E*-)), 8.62 (s, 0.65H, H-4 (*E*-)), 8.51 (s, 0.35H, H-4 (*Z*-)), 8.31 (s, 0.65H, =CH (*E*-)), 8.14 (s, 0.35H, =CH (*Z*-)), 7.75–7.85 (m, 1.3H, H-2′′,6′′ Ar (*E*-)), 7.41–7.65 (m, 5H, Ph, 2H, H-3′′,5′′ Ar (*E*- + *Z*-), 0.7H, H-2′′,6′′ Ar (*Z*-)), 2.72 (s, 2H, CH_3_ (*E*-)), 2.58 (s, 1H, CH_3_ (*Z*-)); ^13^C NMR (100 MHz, DMSO-*d*_6_) *δ* 167.4 (C=O), 161.7 (C-2), 151.6 (C-6 (*E*-)), 151.0 (C-6 (*Z*-)), 147.5 (CH=), 144.2 (C-5 (*Z*-)), 143.4 (C-5 (*E*-)), 136.0 (C-4″ Ar (*Z*-)), 135.9 (C-4″ Ar (*E*-)), 135.0 (C-3 (*E*-)), 134.7 (C-3 (*Z*-)), 132.9 (C-1″ Ar (*E*-)), 132.7 (C-1″ Ar (*Z*-)), 132.6 (C-4 (*Z*-)), 132.4 (C-4 (*E*-)), 129.95 (C-1′ Ph (*E*-)), 129.80 (C-1′ Ph (*Z*-)), 129.04 (C-2″,6″ Ar), 128.98 (C-3″, 5″ Ar), 128.7 (C-3′,5′ Ph), 128.5 (C-4′ Ph), 128.1 (C-2′,6′ Ph), 23.3 (CH_3_). Anal. calcd for C_20_H_15_ClN_4_O_3_: C, 60.84; H, 3.83; N, 14.19; found: C, 60.58; H, 3.97; N, 13.98.

(*E*-,*Z*-)-2-methyl-5-nitro-6-phenyl-*N*′-(pyridin-4-ylmethylene)nicotinohydrazide (7:3) (**13**). White crystals (0.52 g, 77%), mp 272–273 °C. IR spectrum, ν, cm^−1^: 694; 937; 976; 1072; 1111; 1142; 1343, 1512 (–NO_2_); 1439; 1555; 1607 (C=N); 1670 (C=O); 1871; 1952; 2855; 2920; 3071; 3214 (N–H). ^1^H NMR (400 MHz, DMSO-*d*_6_) *δ* 12.39 (br s, 1H, NH (*E*- + *Z*-)), 8.67 (br s, 1.2H, H-2″,6″ Py (*E*-)), 8.64 (s, 0.7H, H-4 (*E*-)), 8.56 (br s, 0.8H, H-2″,6″ Py (*Z*-)), 8.53 (s, 0.3H, H-4 (*Z*-)), 8.31 (s, 0.65H, =CH (*E*-)), 8.13 (s, 0.35H, =CH (*Z*-)), 7.70 (br s, 1.2H, H-3″,5″ Py (*E*-)), 7.48–7.65 (m, 5H, Ph), 7.39 (br s, 0.8H, H-3″,5″ Py (*Z*-)), 2.73 (s, 1.9H, CH_3_ (*E*-)), 2.60 (s, 1.1H, CH_3_ (*Z*-)); ^13^C NMR (100 MHz, DMSO-*d*_6_) *δ* 167.7 (C=O), 162.0 (C-2 (*Z*-)), 160.1 (C-2 (*E*-)), 151.7 (C-6 (*E*-)), 151.1 (C-6 (*Z*-)), 150.3 (C-2″,6″ Py (*E*- + *Z*-)), 146.4 (CH=), 143.3 (C-5 (*E*-)), 143.0 (C-5 (*Z*-)), 141.1 (C-4″ Py (*E*-)), 140.8 (C-4″ Py (*Z*-)), 136.0 (C-3 (*Z*-)), 135.8 (C-3 (*E*-)), 132.8 (C-4 (*Z*-)), 132.5 (C-4 (*E*-)), 129.9 (C-1′ Ph (*E*-)), 129.8 (C-1′ Ph (*Z*-)), 128.8 (C-4′ Ph), 128.7 (C-3′,5′ Ph), 128.1 (C-2′,6′ Ph), 121.1 (C-3″,5″ Py (*E*-)), 120.7 (C-3″,5″ Py (*Z*-)), 23.0 (CH_3_). Anal. calcd for C_19_H_15_N_5_O_3_: C, 63.15; H, 4.18; N, 19.38; found: C, 62.90; H, 4.39; N, 19.03.

### 3.2. DFT Calculations

The DFT optimization and NMR-GIAO calculations were performed using Orca 5 quantum chemistry program package [15,16]. All geometries were energetically minimized with the help of B3LYP functional [17,18,19], D3 Becke-Johnson (BJ) [20] dispersion correction, def2-TZVPP [21] basis set and conductor-like polarizable continuum model (CPCM) [22,23] to take into account the solvation effect of DMSO. The corresponding structures were further used for NMR-GIAO calculations with the same level of theory. The structure of tetramethylsilane (TMS) was also optimized to obtain reference NMR shifts. Cartesian coordinates of optimized geometries, thermodynamic data and calculated ^1^H and ^13^C NMR chemical shifts are presented in Figures S12–S22 and Table 2 and Table S1–S22 in Supplementary Materials.

### 3.3. Biological Assay

The antimicrobial abilities of synthesized hydrazones were studied in the experiment with bacterial and fungal microorganisms: *Staphylococcus aureus* 209P and ATCC 29923 (Gram-positive facultative anaerobe); *Staphylococcus epidermidis* (Gram-positive facultative anaerobe); *Enterococcus faecium* 79 OSAU (Gram-positive facultative anaerobe); *Bacillus cereus* IP 5832 (Gram-positive facultative anaerobe); *Escherichia coli* K12 (Gram-negative facultative anaerobe); *Pectobacterium carotovorum* VKM-B-1247 (Gram-negative facultative anaerobe); *Chromobacterium substugae* ATCC 31532 (Gram-negative facultative anaerobe); *Candida albicans* ATCC 10231 (yeast); *Aspergillus niger* INA 007760 (mold). A Luria–Bertani (LB) medium (10 mg/mL of tryptone, 5 mg/mL of yeast extract and 5 mg/mL of sodium chloride) was used for bacteria cultivation, while *C. albicans* was grown on SDA (40 mg/mL of dextrose and 10 mg/mL of Peptone), and *A. niger* was grown on PDA (23 mL of potato extract, 20 mg/mL of glucose and 77 mL of distilled water). The studied hydrazones were dissolved in DMSO (99.5%, Applichem, Darmstadt, Germany). The negative control was a 1% water solution of DMSO. The final concentration of the tested compounds was 5 mg/mL. The hydrazone compounds were evaluated using the agar diffusion assay [24]. Briefly, the culture of the studied bacteria or fungi was diluted 10 times in the LB medium with non-solidified 0.75% agar (*w*/*w*) on sterile Petri dishes. A drop of the tested sample was applied to the surface of an agar plate, and after 24 h of incubation, the results were assessed by the appearance of zones of inhibition. In the case of *C. substugae*, we took into account the colorless zone where purple pigment was not produced by this bacterium (an indicator of QQ).

## 4. Conclusions

We have reported the synthesis, structure and biological activity of new hydrazones derived from 2-methyl-5-nitro-6-phenylnicotinohydrazide and corresponding aldehydes. Despite the repeated recrystallization, duplicate proton signals with a chemical shift difference of 0.1–0.2 ppm and in a ratio of about 2:1 were recorded in the ^1^H NMR spectra. Our main explanation was based on the existence of a mixture of *Z*- and *E*-isomers, which also exist mainly in the form of two rotamers, namely II and I. As a result of modeling each isomer using DFT calculations, we were able to determine that for *E*-isomers, the ratio of the content of rotamers is approximately the same for all compounds and equal to 3:2, which coincides with experimental results. During the study of antibacterial activity, *B. cereus* was most sensitive to the action of chemical agents, namely **3**, **5**, **6** and **7**, while compound **7** demonstrated the widest range of action. All the compounds had no effect on fungal cultures, regardless of their species.

## Figures and Tables

**Table 1:** ^1^H NMR chemical shifts, *δ* (ppm) for substituted pyridine and imine hydrogen atoms, namely H-4 and =CH, respectively.

Compound	*δ* (H-4 (*E*-))	*δ* (H-4 (*Z*-))	*δ* (=CH (*E*-))	*δ* (=CH (*Z*-))
**3**	8.35	–	–	–
**4**	8.56	8.45	8.65	8.52
**5**	8.58	8.48	8.48	8.32
**6**	8.53	8.48	8.17	7.99
**7**	8.58	8.48	9.07	8.87
**8**	8.53	8.53	8.65	8.34
**9**	8.58	8.49	8.31	8.13
**10**	8.57	8.47	8.29	8.13
**11**	8.57	8.37	8.67	8.47
**12**	8.62	8.51	8.31	8.14
**13**	8.64	8.53	8.31	8.13

**Table 2:** Relative Gibbs free energy values, ∆*G* (kcal/mol) for **4**–**13**, obtained using B3LYP-D3(BJ)/def2-TZVPP level of theory in DMSO with the help of the CPCM continuum solvation model.

Compound	*Z*-I ^1^	*Z*-II	*E*-I	*E*-II
**4**	0.0	−1.5	−9.7	−10.0
**5**	0.0	−1.5	−9.9	−10.1
**6**	0.0	−0.5	−6.3	−6.6
**7**	0.0	−1.9	−8.0	−8.3
**8**	0.0	−0.5	−3.5	−3.8
**9**	0.0	−0.4	−4.5	−4.9
**10**	0.0	−0.4	−4.3	−4.7
**11**	0.0	−0.7	−1.8	−2.1
**12**	0.0	−0.5	−4.5	−4.8
**13**	0.0	−0.4	−4.8	−5.2

^1^ The energy of *Z*-I-isomer was selected as a starting point to illustrate the decrease in energy of the system towards a more stable configuration and conformation.

**Table 3:** ^1^H NMR chemical shifts, *δ* (ppm), obtained using GIAO//B3LYP-D3(BJ)/def2-TZVPP level of theory in DMSO with the help of the CPCM continuum solvation model, for substituted pyridine and imine hydrogen atoms, namely H-4 and =CH, respectively.

Compound	*δ* (H-4 (*Z*-I))	*δ* (H-4 (*Z*-II))	*δ* (H-4 (*E*-I))	*δ* (H-4 (*E*-II))	*δ* (=CH (*Z*-I))	*δ* (=CH (*Z*-II))	*δ* (=CH (*E*-I))	*δ* (=CH (*E*-II))
**4**	8.74	8.73	8.67	8.67	8.84	8.86	10.84	10.81
**5**	8.74	8.7	8.78	8.55	8.76	8.73	10.81	10.78
**6**	8.96	8.66	8.83	8.52	8.27	8.34	10.89	10.84
**7**	8.87	8.88	8.82	8.63	8.93	9.02	10.93	10.98
**8**	8.85	8.65	8.78	8.57	8.48	8.93	10.89	10.84
**9**	8.83	8.71	8.83	8.6	8.49	8.56	10.87	10.73
**10**	8.55	8.61	8.78	8.56	8.49	8.51	10.82	10.85
**11**	8.47	8.63	8.73	8.66	8.72	8.69	11.39	11.34
**12**	8.84	8.7	8.77	8.57	8.47	8.63	10.82	10.83
**13**	8.68	8.69	8.72	8.69	8.6	8.65	10.77	10.78

**Table 4:** Antibacterial properties of **3**–**13**.

Bacteria	Parameter ^1^	Compound											Control (1% DMSO)
		3	4	5	6	7	8	9	10	11	12	13	
*Staphylococcus aureus* 209P	GI	−	−	+	−	+	−	−	−	−	−	−	−
*Staphylococcus aureus* ATCC 29923	GI	−	−	−	−	+	−	−	−	−	−	−	−
*Staphylococcus epidermidis*	GI	−	−	−	−	−	−	−	−	−	−	−	−
*Enterococcus. faecium* 79 OSAU	GI	−	−	−	−	−	−	−	−	−	−	−	−
*Bacillus cereus* IP 5832	GI	+	−	+	+	+	−	−	−	−	−	−	−
*Escherichia coli* K12	GI	−	−	−	−	−	−	−	−	−	−	−	−
*Pectobacterium carotovorum* VKM-B-1247	GI	+	−	+	−	+	−	−	−	−	−	−	−
*Chromobacterium substugae* ATCC 31532	GI	−	−	−	−	+	−	−	−	−	−	−	−
	QQ	−	−	−	+	−	−	−	−	−	−	−	−

^1^ “GI”: growth inhibition; “QQ”: quorum quenching; “+”: parameter is positive; “−”: parameter is negative (i.e., t.i., does not affect growth).

**Table 5:** Antifungal properties of **3**–**13**.

Bacteria	Parameter ^1^	Compound											Control (1% DMSO)
		3	4	5	6	7	8	9	10	11	12	13	
*Candida albicans* ATCC 10231	GI	−	−	−	−	−	−	−	−	−	−	−	−
*Aspergillus niger* INA 007760	GI	−	−	−	−	−	−	−	−	−	−	−	−

^1^ “GI”: growth inhibition; “−”: parameter is negative (i.e., t.i., does not affect growth).

## Data Availability

The original contributions presented in the study are included in the article and Supplementary Materials, further inquiries can be directed to the corresponding authors.

## Supplementary Materials

The following supporting information can be downloaded at https://www.mdpi.com/article/10.3390/molecules30010169/s1: Figure S1: ^1^H NMR (400 MHz, DMSO-*d*_6_) and ^13^C NMR (100 MHz, DMSO-*d*_6_) spectra of **3**; Figure S2: ^1^H NMR (400 MHz, DMSO-*d*_6_) and ^13^C NMR (100 MHz, DMSO-*d*_6_) spectra of **4**; Figure S3: ^1^H NMR (400 MHz, DMSO-*d*_6_) and ^13^C NMR (100 MHz, DMSO-*d*_6_) spectra of **5**; Figure S4: ^1^H NMR (400 MHz, DMSO-*d*_6_) and ^13^C NMR (100 MHz, DMSO-*d*_6_) spectra of **6**; Figure S5: ^1^H NMR (400 MHz, DMSO-*d*_6_) and ^13^C NMR (100 MHz, DMSO-*d*_6_) spectra of **7**; Figure S6: ^1^H NMR (400 MHz, DMSO-*d*_6_) and ^13^C NMR (100 MHz, DMSO-*d*_6_) spectra of **8**; Figure S7: ^1^H NMR (400 MHz, DMSO-*d*_6_) and ^13^C NMR (100 MHz, DMSO-*d*_6_) spectra of **9**; Figure S8: ^1^H NMR (400 MHz, DMSO-*d*_6_) and ^13^C NMR (100 MHz, DMSO-*d*_6_) spectra of **10**; Figure S9: ^1^H NMR (400 MHz, DMSO-*d*_6_) and ^13^C NMR (100 MHz, DMSO-*d*_6_) spectra of **11**; Figure S10: ^1^H NMR (400 MHz, DMSO-*d*_6_) and ^13^C NMR (100 MHz, DMSO-*d*_6_) spectra of **12**; Figure S11: ^1^H NMR (400 MHz, DMSO-*d*_6_) and ^13^C NMR (100 MHz, DMSO-*d*_6_) spectra of **13**; Figure S12: 2D structures of possible configurations and conformations for **4**–**13**; Figure S13: Optimized geometry of **4**, obtained using B3LYP-D3(BJ)/def2-TZVPP level of theory in DMSO with the help of the CPCM continuum solvation model; Figure S14: Optimized geometry of **5**, obtained using B3LYP-D3(BJ)/def2-TZVPP level of theory in DMSO with the help of the CPCM continuum solvation model; Figure S15: Optimized geometry of **6**, obtained using B3LYP-D3(BJ)/def2-TZVPP level of theory in DMSO with the help of the CPCM continuum solvation model; Figure S16: Optimized geometry of **7**, obtained using B3LYP-D3(BJ)/def2-TZVPP level of theory in DMSO with the help of the CPCM continuum solvation model; Figure S17: Optimized geometry of **8**, obtained using B3LYP-D3(BJ)/def2-TZVPP level of theory in DMSO with the help of the CPCM continuum solvation model; Figure S18: Optimized geometry of **9**, obtained using B3LYP-D3(BJ)/def2-TZVPP level of theory in DMSO with the help of the CPCM continuum solvation model; Figure S19: Optimized geometry of **10**, obtained using B3LYP-D3(BJ)/def2-TZVPP level of theory in DMSO with the help of the CPCM continuum solvation model; Figure S20: Optimized geometry of **11**, obtained using B3LYP-D3(BJ)/def2-TZVPP level of theory in DMSO with the help of the CPCM continuum solvation model; Figure S21: Optimized geometry of **12**, obtained using B3LYP-D3(BJ)/def2-TZVPP level of theory in DMSO with the help of the CPCM continuum solvation model; Figure S22: Optimized geometry of **13**, obtained using B3LYP-D3(BJ)/def2-TZVPP level of theory in DMSO with the help of the CPCM continuum solvation model; Table S1: Gibbs free energy, ∆*G* (kcal/mol) differences in values for corresponding configurations and conformations of **4**–**13**; Table S2: Conformational constants, *K* for II*→*I-transition and equilibrium content of **4**–**13**; Table S3: ^1^H and ^13^C NMR chemical shifts (TMS, ppm) for **3**, obtained using GIAO//B3LYP-D3(BJ)/def2-TZVPP level of theory in DMSO with the help of the CPCM continuum solvation model; Table S4: ^1^H and ^13^C NMR chemical shifts (TMS, ppm) for **5**, obtained using GIAO//B3LYP-D3(BJ)/def2-TZVPP level of theory in DMSO with the help of the CPCM continuum solvation model; Table S5: ^1^H and ^13^C NMR chemical shifts (TMS, ppm) for **6**, obtained using GIAO//B3LYP-D3(BJ)/def2-TZVPP level of theory in DMSO with the help of the CPCM continuum solvation model; Table S6: ^1^H and ^13^C NMR chemical shifts (TMS, ppm) for **6**, obtained using GIAO//B3LYP-D3(BJ)/def2-TZVPP level of theory in DMSO with the help of the CPCM continuum solvation model; Table S7: ^1^H and ^13^C NMR chemical shifts (TMS, ppm) for **8**, obtained using GIAO//B3LYP-D3(BJ)/def2-TZVPP level of theory in DMSO with the help of the CPCM continuum solvation model; Table S8: ^1^H and ^13^C NMR chemical shifts (TMS, ppm) for **9**, obtained using GIAO//B3LYP-D3(BJ)/def2-TZVPP level of theory in DMSO with the help of the CPCM continuum solvation model; Table S9: ^1^H and ^13^C NMR chemical shifts (TMS, ppm) for **10**, obtained using GIAO//B3LYP-D3(BJ)/def2-TZVPP level of theory in DMSO with the help of the CPCM continuum solvation model; Table S10: ^1^H and ^13^C NMR chemical shifts (TMS, ppm) for **11**, obtained using GIAO//B3LYP-D3(BJ)/def2-TZVPP level of theory in DMSO with the help of the CPCM continuum solvation model; Table S11: ^1^H and ^13^C NMR chemical shifts (TMS, ppm) for **12**, obtained using GIAO//B3LYP-D3(BJ)/def2-TZVPP level of theory in DMSO with the help of the CPCM continuum solvation model; Table S12: ^1^H and ^13^C NMR chemical shifts (TMS, ppm) for **13**, obtained using GIAO//B3LYP-D3(BJ)/def2-TZVPP level of theory in DMSO with the help of the CPCM continuum solvation model; Table S13: Cartesian coordinates of optimized geometries for configurations and conformations of **4**, obtained using B3LYP-D3(BJ)/def2-TZVPP level of theory in DMSO with the help of the CPCM continuum solvation model; Table S14: Cartesian coordinates of optimized geometries for configurations and conformations of **5**, obtained using B3LYP-D3(BJ)/def2-TZVPP level of theory in DMSO with the help of the CPCM continuum solvation model; Table S15: Cartesian coordinates of optimized geometries for configurations and conformations of **6**, obtained using B3LYP-D3(BJ)/def2-TZVPP level of theory in DMSO with the help of the CPCM continuum solvation model; Table S16: Cartesian coordinates of optimized geometries for configurations and conformations of **7**, obtained using B3LYP-D3(BJ)/def2-TZVPP level of theory in DMSO with the help of the CPCM continuum solvation model; Table S17: Cartesian coordinates of optimized geometries for configurations and conformations of **8**, obtained using B3LYP-D3(BJ)/def2-TZVPP level of theory in DMSO with the help of the CPCM continuum solvation model; Table S18: Cartesian coordinates of optimized geometries for configurations and conformations of **9**, obtained using B3LYP-D3(BJ)/def2-TZVPP level of theory in DMSO with the help of the CPCM continuum solvation model; Table S19: Cartesian coordinates of optimized geometries for configurations and conformations of **10**, obtained using B3LYP-D3(BJ)/def2-TZVPP level of theory in DMSO with the help of the CPCM continuum solvation model; Table S20: Cartesian coordinates of optimized geometries for configurations and conformations of **11**, obtained using B3LYP-D3(BJ)/def2-TZVPP level of theory in DMSO with the help of the CPCM continuum solvation model; Table S21: Cartesian coordinates of optimized geometries for configurations and conformations of **12**, obtained using B3LYP-D3(BJ)/def2-TZVPP level of theory in DMSO with the help of the CPCM continuum solvation model; Table S22: Cartesian coordinates of optimized geometries for configurations and conformations of **13**, obtained using B3LYP-D3(BJ)/def2-TZVPP level of theory in DMSO with the help of the CPCM continuum solvation model.
