# Supplementary material for: Synthesis, Structure and Biological Activity of 2-Methyl-5-nitro-6-phenylnicotinohydrazide-Based Hydrazones
Source: Molecules. 2025 Jan 4;30(1):169. doi: 10.3390/molecules30010169 (PMC11721005; doi:10.3390/molecules30010169)
Supplement: Supplementary file 1 [file molecules-30-00169-s001.zip › molecules-3374906-supplementary.pdf]

Electronic Supporting Information

Synthesis, Structure and Biological Activity of 2-Methyl-5-Nitro-6-Phenylnicotinohydrazide-based Hydrazones

Table of Contents

1 General Experimental Procedures 4
1.1 Instrumentation 4
1.2 Synthetic procedure for compound 3 4
1.3 General synthetic procedure for compounds 4–13 4

2 NMR spectra 8

3 DFT calculations 31
3.1 Analysis of thermodynamic data for configurations and conformations 31
3.2 NMR summary data for configurations and conformations 32
3.3 Cartesian coordinates of optimized geometries 42

List of Figures

S1 ^{1}H NMR (400 MHz, DMSO-d_6) and ^{13}C NMR (100 MHz, DMSO-d_6) spectra of **3**.
S2.1 ^{1}H NMR (400 MHz, DMSO-d_6) and ^{13}C NMR (100 MHz, DMSO-d_6) spectra of **4**.
S2.2 ^{1}H-^{1}H COSY spectra of **4**
S2.3 HMQC spectra of **4**
S2.4 HMBC spectra of **4**
S3.1 ^{1}H NMR (400 MHz, DMSO-d_6) and ^{13}C NMR (100 MHz, DMSO-d_6) spectra of **5**.
S3.2 ^{1}H-^{1}H COSY spectra of **5**
S3.3 HMQC spectra of **5**
S3.4 HMBC spectra of **5**
S4.1 ^{1}H NMR (400 MHz, DMSO-d_6) and ^{13}C NMR (100 MHz, DMSO-d_6) spectra of **6**.
S4.2 ^{1}H-^{1}H COSY spectra of **6**
S4.3 HMQC spectra of **6**
S4.4 HMBC spectra of **6**
S5.1 ^{1}H NMR (400 MHz, DMSO-d_6) and ^{13}C NMR (100 MHz, DMSO-d_6) spectra of **7**.
S5.2 ^{1}H-^{1}H COSY spectra of **7**
S5.3 HMQC spectra of **7**
S5.4 HMBC spectra of **7**
S6 ^{1}H NMR (400 MHz, DMSO-d_6) and ^{13}C NMR (100 MHz, DMSO-d_6) spectra of **8**.
S7 ^{1}H NMR (400 MHz, DMSO-d_6) and ^{13}C NMR (100 MHz, DMSO-d_6) spectra of **9**.
S8 ^{1}H NMR (400 MHz, DMSO-d_6) and ^{13}C NMR (100 MHz, DMSO-d_6) spectra of **10**.
S9 ^{1}H NMR (400 MHz, DMSO-d_6) and ^{13}C NMR (100 MHz, DMSO-d_6) spectra of **11**.
S10.1 ^{1}H NMR (400 MHz, DMSO-d_6) and ^{13}C NMR (100 MHz, DMSO-d_6) spectra of **12**.
S10.2 ^{1}H-^{1}H COSY spectra of **12**
S10.3 HMQC spectra of **12**
S10.4 HMBC spectra of **12**
S11.1 ^{1}H NMR (400 MHz, DMSO-d_6) and ^{13}C NMR (100 MHz, DMSO-d_6) spectra of **13**.
S11.2 ^{1}H-^{1}H COSY spectra of 13
S11.3 HMQC spectra of 13
S11.4 HMBC spectra of 13
S12 2D structures of possible configurations and conformations for **4–13**.
S13 Optimized geometry of **4**, obtained using B3LYP-D3(BJ)/def2-TZVPP level of theory in DMSO with the help of the CPCM continuum solvation model.

S14 Optimized geometry of **5**, obtained using B3LYP-D3(BJ)/def2-TZVPP level of theory in DMSO with the help of the CPCM continuum solvation model.
S15 Optimized geometry of **6**, obtained using B3LYP-D3(BJ)/def2-TZVPP level of theory in DMSO with the help of the CPCM continuum solvation model.
S16 Optimized geometry of **7**, obtained using B3LYP-D3(BJ)/def2-TZVPP level of theory in DMSO with the help of the CPCM continuum solvation model.
S17 Optimized geometry of **8**, obtained using B3LYP-D3(BJ)/def2-TZVPP level of theory in DMSO with the help of the CPCM continuum solvation model.
S18 Optimized geometry of **9**, obtained using B3LYP-D3(BJ)/def2-TZVPP level of theory in DMSO with the help of the CPCM continuum solvation model.
S19 Optimized geometry of **10**, obtained using B3LYP-D3(BJ)/def2-TZVPP level of theory in DMSO with the help of the CPCM continuum solvation model.
S20 Optimized geometry of **11**, obtained using B3LYP-D3(BJ)/def2-TZVPP level of theory in DMSO with the help of the CPCM continuum solvation model.
S21 Optimized geometry of **12**, obtained using B3LYP-D3(BJ)/def2-TZVPP level of theory in DMSO with the help of the CPCM continuum solvation model.
S22 Optimized geometry of **13**, obtained using B3LYP-D3(BJ)/def2-TZVPP level of theory in DMSO with the help of the CPCM continuum solvation model.

List of Tables

S.1 Gibbs free energy, Δ*G* (kcal/mol) differences in values for corresponding configurations and conformations of **4–13.**
S.2 Conformational constants, *K* for II→I-transition and equilibrium content of **4–13**.
S.3 ^{1}H and ^{13}C NMR chemical shifts (TMS, ppm) for **4**, obtained using GIAO//B3LYP-D3(BJ)/def2-TZVPP level of theory in DMSO with the help of the CPCM continuum solvation model.
S.4 ^{1}H and ^{13}C NMR chemical shifts (TMS, ppm) for **5**, obtained using GIAO//B3LYP-D3(BJ)/def2-TZVPP level of theory in DMSO with the help of the CPCM continuum solvation model.
S.5 ^{1}H and ^{13}C NMR chemical shifts (TMS, ppm) for **6**, obtained using GIAO//B3LYP-D3(BJ)/def2-TZVPP level of theory in DMSO with the help of the CPCM continuum solvation model.
S.6 ^{1}H and ^{13}C NMR chemical shifts (TMS, ppm) for **7**, obtained using GIAO//B3LYP-D3(BJ)/def2-TZVPP level of theory in DMSO with the help of the CPCM continuum solvation model.
S.7 ^{1}H and ^{13}C NMR chemical shifts (TMS, ppm) for **8**, obtained using GIAO//B3LYP-D3(BJ)/def2-TZVPP level of theory in DMSO with the help of the CPCM continuum solvation model.
S.8 ^{1}H and ^{13}C NMR chemical shifts (TMS, ppm) for **9**, obtained using GIAO//B3LYP-D3(BJ)/def2-TZVPP level of theory in DMSO with the help of the CPCM continuum solvation model.
S.9 ^{1}H and ^{13}C NMR chemical shifts (TMS, ppm) for **10**, obtained using GIAO//B3LYP-D3(BJ)/def2-TZVPP level of theory in DMSO with the help of the CPCM continuum solvation model.
S.10 ^{1}H and ^{13}C NMR chemical shifts (TMS, ppm) for **11**, obtained using GIAO//B3LYP-D3(BJ)/def2-TZVPP level of theory in DMSO with the help of the CPCM continuum solvation model.
S.11 ^{1}H and ^{13}C NMR chemical shifts (TMS, ppm) for **12**, obtained using GIAO//B3LYP-D3(BJ)/def2-TZVPP level of theory in DMSO with the help of the CPCM continuum solvation model.
S.12 ^{1}H and ^{13}C NMR chemical shifts (TMS, ppm) for **13**, obtained using GIAO//B3LYP-D3(BJ)/def2-TZVPP level of theory in DMSO with the help of the CPCM continuum solvation model.
S.13 Cartesian coordinates of optimized geometries for configurations and conformations of

4, obtained using B3LYP-D3(BJ)/def2-TZVPP level of theory in DMSO with the help of the CPCM continuum solvation model.
S.14 Cartesian coordinates of optimized geometries for configurations and conformations of **5**, obtained using B3LYP-D3(BJ)/def2-TZVPP level of theory in DMSO with the help of the CPCM continuum solvation model.
S.15 Cartesian coordinates of optimized geometries for configurations and conformations of **6**, obtained using B3LYP-D3(BJ)/def2-TZVPP level of theory in DMSO with the help of the CPCM continuum solvation model.
S.16 Cartesian coordinates of optimized geometries for configurations and conformations of **7**, obtained using B3LYP-D3(BJ)/def2-TZVPP level of theory in DMSO with the help of the CPCM continuum solvation model.
S.17 Cartesian coordinates of optimized geometries for configurations and conformations of **8**, obtained using B3LYP-D3(BJ)/def2-TZVPP level of theory in DMSO with the help of the CPCM continuum solvation model.
S.18 Cartesian coordinates of optimized geometries for configurations and conformations of **9**, obtained using B3LYP-D3(BJ)/def2-TZVPP level of theory in DMSO with the help of the CPCM continuum solvation model.
S.19 Cartesian coordinates of optimized geometries for configurations and conformations of **10**, obtained using B3LYP-D3(BJ)/def2-TZVPP level of theory in DMSO with the help of the CPCM continuum solvation model.
S.20 Cartesian coordinates of optimized geometries for configurations and conformations of **11**, obtained using B3LYP-D3(BJ)/def2-TZVPP level of theory in DMSO with the help of the CPCM continuum solvation model.
S.21 Cartesian coordinates of optimized geometries for configurations and conformations of **12**, obtained using B3LYP-D3(BJ)/def2-TZVPP level of theory in DMSO with the help of the CPCM continuum solvation model.
S.22 Cartesian coordinates of optimized geometries for configurations and conformations of **13**, obtained using B3LYP-D3(BJ)/def2-TZVPP level of theory in DMSO with the help of the CPCM continuum solvation model.

1 General Experimental Procedures

1.1 Instrumentation

^1H and ^{13}C NMR spectra were acquired on a Jeol JNM-ECA 400 spectrometer (400 and 100 MHz, respectively) and Magritek spinsolve 80 carbon ultra (81 and 20 MHz, respectively) in DMSO-d_6, using TMS or residual solvent signals as internal standard. The elemen- tal analysis (C,H,N) was performed on the EuroVector Elemental Analyser device. Melting points were determined using the SMP 10 device. TLC analysis was performed on Silufol UV-254 plates, manifested with iodine vapor.

1.2 Synthetic procedure for compound 3

To a solution of 2.86 g (10 mmol) ethyl ether of 2-methyl-5-nitro-6- phenylnicotinic acid (**2b**) in 50 ml of 2-PrOH, 5 ml of 80% hydrazine hydrate is added and heated with intensive stirring at a temperature of 60–70 °C for about an hour. Upon cooling, white lamellar crystals fall out of the flask, which are filtered, washed with cold 2-PrOH and dried.

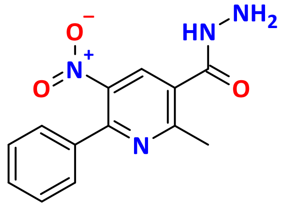

2-Methyl-5-nitro-6-phenylnicotinohydrazide (**3**). White crystals (2.67 g, 98%), mp 222–224 °C. IR spectrum, ν, cm^{-1}: 664; 694; 760; 926; 980; 1180; 1319; 1346, 1516 (–NO_2); 1366; 1443; 1555; 1597; 1647(C=O); 1744; 2855; 2924; 3052; 3187; 3275 (N–H). ^1H NMR (80 MHz, DMSO-d_6) δ 9.81 (br s, 1H, NH), 8.35 (s, 1H, H-4), 7.51 (s, 5H, Ph), 4.61 (br s, 2H, NH_2), 2.66 (s, 3H, CH_3); ^{13}C NMR (20 MHz, DMSO-d_6) δ 164.7 (C=O), 159.7 (C-2), 151.1 (C-6), 143.3 (C-5), 135.9 (C-3), 132.0 (C-4), 129.7 (C-4'), 129.6 (C-1'), 128.6 (C-3', C-5'), 128.0 (C-2', C-6'), 22.9 (CH_3). Anal. calcd for $C_{13}H_{12}N_4O_3$: C, 57.35; H, 4.44; N, 20.58; found: C, 57.02; H, 4.72; N, 20.75.

1.3 General synthetic procedure for compounds 4–13

To a mixture of 0.5 g (1.8 mmol) of **3** in 10 ml of EtOH, 1.8 mmol of the corresponding aromatic aldehyde was added with stirring. The reaction mixture was stirred at a temperature of 60–70 °C for 6 hours, then cooled to room temperature. The raw product was filtered, washed with cold alcohol and dried. The precipitate was recrystallized several times from 2-PrOH.

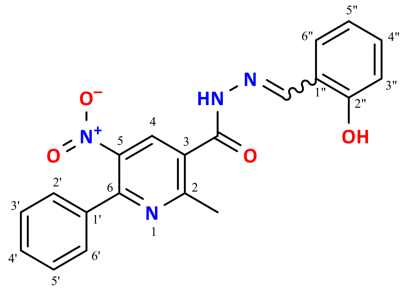

(*E*-,*Z*-)-*N*'-(2-hydroxybenzylidene)-2-methyl-5-nitro-6-phenylnicotinohydrazide (3 : 1) (**4**). White crystals (0.54 g, 76%), mp 272–273 °C. IR spectrum, ν, cm^{-1}: 694; 760; 856; 964; 1034; 1150; 1204; 1273; 1300; 1365, 1508 (–NO_2); 1439; 1551; 1636 (C=N); 1659 (C=O); 2851; 2920; 3052; 3229 (N–H); 3420 (–OH). ^1H NMR (400 MHz, DMSO-d_6) δ 12.28 (br s, 1H, NH), 11.00 (br s, 1H, OH), 8.65 (s, 0.75H, =CH (*E*-)), 8.56 (s, 0.75H, H-4 (*E*-)), 8.52 (s, 0.25H, =CH (*Z*-)), 8.45 (s, 0.25H, H-4 (*Z*-)), 7.45–7.65 (m, 5H, Ph), 7.17–7.40 (m, 2H, H-4",6" Ar (*E*- + *Z*-)), 6.90–7.00 (m, 2H, H-3",5" Ar (*E*- + *Z*-)), 2.74 (s, 2.2H, CH_3 (*E*-)), 2.60 (s, 0.8H, CH_3 (*Z*-)); 13C NMR (100 MHz, DMSO-d_6) δ 167.1 (C=O), 161.4, 160.3, 157.5 (C-2" Ar), 151.7 (C-6 (*E*-)), 150.9 (C-6 (*Z*-)), 148.6 (=CH), 143.5 (C-5 (*Z*-)), 143.3 (C-5 (*E*-)), 136.0 (C-3 (*Z*-)), 135.9 (C-3 (*E*-)), 132.5 (C-4 (*E*-)), 132.4 (C-4 (*Z*-)), 131.8 (C-4" Ar (*E*-)), 131.5 (C-4" Ar (*Z*-)), 129.9 (C-1' Ph (*E*-)), 129.7 (C-1' Ph (*Z*-)), 129.1 (C-4' Ph), 128.7 (C-3',5' Ph), 128.1 (C-2',6' Ph), 119.5 (C-5" Ar), 118.7 (C-1" Ar), 116.4 (C-3" Ar), 23.1 (CH_3 (*E*-)), 22.9 (CH_3 (*Z*-)). Anal. calcd for $C_{20}H_{16}N_4O_4$: C, 63.82; H, 4.28; N, 14.89; found: C, 63.31; H, 4.14; N, 14.77.

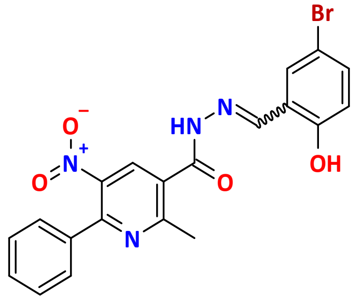

(*E*-,*Z*-)-*N′*-(5-bromo-2-hydroxybenzylidene)-2-methyl-5-nitro-6-phenylnicotinohydrazide (7 : 3) (**5**). White crystals (0.85 g, 97%), mp 286–288 °C. IR spectrum, ν, cm^{-1}: 628 (C-Br); 694; 760; 834; 910; 976; 1030; 1115; 1154; 1188; 1273; 1343, 1551 (–NO_2); 1443; 1474; 1508; 1601 (C=N); 1659 (C=O); 2855; 2924; 3036; 3198; 3333 (–OH). ^{1}H NMR (400 MHz, DMSO-d_6) δ 10.13 (br s, 1H, NH), 9.64 (br s, 1H, OH), 8.58 (s, 0.7H, H-4 (*E*-)), 8.48 (s, 0.3H, H-4 (*Z*-)), 8.48 (s, 0.7H, =CH (*E*-)), 8.32 (s, 0.3H, =CH (*Z*-)), 7.81 (s, 0.7H, H-6” Ar (*E*-)), 7.48–7.55 (m, 5H Ph, 0.3H, H-6” Ar (*Z*-)), 7.42 (d, 0.7H, *J* = 7.8 Hz, H-4” Ar (*E*-)), 7.35 (d, 0.3H, *J* = 8.0 Hz, H-4” Ar (*Z*-)), 6.89 (d, 0.7H, *J* = 8.7 Hz, H-3” Ar (*E*-)), 6.80 (d, 0.3H, *J* = 8.9 Hz, H-3” Ar (*Z*-)), 2.70 (s, 2H, CH_3 (*E*-)), 2.57 (s, 1H, CH_3 (*Z*-)); 13C NMR (100 MHz, DMSO-d_6) δ 167.9 (C=O), 162.0, 160.6, 152.1, 146.4 (*Z*-), 143.5 (*Z*-), 139.1 (*Z*-), 136.1 (*E*-), 134.4 (*Z*-), 132.8 (*E*-), 130.3 (*Z*-), 129.0 (2C), 128.4 (2C), 128.3, 125.3, 121.5, 119.0 (*E*-), 110.9 (*Z*-), 23.4 (CH_3). Anal. calcd for $C_{20}H_{15}BrN_4O_4$: C, 52.76; H, 3.32; N, 12.31; found: C, 53.04; H, 3.14; N, 12.24.

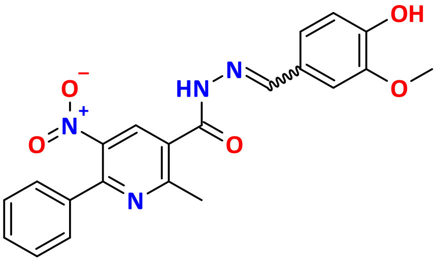

(*E*-,*Z*-)-*N′*-(4-hydroxy-3-methoxybenzylidene)-2-methyl-5-nitro-6-phenylnicotinohydrazide (7 : 3) (**6**). White crystals (0.62 g, 80%), mp 260–262 °C. IR spectrum, ν, cm^{-1}: 694; 756; 899; 868; 1026; 1165 (–OCH_3); 1208; 1277; 1346, 1512 (–NO_2); 1555; 1601 (C=N); 1655 (C=O); 2361; 2855 (–OCH_3); 2928; 3059; 3210 (N–H); 3433 (–OH). ^{1}H NMR (400 MHz, DMSO-d_6) δ 11.98 (br s, 1H, NH), 9.81 (br s, 1H, OH), 8.53 (s, 0.7H, H-4 (*E*-)), 8.48 (s, 0.3H, H-4 (*Z*-)), 8.17 (s, 0.7H, =CH (*E*-)), 7.99 (s, 0.3H, =CH (*Z*-)), 7.38 (s, 1H, H-2’’ Ar), 7.42–7.59 (m, 5H, Ph), 6.93–7.17 (m, 1H, H-6’’, Ar (*E*- + *Z*-)), 6.85 (br. s, 0.7H, H-5’’ (*E*-) Ar), 6.75 (br.s, 0.3H, H-5’’ (*Z*-) Ar), 3.81 (s, 3H, OCH_3), 2.68 (s, 2H, CH_3 (*E*-)), 2.58 (s, 1H, CH_3 (*Z*-)); ^{13}C NMR (100 MHz, DMSO-d_6) δ 167.3 (C=O (*E*-)), 167.2 (C=O (*Z*-)), 161.9, 160.3, 151.9, 149.7 (*E*-), 149.6 (*Z*-), 148.4 (*E*-), 148.2 (*Z*-), 143.6 (*E*-), 143.5 (*Z*-), 136.3 (*Z*-), 136.2 (*E*-), 133.1 (*Z*-), 132.5 (*E*-), 130.2 (*E*-), 130.1 (*Z*-), 129.5, 129.0 (2C), 128.3 (2C), 125.5, 123.0, 115.7, 109.3 (*E*-), 109.0 (*Z*-), 55.8 (OCH_3 (*E*-)), 55.3 (OCH_3 (*Z*-)), 23.2 (CH_3). Anal. calcd for $C_{21}H_{18}N_4O_5$: C, 62.06; H, 4.46; N, 13.79; found: C, 62.49; H, 4.23; N, 13.96.

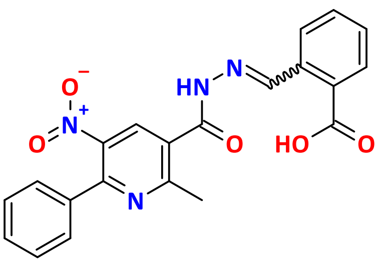

(*E*-,*Z*-))-2-((2-(2-methyl-5-nitro-6-phenylnicotinoyl)hydrazono)methyl) benzoic acid (7 : 3) (**7**). White crystals (0.66 g, 86%), mp 235–237 °C. IR spectrum, ν, cm^{-1}: 691; 752; 833; 922; 945; 1034; 1076; 1154; 1281; 1350, 1520 (–NO_2); 1393; 1451; 1551; 1601 (C=N); 1678 (C=O); 2508; 2635; 2928; 2962; 3052; 3437 (–OH). ^{1}H NMR (400 MHz, DMSO-d_6) δ 12.36 (br s, 0.3H, NH (*Z*-)), 12.32 (br s, 0.7H, NH (*E*-)), 9.07 (s, 0.7H, =CH (*E*-)), 8.87 (s, 0.3H, =CH (*Z*-)), 8.58 (s, 0.7H, H-4 (*E*-)), 8.48 (s, 0.3H, H-4 (*Z*-)), 8.10 (d, 0.7H, *J* = 6.8 Hz, H-3’ Ar (*E*-)), 7.84 (d, 0.3H, *J* = 6.7 Hz, H-3’ Ar (*Z*-)), 7.91 (d, 0.7H, *J* = 7.4 Hz, H-6’ Ar (*E*-)), 7.66 (d, 0.3H, *J* = 6.9 Hz, H-6’ Ar (*Z*-)), 7.64 (t, 0.7H, *J* = 5.9 Hz, H-5’ Ar (*E*-)), 7.46–7.56 (m, 5H, Ph, 1H, H-4’ Ar (*E*- + *Z*-), 0.3H, H-5’ Ar (*Z*-)), 2.70 (s, 2H, CH_3 (*E*-)), 2.59 (s, 1H, CH_3 (*Z*-)); ^{13}C NMR (100 MHz, DMSO-d_6) δ 168.4 (*E*-), 168.3 (*Z*-), 162.4, 160.5, 152.0 (*E*-), 151.3 (*Z*-), 148.2, 143.7 (*Z*-), 143.6 (*E*-), 136.2 (*Z*-), 136.1 (*E*-), 134.4 (*E*-), 134.2 (*Z*-), 132.9 (*Z*-), 132.8 (*E*-), 132.5 (*E*-), 132.4 (*Z*-), 131.1 (*E*-), 131.0 (*Z*-), 130.8 (*E*-), 130.7 (*Z*-), 130.5, 130.3 (*E*-), 130.2 (*Z*-), 129.2, 129.1 (1C (*E*-)), 129.0 (3C (*Z*-)), 128.43 (1C (*Z*-)), 128.37 (3C (*E*-)), 127.1 (*E*-), 126.6 (*Z*-, 23.3 (CH_3 (*E*-)), 23.2 (CH_3 (*Z*-)). Anal. calcd for $C_{21}H_{16}N_4O_5$: C, 62.37; H, 3.99; N, 13.86; found: C, 62.28; H, 4.15; N, 13.95.

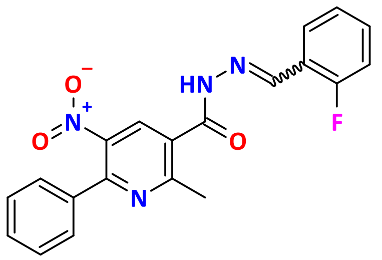

(*E*-,*Z*-)-*N'*-(2-fluorobenzylidene)-2-methyl-5-nitro-6-phenylnicotino-hydrazide (3:2) (**8**).White crystals (0.52 g, 74%), mp 283–285 °C. IR spectrum, ν, cm^{-1}: 698; 760; 772; 837; 887; 937; 961; 1061; 1096 (C–F); 1300; 1343, 1520 (–NO_2); 1370; 1451; 1481; 1551; 1598 (C=N); 1651 (C=O); 1709; 2855; 2924; 2924; 2967; 3168; 3275 (N–H). ^{1}H NMR (400 MHz, DMSO-d_6) δ 12.23 (br s, 1H, NH), 8.65 (br s, 0.6H, =CH (*E*-)), 8.53 (br s, 1 H, H-4 (*E*- + *Z*-)), 8.34 (br s, 0.4H, =CH (*Z*-)), 7.19–7.96 (m, 9H, 5H, Ph, H-2',3',4',5' Ar (*E*- + *Z*-)), 2.72 (s, 2H, CH_3 (*E*-)), 2.59 (s, 1H, CH_3 (*Z*-)); ^{13}C NMR (100 MHz, DMSO-d_6) δ 167.4 (C=O), 160.9 (d, $J_{13C\text{–}F}$ = 145 Hz, C-2''), 159.7, 158.5, 151.7, 143.4, 141.5, 138.4, 135.8, 132.75 (d, $^{3}J_{13C\text{–}F}$ = 8.6 Hz, C-4'' Ar), 132.5 (*E*- +*Z*-), 129.95 (d, $^{3}J_{13C\text{–}F}$ = 9.6 Hz, C-6'' Ar), 128.9, 128.7 (C-3',5' Ph), 128.1 (C-2',6' Ph), 125.1 (*Z*- + *E*-), 121.4 (d, $^{2}J_{13C\text{–}F}$ = 26 Hz, C-1''), 116.14 (d, $^{2}J_{13C\text{–}F}$ = 22 Hz, C-3'), 23.1 (CH_3). Anal. calcd for $C_{20}H_{15}FN_4O_3$: C, 63.49; H, 4.00; N, 14.81; found: C, 63.72; H, 4.27; N, 14.65.

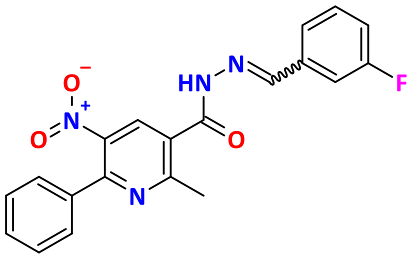

(*E*-,*Z*-)-*N'*-(3-fluorobenzylidene)-2-methyl-5-nitro-6-phenylnicotino-hydrazide (6.5:3.5) (**9**). White crystals (0.48 g, 68%), mp 257–259 °C. IR spectrum, ν, cm^{-1}: 691; 760; 837; 868; 980; 1134 (C–F); 1269; 1300; 1343, 1555 (–NO_2); 1443; 1516; 1612 (C=N); 1663 (C=O); 1740; 2338; 2361; 2859; 2924; 3063; 3198; 3275 (N–H). ^{1}H NMR (400 MHz, DMSO-d_6) δ 12.24 (br s, 1H, NH), 8.58 (s, 0.65H, H-4 (*E*-)), 8.49 (s, 0.35H, H-4 (*Z*-)), 8.31 (s, 0.65H, =CH (*E*-)), 8.13 (s, 0.35H, =CH (*Z*-)), 7.18–7.57 (m, 9H, 5H Ph, H-2',3',4',6' Ar (*E*- + *Z*-)), 2.71 (s, 2H, CH_3 (*E*-)), 2.58 (s, 1H, CH_3 (*Z*-)); ^{13}C NMR (100 MHz, DMSO-d_6) δ 167.7 (C=O), 161.4 (C-2 (*E*-)), 161.3 (C-2 (*Z*-)), 161.3 (d, $^{1}J_{13C\text{–}F}$ = 187 Hz, C-4''), 158.8, 152.0 (*E*-), 151.3 (*Z*-), 147.8, 144.3 (*E*-), 143.5 (*Z*-), 136.58 (d,$J_{13C\text{–}F}$ = 8 Hz, C-1' Ar), 136.1 (*Z*-), 136.0 (*E*-), 133.0 (C-4 (*Z*-)), 132.6 (C-4 (*E*-)), 131.27 (d, $^{3}J_{13C\text{–}F}$ = 8 Hz, C-5'' Ar), 130.2 (*E*-), 130.1 (*Z*-), 129.2, 129.0 (C-3',5' Ph), 128.3 (C-2',6' Ph), 124.0 (*E*-), 123.4 (*Z*-), 117.2 (d, $^{2}J_{13C\text{–}F}$ = 26 Hz, C-4''), 113.5 (d, 2$J_{13C\text{–}F}$ = 22 Hz, C-2''), 23.2 (CH_3 (*E*-)), 23.1 (CH_3 (*Z*-)). Anal. calcd for $C_{20}H_{15}FN_4O_3$: C, 63.49; H, 4.00; N, 14.81; found: C, 63.82; H, 4.15; N, 15.05.

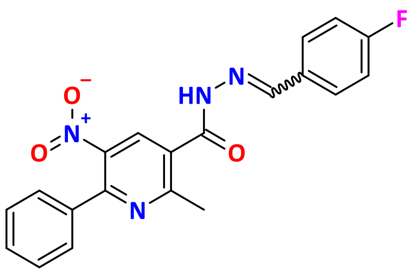

(*E*-,*Z*-)-*N'*-(4-fluorobenzylidene)-2-methyl-5-nitro-6-phenylnicotino-hydrazide (6.5:3.5) (**10**). White crystals (0.35 g, 50%), mp 268–270 °C. IR spectrum, ν, cm^{-1}: 610; 698; 760; 845; 880; 968; 1045; 1134 (C–F); 1157; 1235; 1292; 1346, 1512 (–NO_2); 1447; 1555; 1601 (C=N); 1667 (C=O); 2365; 2855; 2928; 2974; 3055; 3383 (N–H). ^{1}H NMR (400 MHz, DMSO-d_6) δ 12.16 (br s, 1H, NH), 8.57 (s, 0.65H, H-4 (*E*-)), 8.47 (s, 0.35H, H-4 (*Z*-)), 8.29 (s, 0.65H, =CH (*E*-)), 8.13 (s, 0.35H, =CH (*Z*-)), 7.81 (br s, 1.3H, H-2',6' Ar (*E*-)), 7.58 (br s, 0.7H, H-2',6' Ar (*Z*-)), 7.46–7.56 (m, 5H, Ph), 7.29 (br s, 1.3H, H-3',5' Ar (*E*-)), 7.19 (br s, 0.7H, H-3',5' Ar (*Z*-)), 2.70 (s, 2H, CH_3 (*E*-)), 2.58 (s, 1H, CH_3 (*Z*-)); ^{13}C NMR (100 MHz, DMSO-d_6) δ 162.5 (C=O), 161.4 (d, $^{1}J_{13C\text{–}F}$ = 185 Hz, C-4'), 158.9, 152.1, 148.3, 144.9 (*Z*-), 143.6 (*E*-), 136.3 (*Z*-), 136.2 (*E*-), 132.9 (*Z*-), 132.7 (*E*-), 130.7 (*E*-), 130.6 (*Z*-), 130.3 (*E*-), 130.2 (*Z*-), 130.0 (d, $^{3}J_{13C\text{–}F}$ = 8 Hz, C-2',6' Ar), 129.4, 129.1 (C-3,5 Ph), 128.4 (C-2,6 Ph), 116.4 (d, $^{2}J_{13C\text{–}F}$ = 24 Hz, C-3',5'), 23.3 (CH_3). Anal. calcd for $C_{20}H_{15}FN_4O_3$: C, 63.49; H, 4.00; N, 14.81; found: C, 63.32; H, 4.18; N, 15.12.

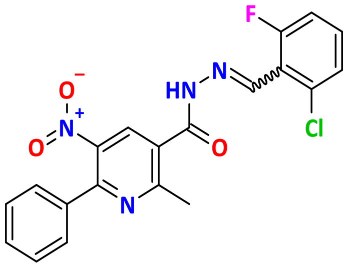

(*E*-,*Z*-)-*N'*-(2-chloro-6-fluorobenzylidene)-2-methyl-5-nitro-6-phenyl-nicotinohydrazide (4.5:5.5) (**11**). White crystals (0.64 g, 82%), mp 230–231 °C. IR spectrum, ν, cm^{-1}: 705; 760 (C–Cl); 1090; 1133 (C–F); 1345, 1565 (–NO2); 1620 (C=N); 1665 (C=O); 3227 (N–H). ^{1}H NMR (400 MHz, DMSO-d_6) δ 12.38 (br s, 1H, NH (*E*- + *Z*-)), 8.67 (s, 0.45H, =CH (*E*-)), 8.57 (s, 0.45H, H-4 (*E*-)), 8.47 (s, 0.55H, =CH (*Z*-)), 8.37 (s, 0.55H, H-4 (*Z*-)), 7.15–7.45 (m, 3H, H-3″,4″,5″ Ar (*E*- + *Z*-)), 7.45–7.60 (m, 5H, Ph), 2.73 (s, 1.3H, CH_3 (*E*-)), 2.60 (s, 1.7H, CH_3 (*Z*-)); ^{13}C NMR (100 MHz, DMSO-d_6) δ 167.7 (C=O), 160.1 (d, $^{1}J_{13C-F}$ = 315 Hz, C-1″), 160.3 (C-2 (*E*-)), 159.1 (C-2 (*Z*-)), 151.7 (C-6 (*E*-)), 150.9 (C-6 (*Z*-)), 143.3 (=CH (*E*-)), 143.2 (=CH (*Z*-)), 142.4 (C-5 (*E*-)), 142.2 ($^{3}J_{13C-F}$ = 16.3 Hz, C-5 (*Z*-)), 138.3 (d, C-2″ Ar), 135.9 (d, $^{3}J_{13C-F}$ = 17.2 Hz, C-4″ Ar), 134.1 (C-3 (*E*-)), 133.3 (C-3 (*Z*-)), 132.6 (C-4 (*E*-)), 132.5 (C-4 (*Z*-)), 130.0 (C-4′ Ph), 128.7 (C-3′,5′ Ph), 128.0 (C-2′,6′ Ph), 126.3 (C-3″ (*Z*-)), 126.1 (C-3″ (*E*-)), 119.8 (d, $^{2}J_{13C-F}$ = 13 Hz, C-1″), 115.66 (d, $^{2}J_{13C-F}$ = 22 Hz, C-5″), 23.1 (CH_3 (*E*-)), 22.8 (CH_3 (*Z*-)). Anal. calcd for $C_{20}H_{14}ClFN_4O_3$: C, 58.19; H, 3.42; N, 13.57; found: C, 57.92; H, 3.63 N, 12.97.

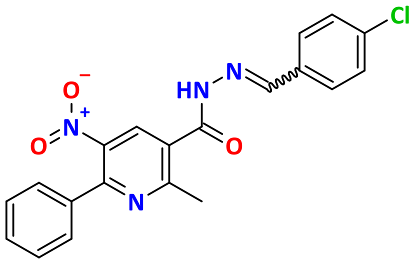

(*E*-,*Z*-)-*N′*-(4-chlorobenzylidene)-2-methyl-5-nitro-6-phenylnicotinohydrazide (6.5:3.5) (**12**). White crystals (0.41 g, 56%), mp 270–272 °C. IR spectrum, ν, cm^{-1}: 637; 691; 756 (C–Cl); 833; 934; 1092; 1269; 1339, 1555 (–NO_2); 1400; 1443; 1512; 1597; 1607 (C=N); 1659 (C=O); 2855; 2924; 3059; 3198 (N–H). ^{1}H NMR (400 MHz, DMSO-d_6) δ 12.31 (br s, 0.3H, NH (*Z*-)), 12.18 (br s, 0.7H, NH (*E*-)), 8.62 (s, 0.65H, H-4 (*E*-)), 8.51 (s, 0.35H, H-4 (*Z*-)), 8.31 (s, 0.65H, =CH (*E*-)), 8.14 (s, 0.35H, =CH (*Z*-)), 7.75–7.85 (m, 1.3H, H-2′,6′ Ar (*E*-)), 7.41–7.65 (m, 5H, Ph, 2H, H-3′,5′ Ar (*E*- + *Z*-), 0.7H, H-2′,6′ Ar (*Z*-)), 2.72 (s, 2H, CH_3 (*E*-)), 2.58 (s, 1H, CH_3 (*Z*-)); ^{13}C NMR (100 MHz, DMSO-d_6) δ 167.4 (C=O), 161.7 (C-2), 151.6 (C-6 (*E*-)), 151.0 (C-6 (*Z*-)), 147.5 (=CH), 144.2 (C-5 (*Z*-)), 143.4 (C-5 (*E*-)), 136.0 (C-4″ Ar (*Z*-)), 135.9 (C-4″ Ar (*E*-)), 135.0 (C-3 (*E*-)), 134.7 (C-3 (*Z*-)), 132.9 (C-1″ Ar (*E*-)), 132.7 (C-1″ Ar (*Z*-)), 132.6 (C-4 (*Z*-)), 132.4 (C-4, (*E*-)), 129.95 (C-1′ Ph (*E*-)), 129.80 (C-1′ Ph (*Z*-)), 129.04 (C-2″,6″ Ar), 128.98 (C-3″, 5″ Ar), 128.7 (C-3′,5′ Ph), 128.5 (C-4′ Ph), 128.1 (C-2′,6′ Ph), 23.3 (CH_3). Anal. calcd for $C_{20}H_{15}ClN_4O_3$: C, 60.84; H, 3.83; N, 14.19; found: C, 60.58; H, 3.97; N, 13.98.

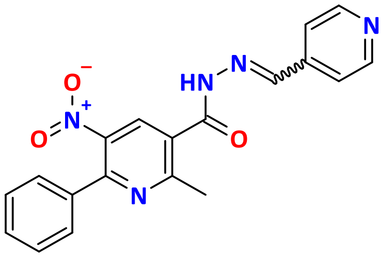

(*E*-,*Z*-)-2-methyl-5-nitro-6-phenyl-*N′*-(pyridin-4-ylmethylene)nicotinohydrazide (7:3) (**13**). White crystals (0.52 g, 77%), mp 272–273 °C. IR spectrum, ν, cm^{-1}: 694; 937; 976; 1072; 1111; 1142; 1343, 1512 (–NO_2); 1439; 1555; 1607 (C=N); 1670 (C=O); 1871; 1952; 2855; 2920; 3071; 3214 (N–H). ^{1}H NMR (400 MHz, DMSO-d_6) δ 12.39 (br s, 1H, NH (*E*- + *Z*-)), 8.67 (br s, 1.2H, H-2″,6″ Py (*E*-)), 8.64 (s, 0.7H, H-4 (*E*-)), 8.56 (br s, 0.8H, H-2″,6″ Py (*Z*-)), 8.53 (s, 0.3H, H-4 (*Z*-)), 8.31 (s, 0.65H, =CH (*E*-)), 8.13 (s, 0.35H, =CH (*Z*-)), 7.70 (br s, 1.2H, H-3″,5″ Py (*E*-)), 7.48–7.65 (m, 5H, Ph), 7.39 (br s, 0.8H, H-3″,5″ Py (*Z*-)), 2.73 (s, 1.9H, CH_3 (*E*-)), 2.60 (s, 1.1H, CH_3 (*Z*-)); ^{13}C NMR (100 MHz, DMSO-d_6) δ 167.7 (C=O), 162.0 (C-2 (*Z*-)), 160.1 (C-2 (*E*-)), 151.7 (C-6 (*E*-)), 151.1 (C-6 (*Z*-)), 150.3 (C-2″,6″ Py (*E*- + *Z*-)), 146.4 (=CH), 143.3 (C-5 (*E*-)), 143.0 (C-5 (*Z*-)), 141.1 (C-4″ Py (*E*-)), 140.8 (C-4″ Py (*Z*-)), 136.0 (C-3 (*Z*-)), 135.8 (C-3 (*E*-)), 132.8 (C-4 (*Z*-)), 132.5 (C-4 (*E*-)), 129.9 (C-1′ Ph (*E*-)), 129.8 (C-1′ Ph (*Z*-)), 128.8 (C-4′ Ph), 128.7 (C-3′,5′ Ph), 128.1 (C-2′,6′ Ph), 121.1 (C-3″,5″ Py (*E*-)), 120.7 (C-3″,5″ Py (*Z*-)), 23.0 (CH_3). Anal. calcd for $C_{19}H_{15}N_5O_3$: C, 63.15; H, 4.18; N, 19.38; found: C, 62.90; H, 4.39; N, 19.03.

2 NMR spectra

No.	(ppm)	(Hz)	Height
1	2.49	200.5	0.1237
2	2.66	214.2	0.6153
3	4.61	370.8	0.0474
4	7.51	604.4	1.0000
5	8.35	672.2	0.2232
6	9.81	789.8	0.0483

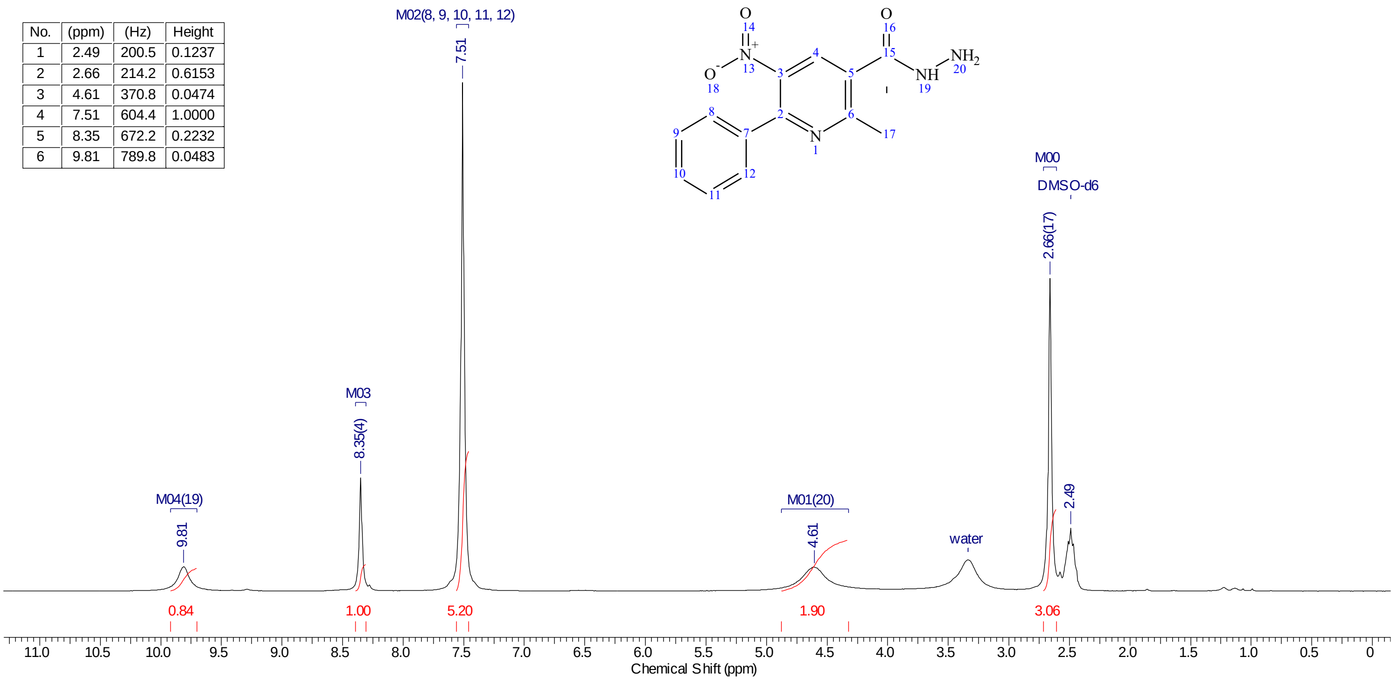

No.	(ppm)	(Hz)	Height
1	22.93	464.2	0.2601
2	128.00	2591.3	0.5707
3	128.60	2603.5	0.5828
4	129.62	2624.1	0.1606
5	129.73	2626.2	0.2480
6	131.95	2671.3	0.2293

No.	(ppm)	(Hz)	Height
7	135.92	2751.6	0.1408
8	143.26	2900.3	0.1171
9	151.12	3059.4	0.0797
10	159.69	3232.8	0.1312
11	164.72	3334.7	0.1195

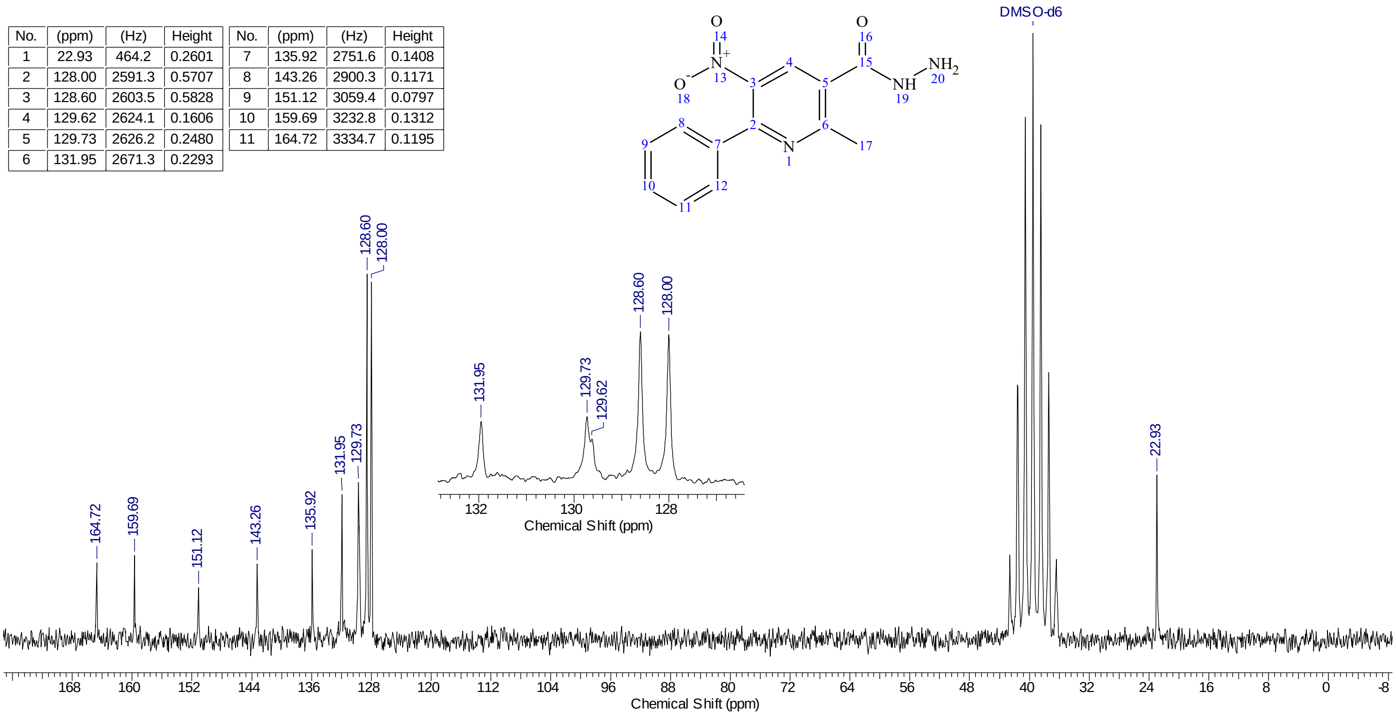

Figure S1: ^{1}H NMR (80 MHz, DMSO-d_6) and ^{13}C NMR (20 MHz, DMSO-d_6) spectra of **3**

No.	(ppm)	(Hz)	Height	No.	(ppm)	(Hz)	Height	No.	(ppm)	(Hz)	Height
1	2.60	1040.3	0.2842	9	7.17	2866.6	0.0333	17	8.45	3376.8	0.0964
2	2.74	1095.8	0.8870	10	7.19	2874.0	0.0630	18	8.52	3404.7	0.1072
3	6.84	2734.3	0.0540	11	7.21	2880.8	0.0602	19	8.56	3423.5	0.3216
4	6.86	2742.5	0.0783	12	7.29	2914.7	0.1018	20	8.65	3456.9	0.3127
5	6.90	2758.1	0.1121	13	7.31	2922.5	0.2068	21	11.00	4398.5	0.1234
6	6.92	2765.4	0.2056	14	7.33	2928.9	0.2329	22	12.28	4909.5	0.1241
7	6.94	2775.5	0.3074	15	7.51	3004.0	1.0000				
8	6.96	2783.7	0.3141	16	7.55	3019.6	0.6843				

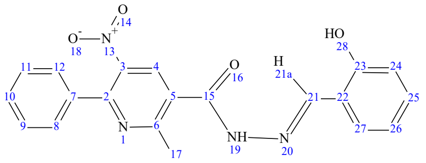

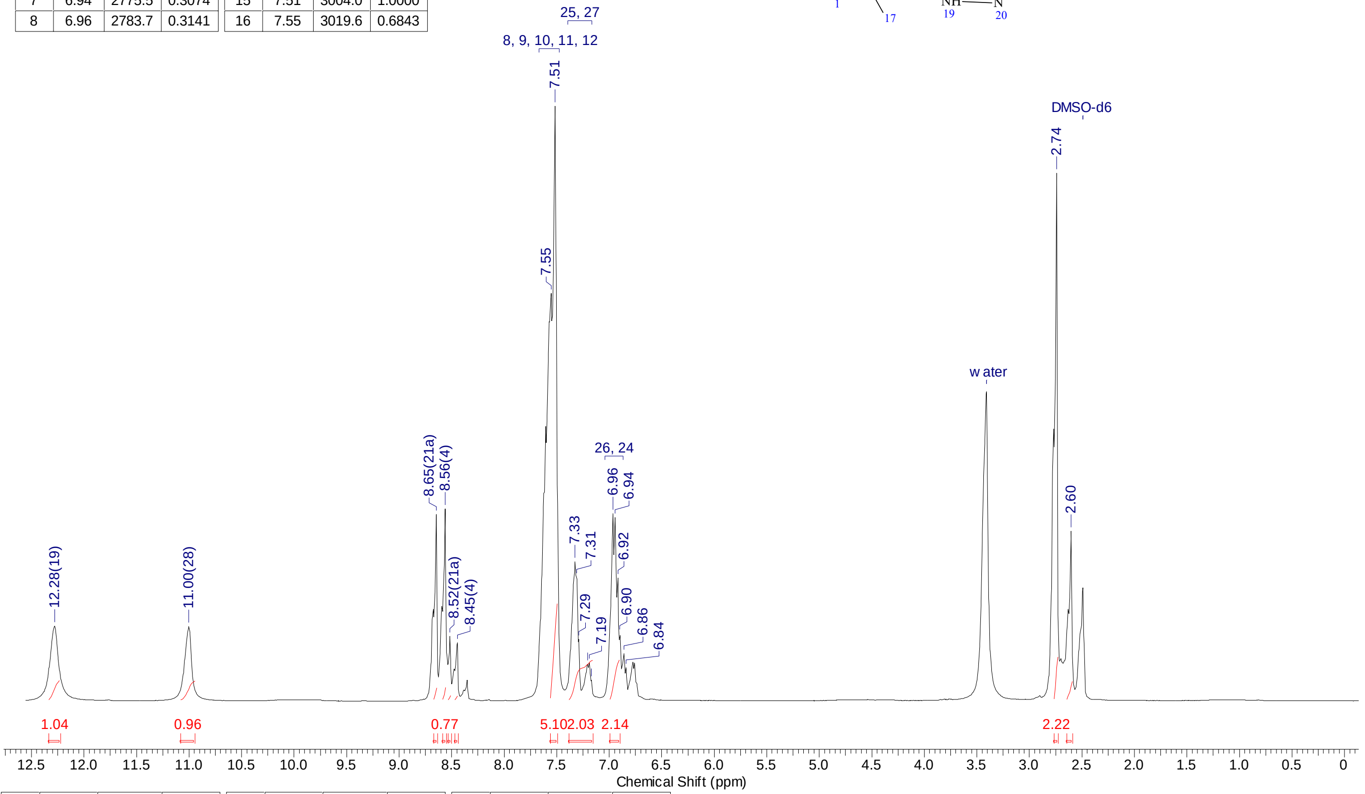

No.	(ppm)	(Hz)	Height	No.	(ppm)	(Hz)	Height	No.	(ppm)	(Hz)	Height
1	22.90	2302.1	0.0374	10	129.75	13043.6	0.0392	19	148.58	14935.6	0.0934
2	23.13	2325.1	0.1090	11	129.91	13058.9	0.0706	20	150.93	15172.3	0.0195
3	116.20	11680.7	0.0229	12	131.48	13217.1	0.0257	21	151.68	15248.1	0.0556
4	116.45	11706.5	0.0941	13	131.82	13251.6	0.0702	22	157.45	15827.9	0.0828
5	118.67	11928.9	0.0701	14	132.40	13309.1	0.0323	23	160.28	16112.6	0.0653
6	119.48	12010.4	0.1280	15	132.49	13318.7	0.0982	24	161.40	16224.7	0.0664
7	128.10	12876.8	0.2726	16	135.86	13657.0	0.0634	25	167.05	16793.1	0.0199
8	128.68	12935.3	0.3023	17	143.31	14406.5	0.0779				
9	129.06	12973.6	0.0886	18	143.47	14422.8	0.0352				

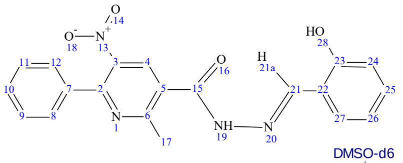

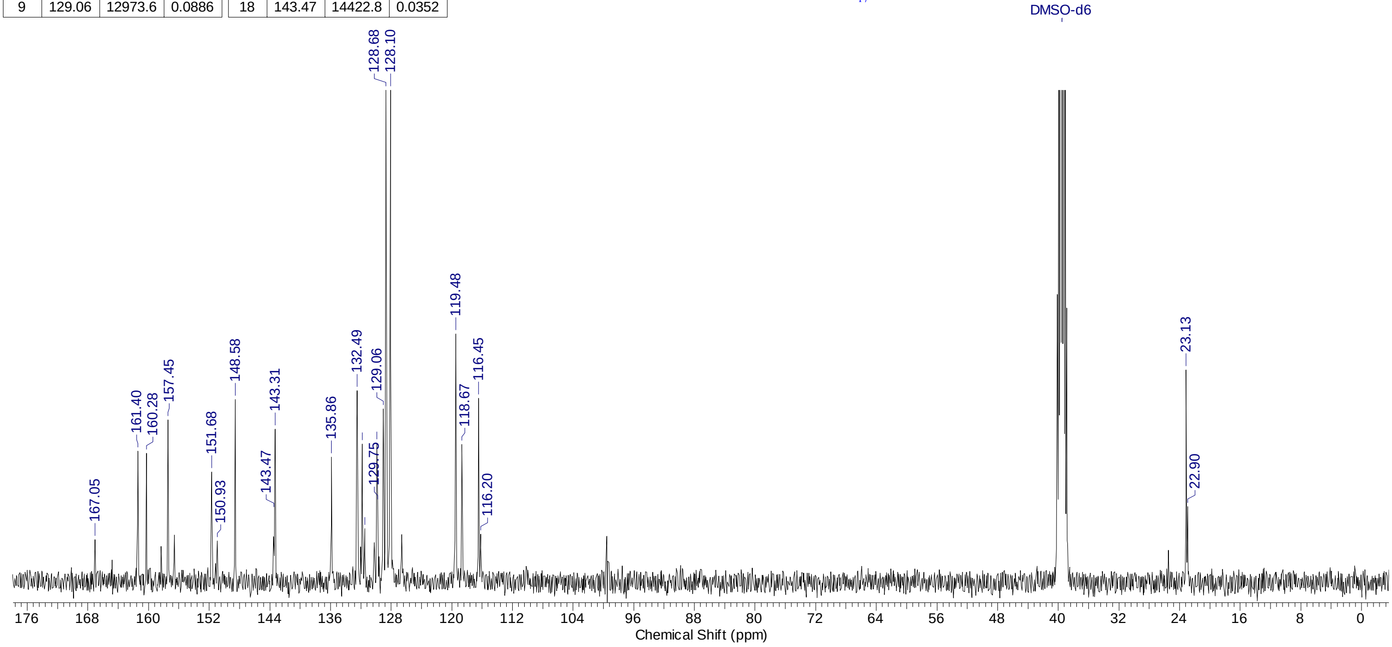

Figure S2.1: ^{1}H NMR (400 MHz, DMSO-d_6) and ^{13}C NMR (100 MHz, DMSO-d_6) spectra of **4**

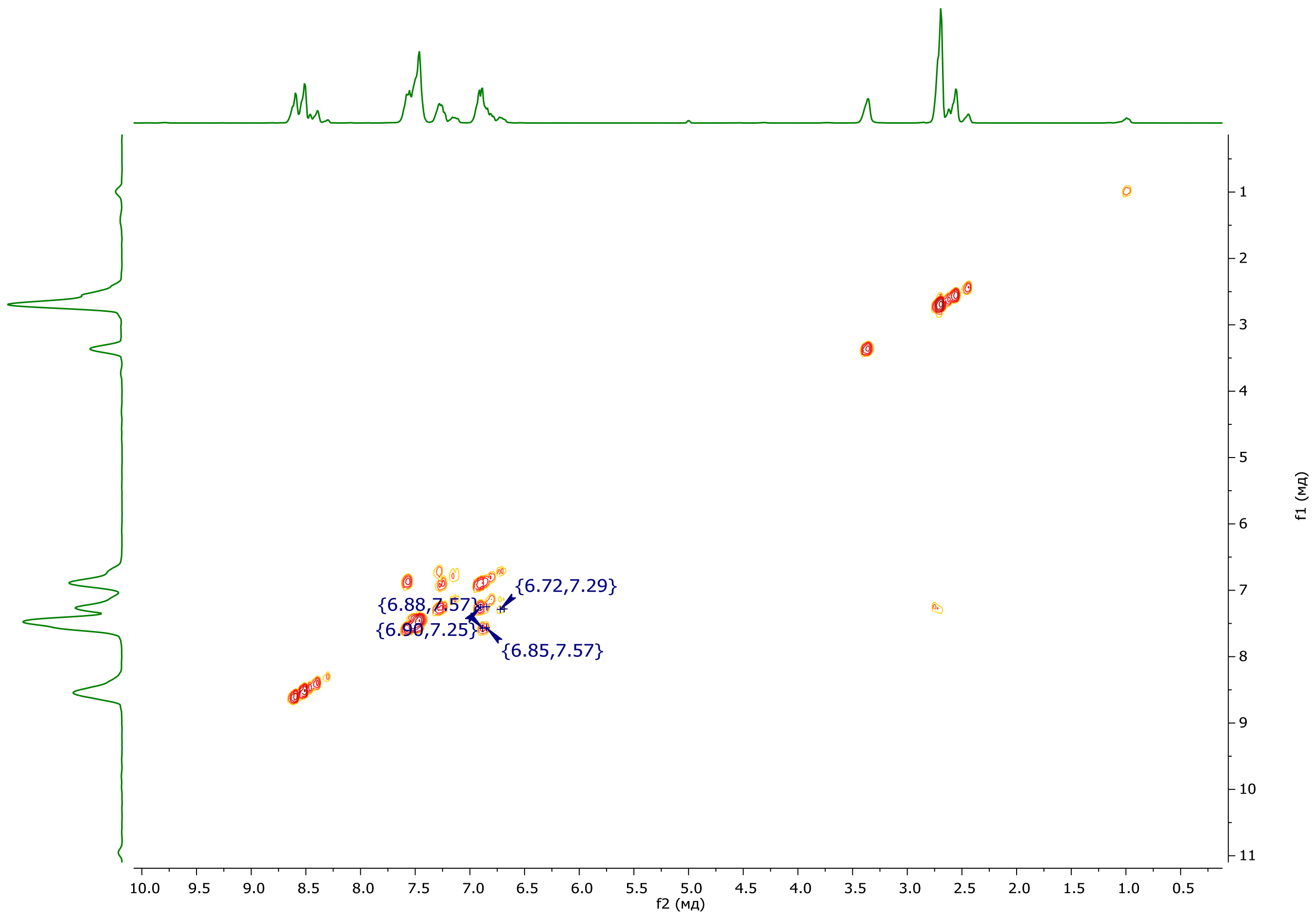

Figure S2.2: ^{1}H-^{1}H COSY spectra of **4**

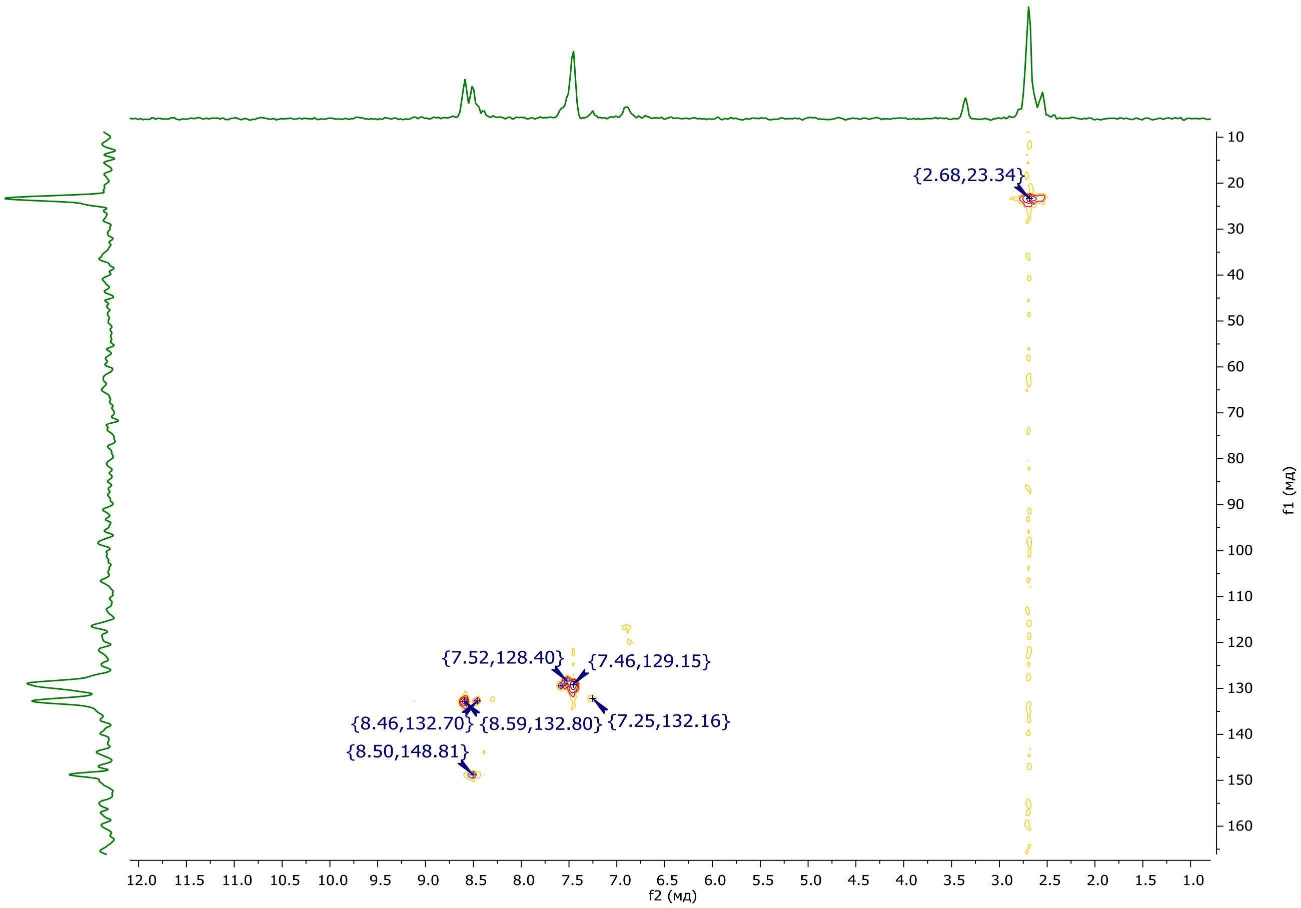

Figure S2.3: HMQC spectra of **4**

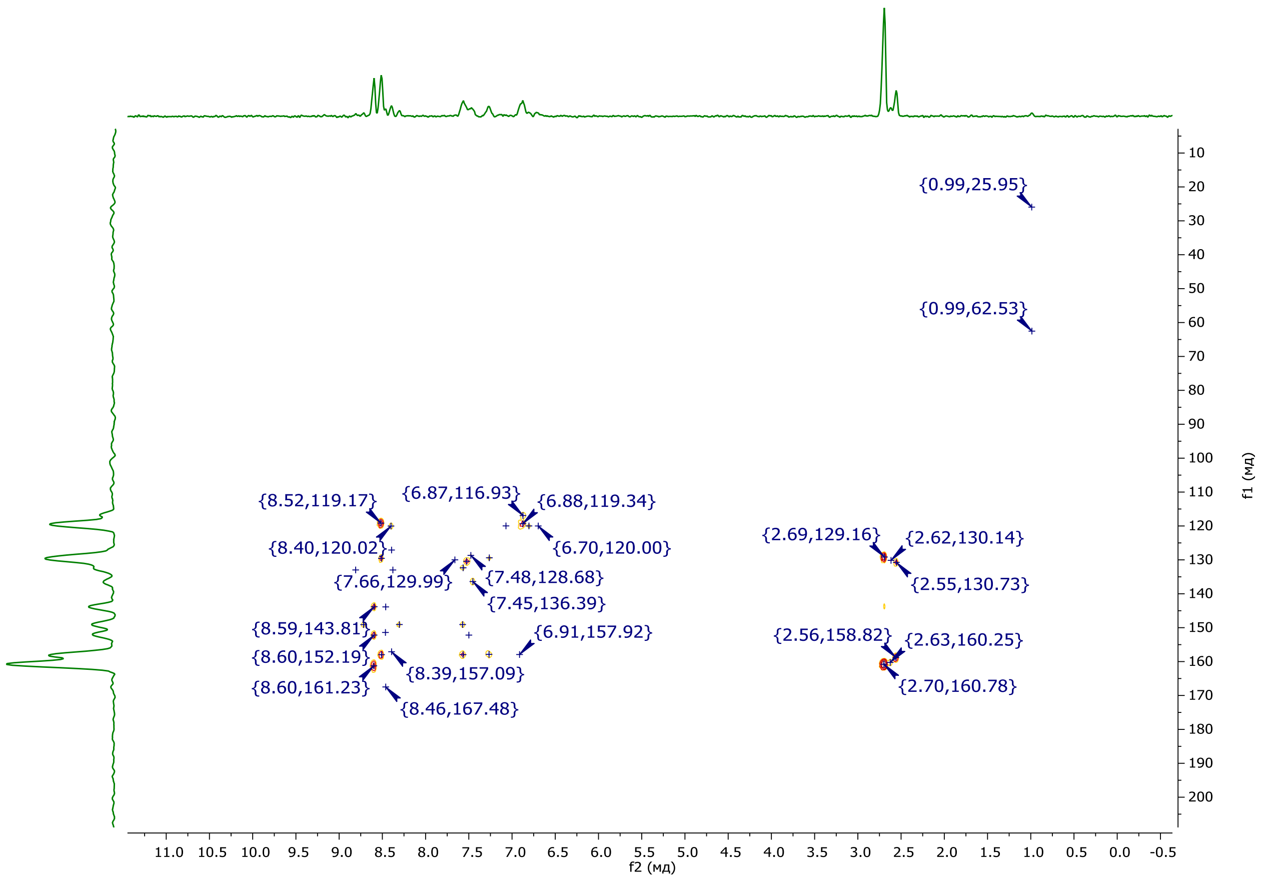

Figure S2.4: HMBC spectra of **4**

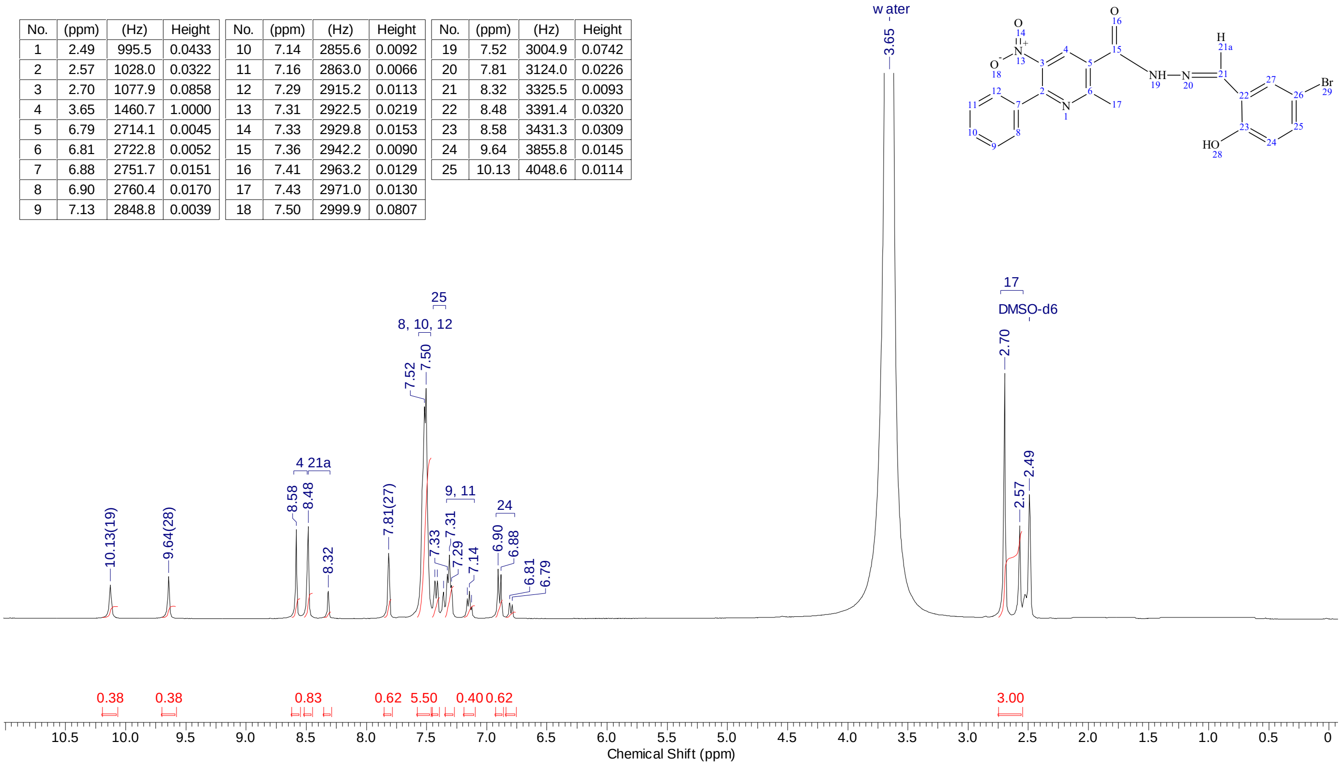

No.	(ppm)	(Hz)	Height
1	2.49	995.5	0.0433
2	2.57	1028.0	0.0322
3	2.70	1077.9	0.0858
4	3.65	1460.7	1.0000
5	6.79	2714.1	0.0045
6	6.81	2722.8	0.0052
7	6.88	2751.7	0.0151
8	6.90	2760.4	0.0170
9	7.13	2848.8	0.0039
10	7.14	2855.6	0.0092
11	7.16	2863.0	0.0066
12	7.29	2915.2	0.0113
13	7.31	2922.5	0.0219
14	7.33	2929.8	0.0153
15	7.36	2942.2	0.0090
16	7.41	2963.2	0.0129
17	7.43	2971.0	0.0130
18	7.50	2999.9	0.0807
19	7.52	3004.9	0.0742
20	7.81	3124.0	0.0226
21	8.32	3325.5	0.0093
22	8.48	3391.4	0.0320
23	8.58	3431.3	0.0309
24	9.64	3855.8	0.0145
25	10.13	4048.6	0.0114

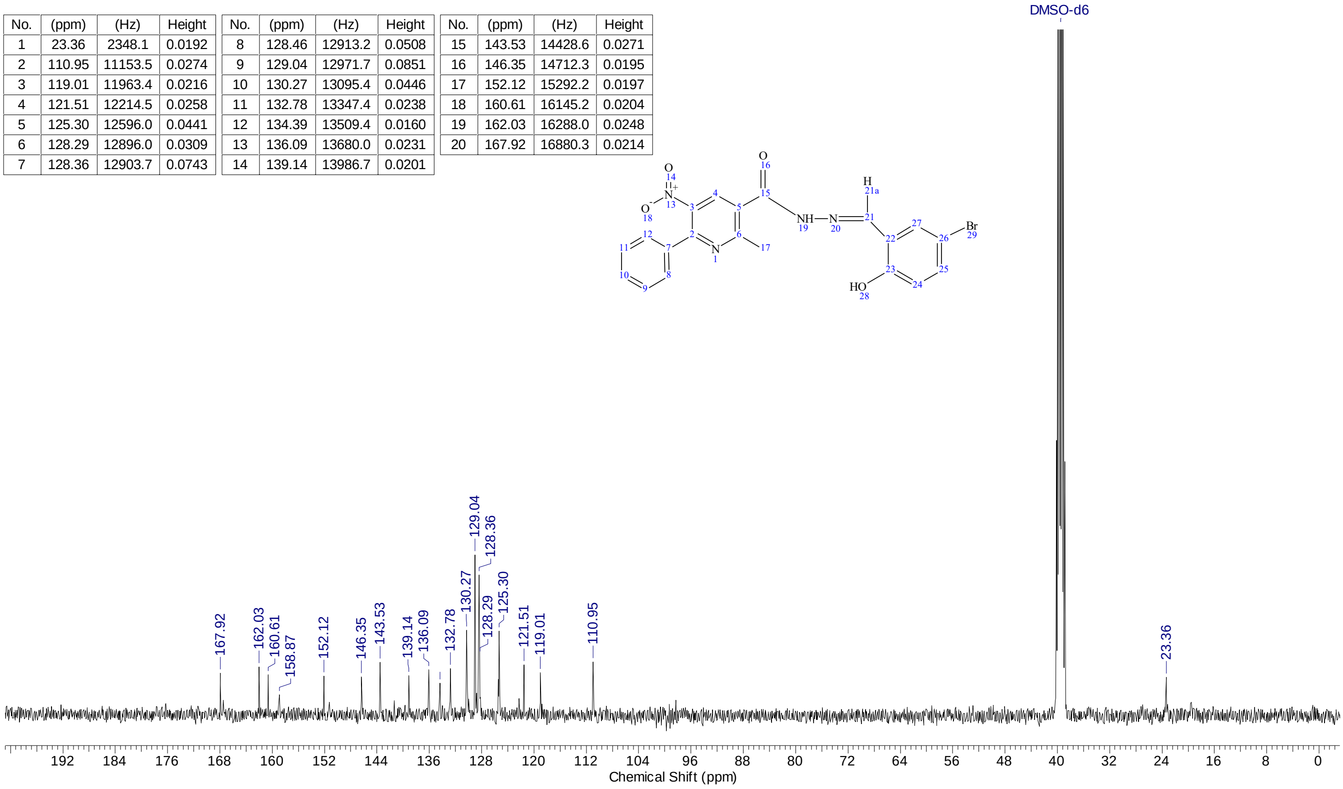

No.	(ppm)	(Hz)	Height
1	23.36	2348.1	0.0192
2	110.95	11153.5	0.0274
3	119.01	11963.4	0.0216
4	121.51	12214.5	0.0258
5	125.30	12596.0	0.0441
6	128.29	12896.0	0.0309
7	128.36	12903.7	0.0743
8	128.46	12913.2	0.0508
9	129.04	12971.7	0.0851
10	130.27	13095.4	0.0446
11	132.78	13347.4	0.0238
12	134.39	13509.4	0.0160
13	136.09	13680.0	0.0231
14	139.14	13986.7	0.0201
15	143.53	14428.6	0.0271
16	146.35	14712.3	0.0195
17	152.12	15292.2	0.0197
18	160.61	16145.2	0.0204
19	162.03	16288.0	0.0248
20	167.92	16880.3	0.0214

Figure S3.1: ^{1}H NMR (400 MHz, DMSO-d_6) and ^{13}C NMR (100 MHz, DMSO-d_6) spectra of **5**

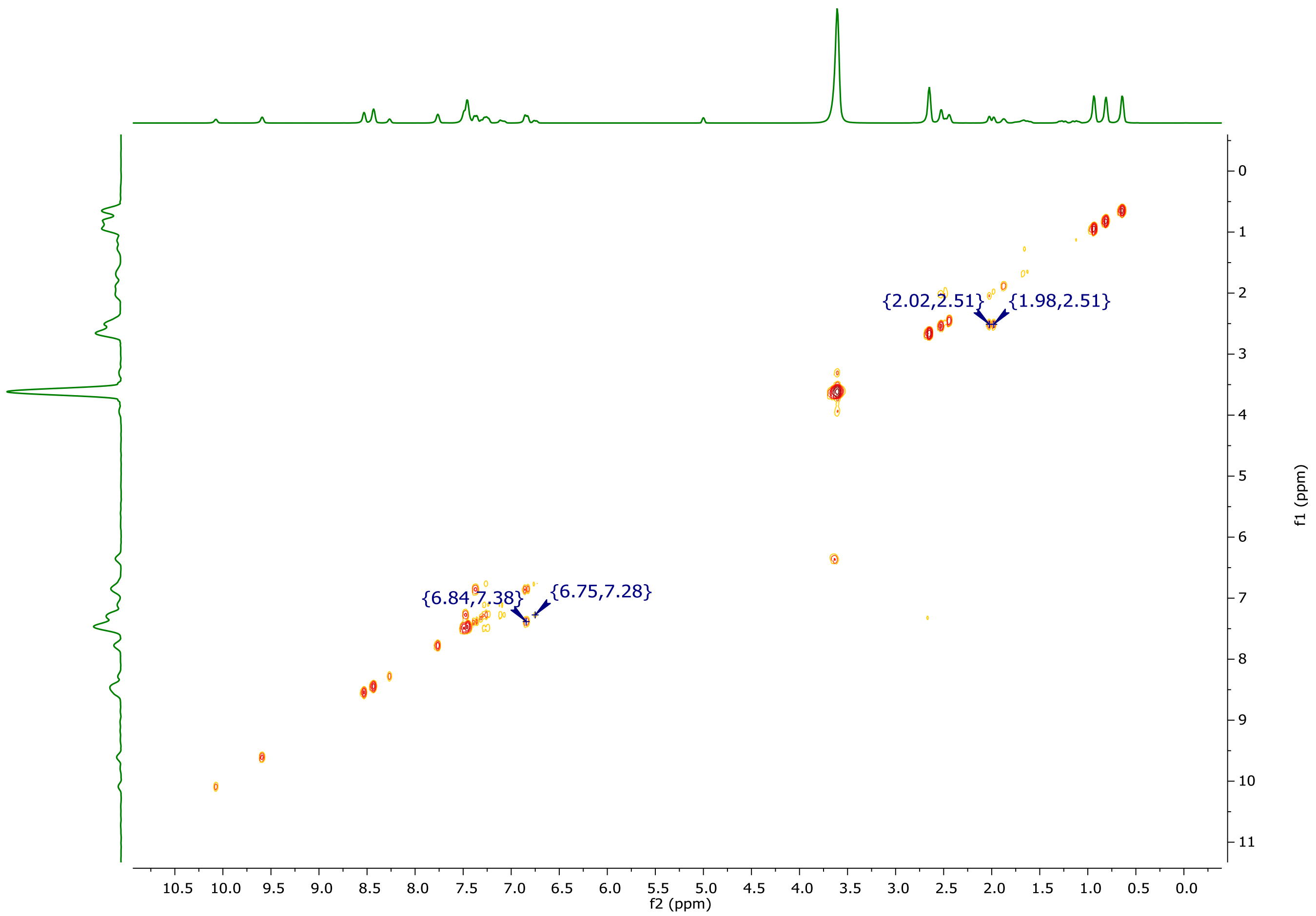

Figure S3.2: ^1H-^1H COSY spectra of **5**

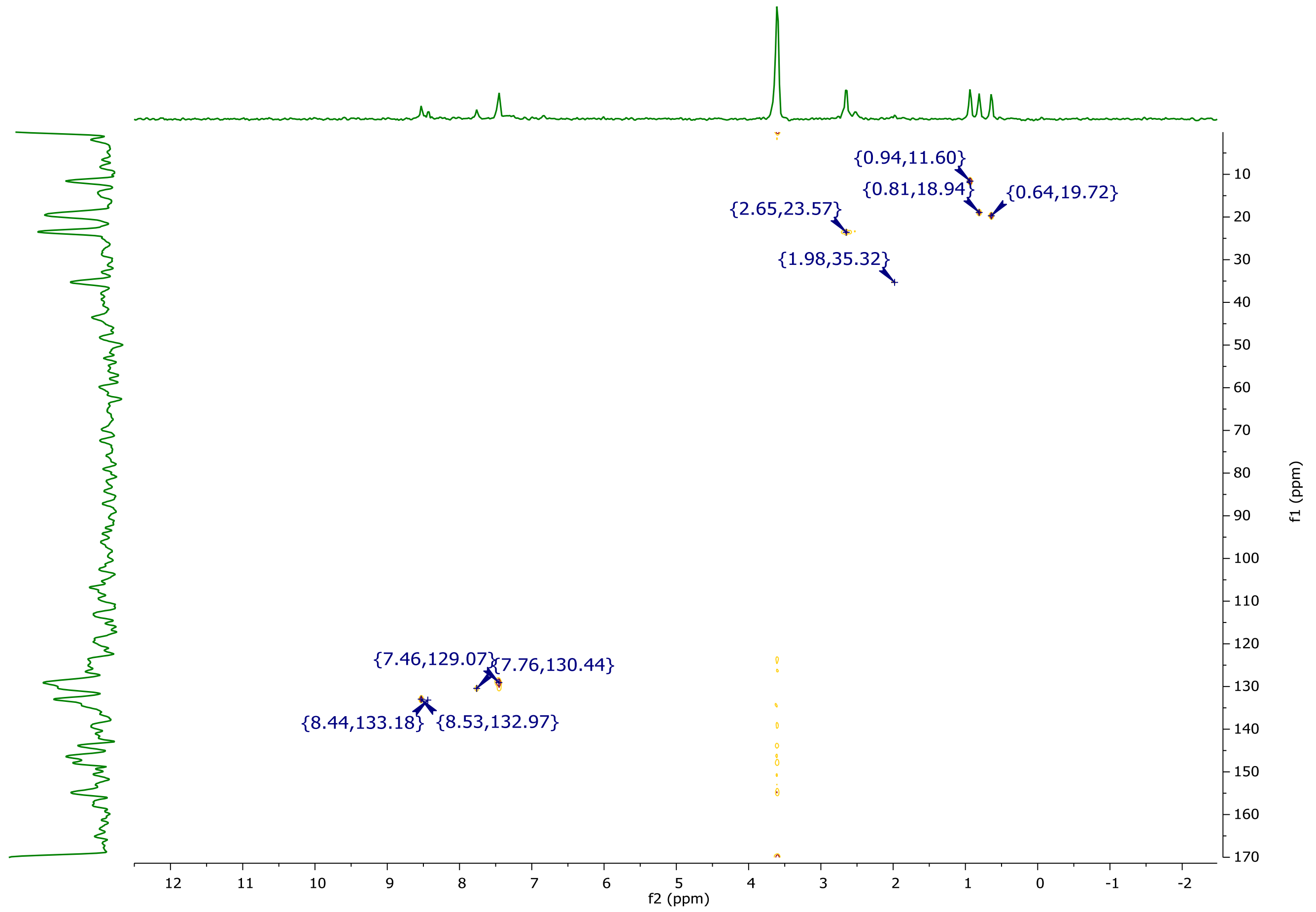

Figure S3.3: HMQC spectra of **5**

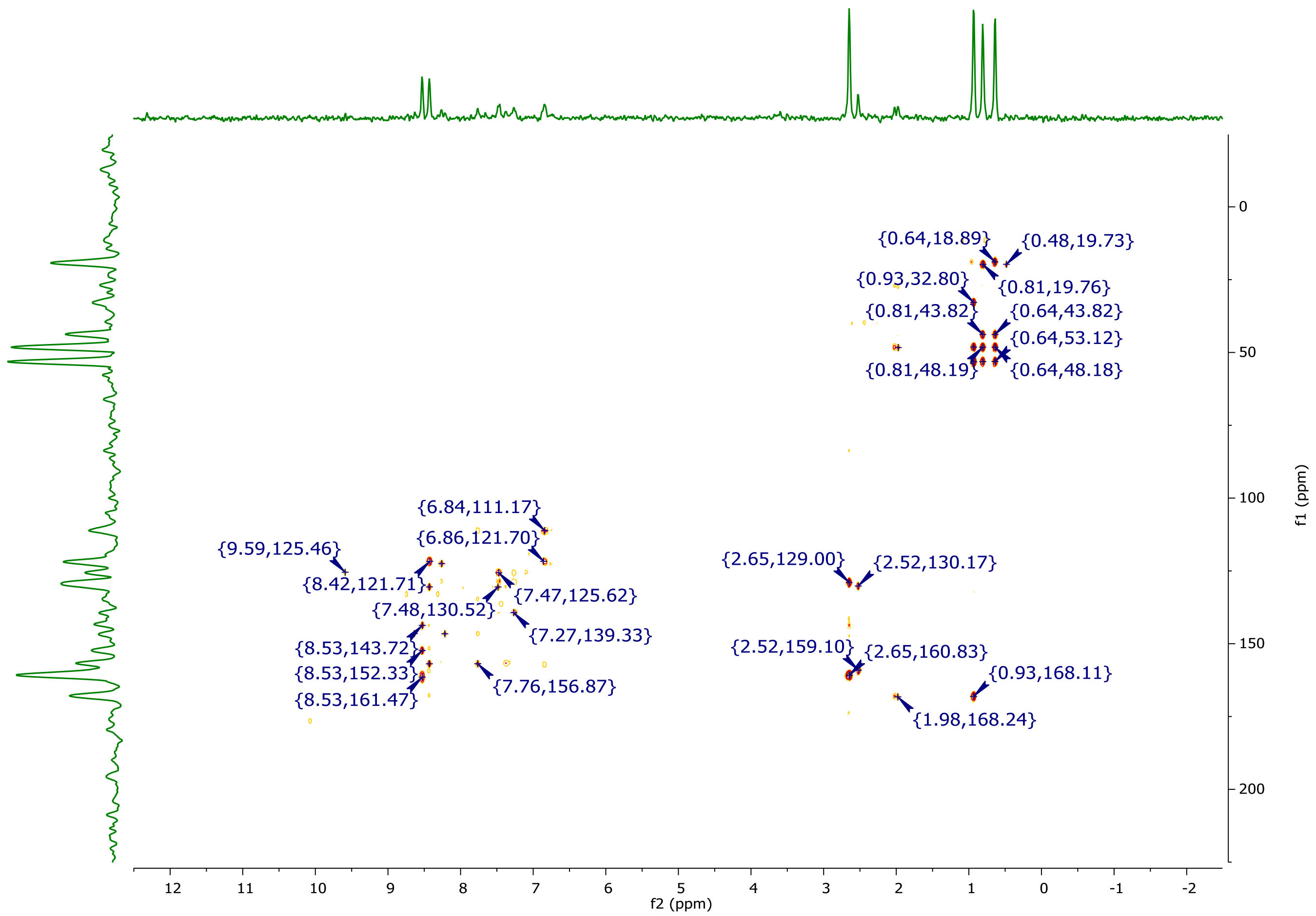

Figure S3.4: HMBC spectra of **5**

No.	(ppm)	(Hz)	Height
1	2.49	995.5	0.0342
2	2.58	1032.5	0.0385
3	2.68	1072.4	0.0818
4	3.66	1461.6	1.0000
5	3.81	1523.5	0.1211
6	6.75	2699.9	0.0101
7	6.85	2738.4	0.0228
8	6.95	2778.7	0.0141
9	7.10	2837.3	0.0213

No.	(ppm)	(Hz)	Height
10	7.38	2949.1	0.0302
11	7.50	2998.5	0.1396
12	7.99	3194.5	0.0113
13	8.17	3265.5	0.0261
14	8.48	3388.7	0.0140
15	8.53	3408.8	0.0283
16	9.81	3921.3	0.0006
17	11.98	4789.5	0.0022

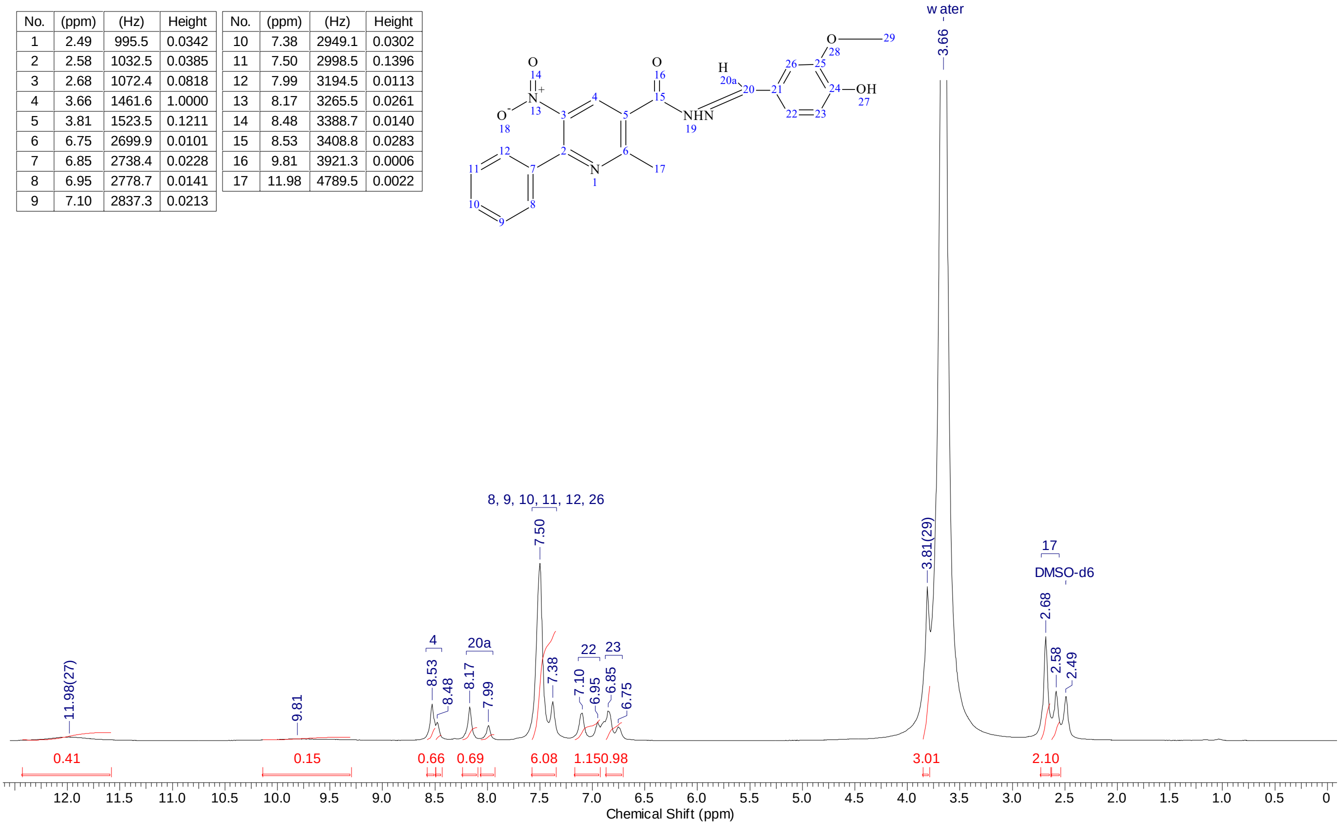

No.	(ppm)	(Hz)	Height
1	23.22	2334.6	0.0522
2	55.28	5557.0	0.0182
3	55.83	5612.6	0.0396
4	109.02	10958.9	0.0174
5	109.28	10985.8	0.0431
6	115.72	11632.7	0.0475
7	122.96	12360.2	0.0374

No.	(ppm)	(Hz)	Height
8	125.47	12613.2	0.0488
9	128.33	12900.8	0.1279
10	129.01	12968.8	0.1406
11	129.54	13021.6	0.0262
12	130.08	13076.2	0.0177
13	130.20	13088.6	0.0437
14	132.54	13323.5	0.0446

No.	(ppm)	(Hz)	Height
15	133.06	13376.2	0.0143
16	136.15	13686.7	0.0259
17	136.28	13699.2	0.0159
18	143.50	14425.7	0.0159
19	143.58	14433.4	0.0316
20	148.17	14894.4	0.0171
21	148.38	14916.4	0.0360

No.	(ppm)	(Hz)	Height
22	149.62	15041.0	0.0382
23	149.74	15052.5	0.0412
24	151.88	15268.2	0.0284
25	160.34	16118.4	0.0274
26	161.91	16276.5	0.0278
27	167.23	16811.3	0.0143
28	167.28	16816.1	0.0184

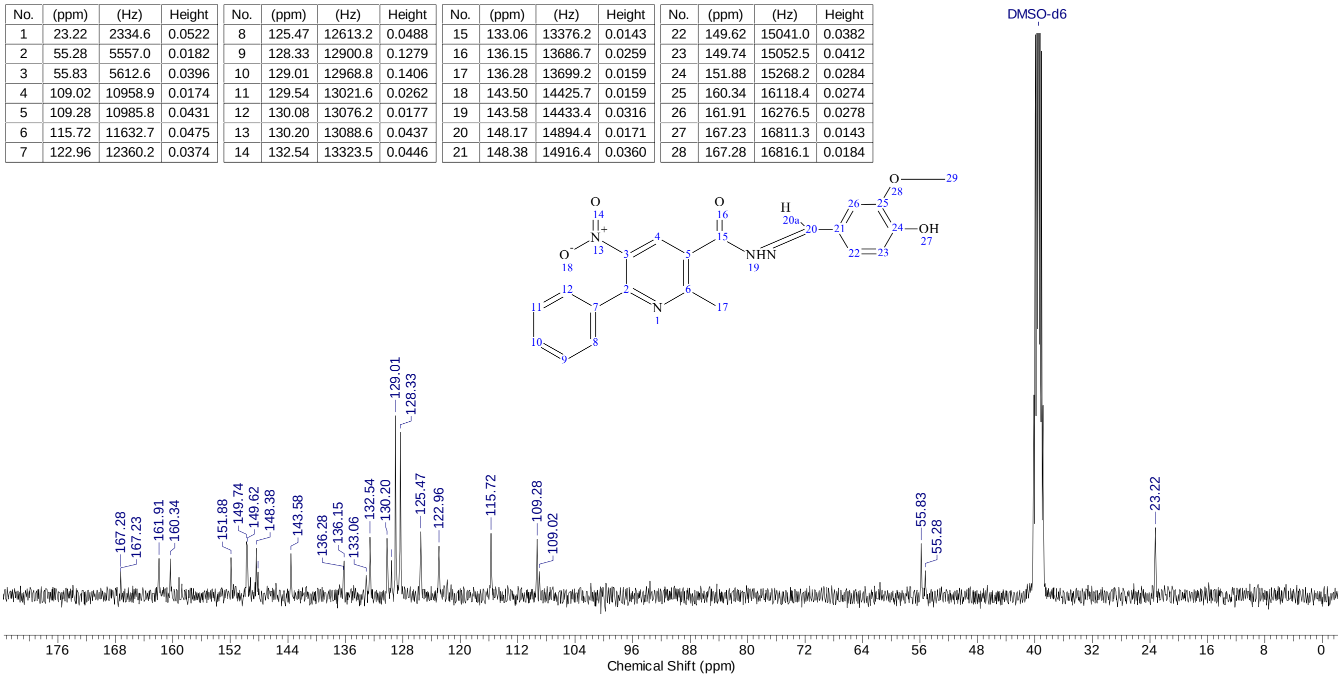

Figure S4.1: ^{1}H NMR (400 MHz, DMSO-d_6) and ^{13}C NMR (100 MHz, DMSO-d_6) spectra of **6**

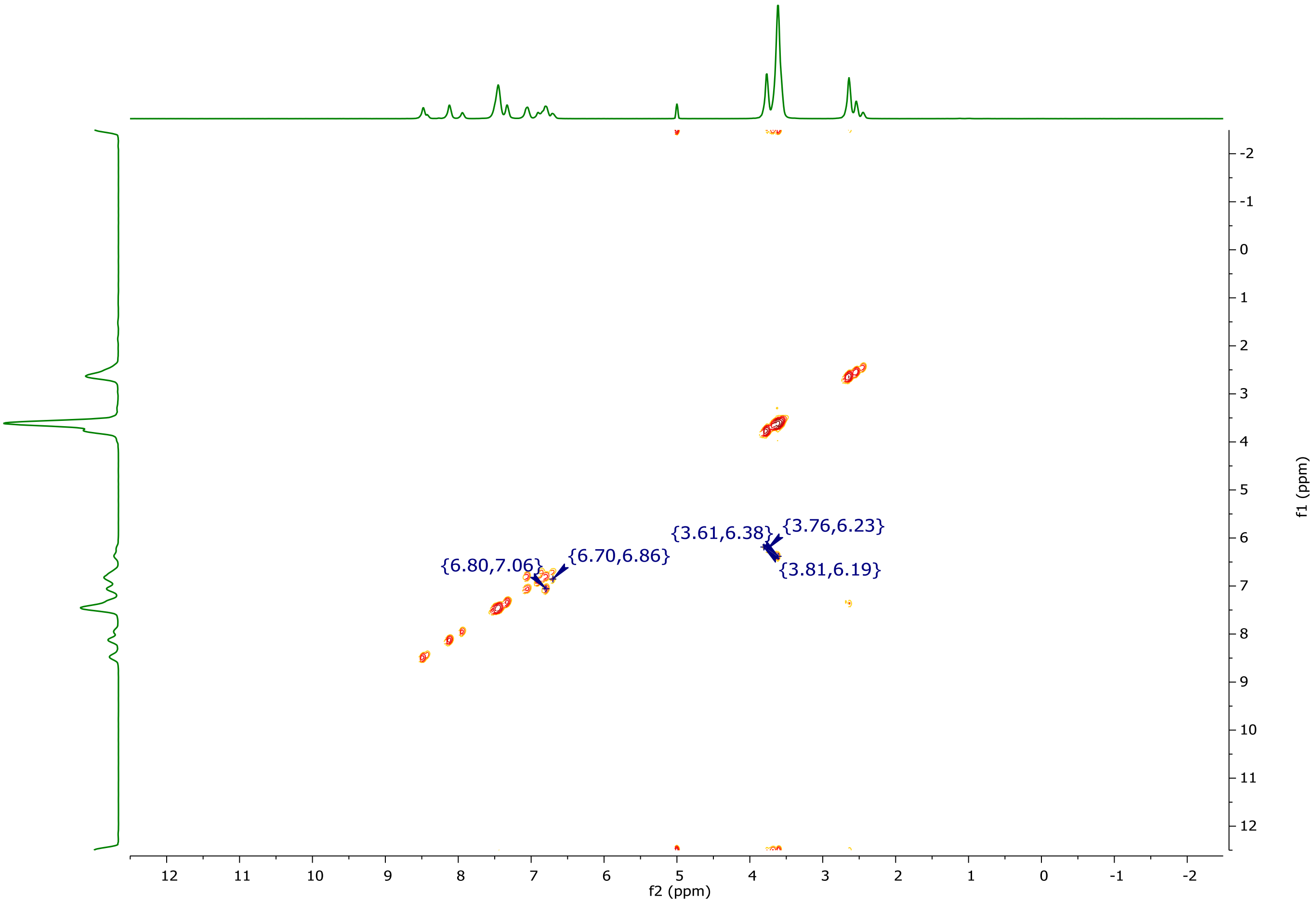

Figure S4.2: ^{1}H-^{1}H COSY spectra of **6**

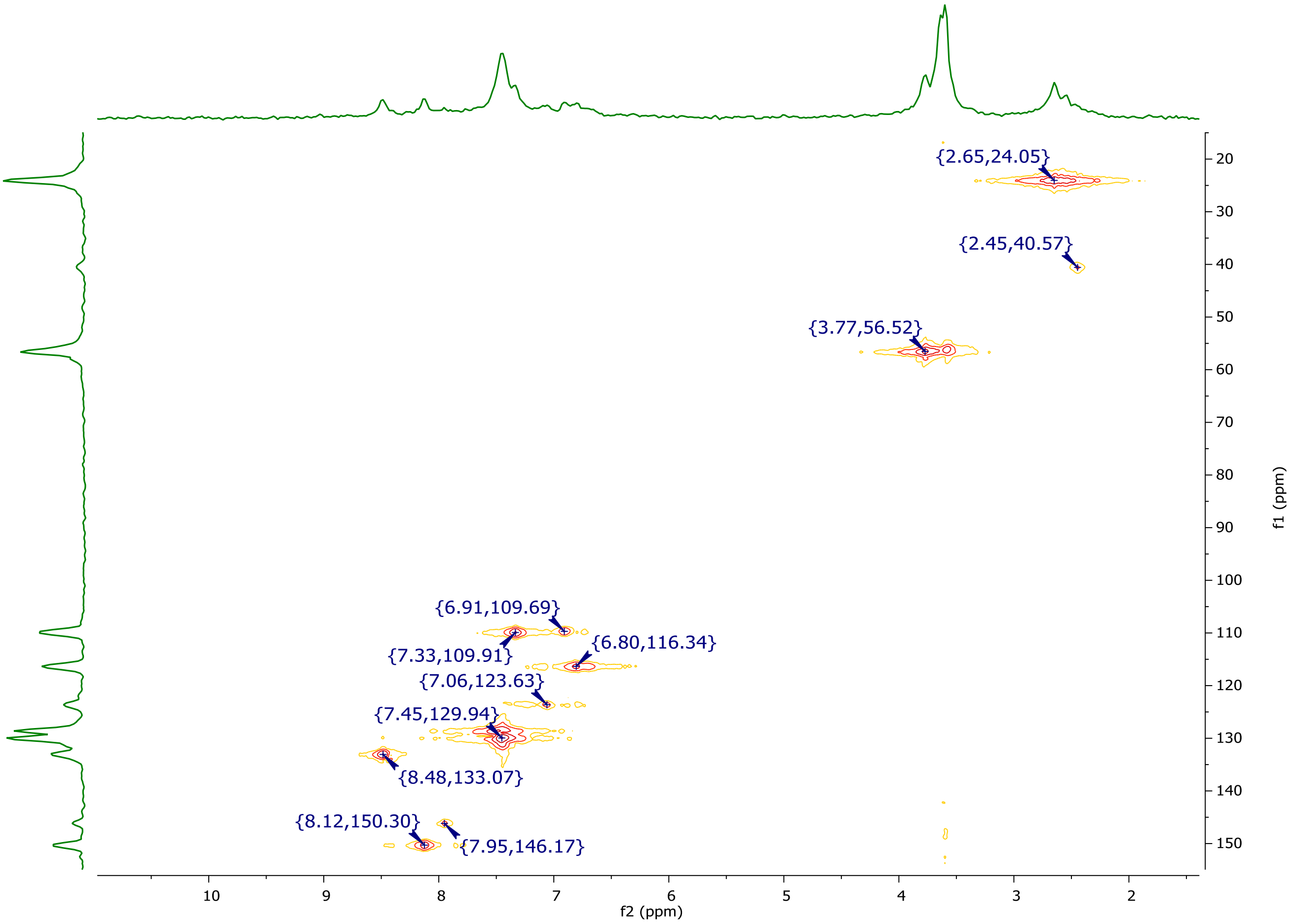

Figure S4.3: HMQC spectra of **6**

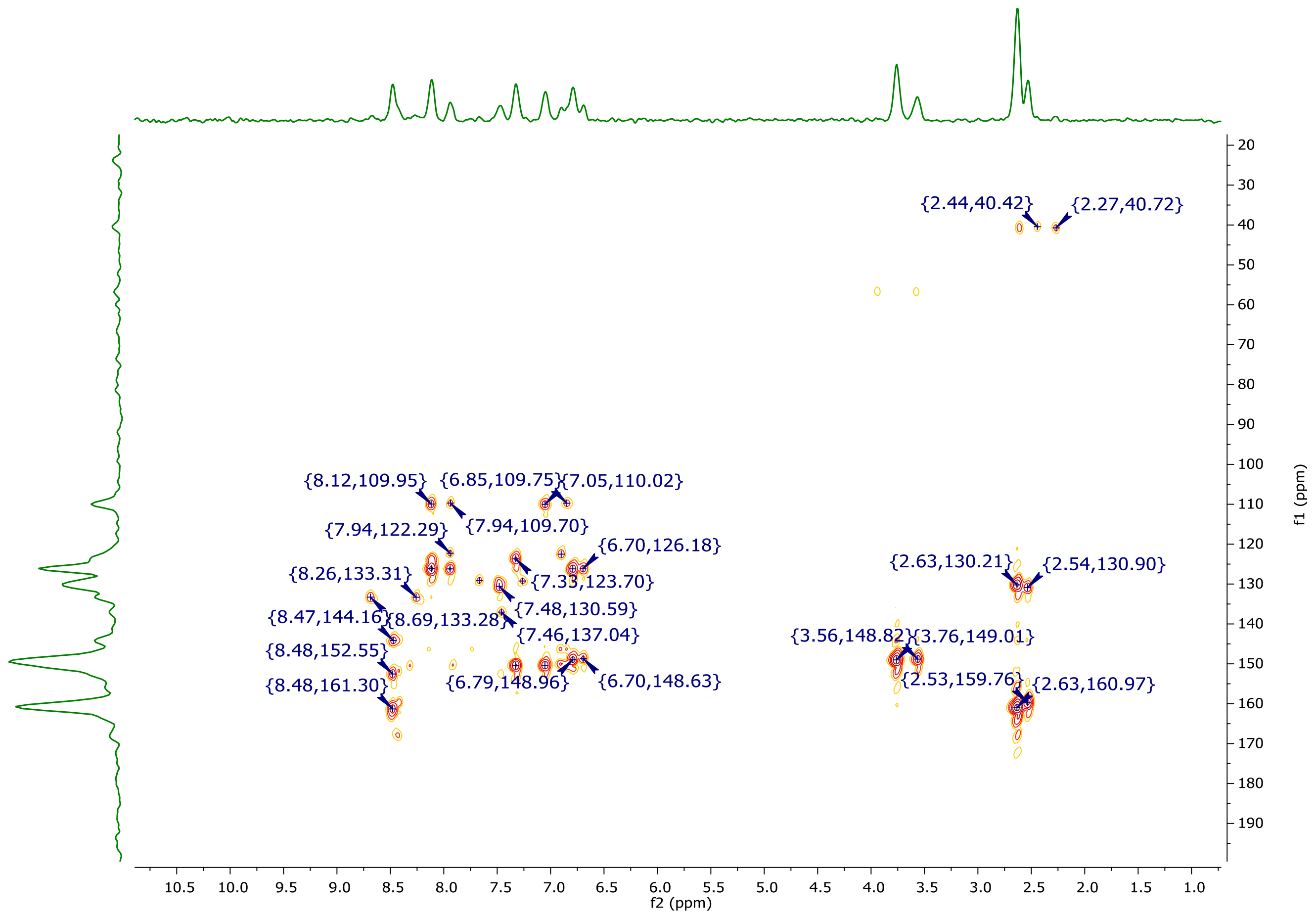

Figure S4.4: HMBC spectra of **6**

No.	(ppm)	(Hz)	Height	No.	(ppm)	(Hz)	Height	No.	(ppm)	(Hz)	Height	No.	(ppm)	(Hz)	Height
1	2.58	1030.3	0.0368	8	7.52	3007.2	0.0859	15	7.90	3157.4	0.0207	22	8.58	3429.5	0.0395
2	2.59	1035.3	0.0549	9	7.53	3010.4	0.0844	16	7.92	3164.8	0.0178	23	8.86	3542.1	0.0122
3	2.69	1074.2	0.0746	10	7.64	3052.6	0.0155	17	8.09	3233.5	0.0200	24	8.87	3546.7	0.0184
4	2.70	1078.8	0.1112	11	7.65	3058.5	0.0190	18	8.11	3240.3	0.0177	25	9.06	3622.7	0.0247
5	3.82	1525.8	1.0000	12	7.67	3065.4	0.0095	19	8.47	3386.4	0.0129	26	9.07	3627.3	0.0370
6	7.49	2995.8	0.1027	13	7.83	3129.5	0.0104	20	8.48	3391.0	0.0201	27	12.32	4925.6	0.0303
7	7.51	3000.8	0.0993	14	7.84	3135.5	0.0082	21	8.57	3424.9	0.0265	28	12.36	4943.0	0.0145

No.	Atom	Exp. Shift (ppm)	No.	Atom	Exp. Shift (ppm)	No.	Atom	Exp. Shift (ppm)
1	17	2.59	8	11	[7.45 .. 7.56]	15	4	8.48
2	17	2.70	9	12	[7.45 .. 7.56]	16	4	8.58
3	26	[7.45 .. 7.56]	10	27	[7.60 .. 7.69]	17	21a	8.87
4	25	[7.45 .. 7.56]	11	26	[7.60 .. 7.69]	18	21a	9.07
5	8	[7.45 .. 7.56]	12	24	[7.81 .. 7.85]	19	19	12.32
6	9	[7.45 .. 7.56]	13	27	[7.89 .. 7.92]	20	19	12.36
7	10	[7.45 .. 7.56]	14	24	[8.07 .. 8.11]			

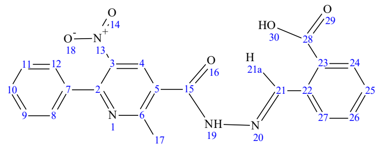

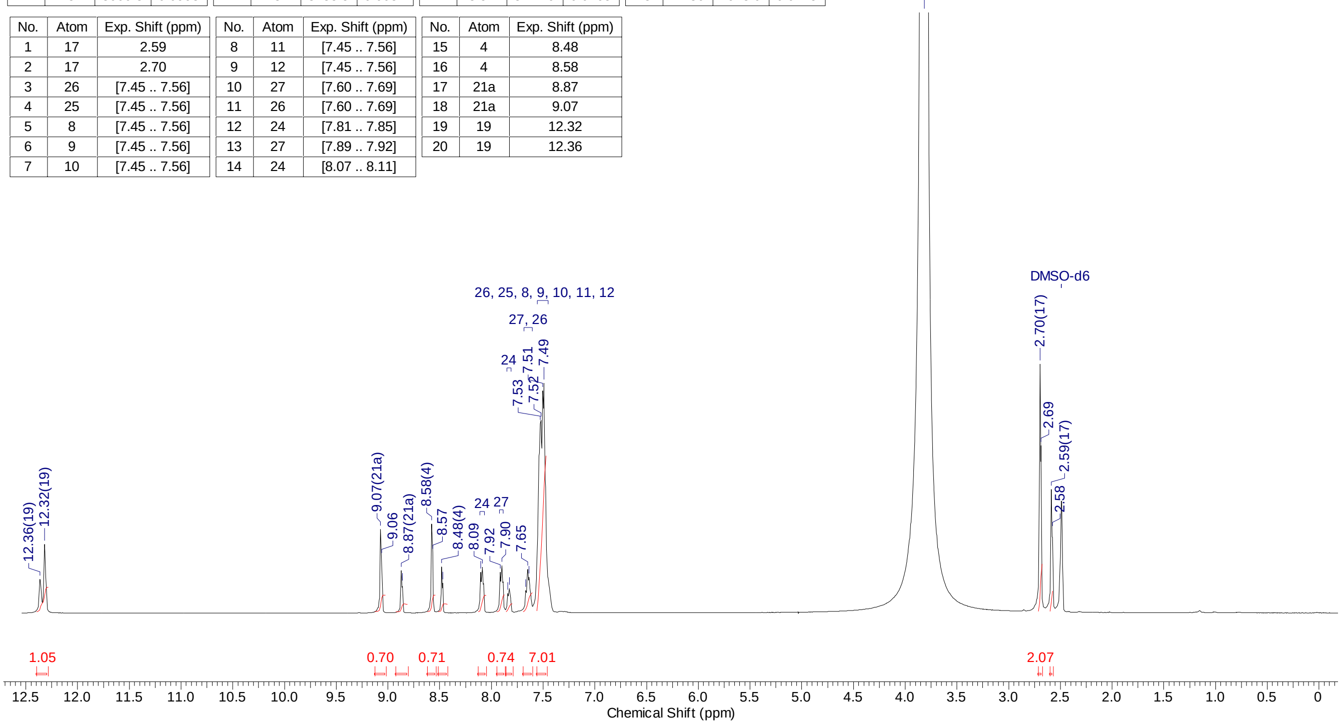

No.	(ppm)	(Hz)	Height	No.	(ppm)	(Hz)	Height	No.	(ppm)	(Hz)	Height	No.	(ppm)	(Hz)	Height
1	23.23	2335.6	0.0200	10	130.18	13086.7	0.0181	19	132.79	13348.4	0.0336	28	151.33	15212.6	0.0104
2	23.34	2346.1	0.0384	11	130.29	13097.3	0.0254	20	132.90	13359.9	0.0152	29	151.99	15278.7	0.0241
3	126.60	12726.3	0.0133	12	130.46	13114.5	0.0265	21	134.19	13489.3	0.0150	30	160.52	16136.6	0.0246
4	127.10	12777.1	0.0263	13	130.68	13136.6	0.0151	22	134.36	13506.5	0.0251	31	162.35	16320.6	0.0235
5	128.37	12904.6	0.1004	14	130.79	13148.1	0.0288	23	136.13	13684.8	0.0264	32	168.31	16919.6	0.0172
6	128.43	12910.4	0.0565	15	130.98	13167.2	0.0141	24	136.23	13694.4	0.0116	33	168.42	16930.2	0.0327
7	128.99	12966.9	0.0477	16	131.09	13177.8	0.0211	25	143.56	14431.5	0.0284				
8	129.07	12974.6	0.0934	17	132.43	13312.9	0.0167	26	143.67	14442.0	0.0142				
9	129.20	12988.0	0.0301	18	132.52	13321.5	0.0314	27	148.20	14898.2	0.0322				

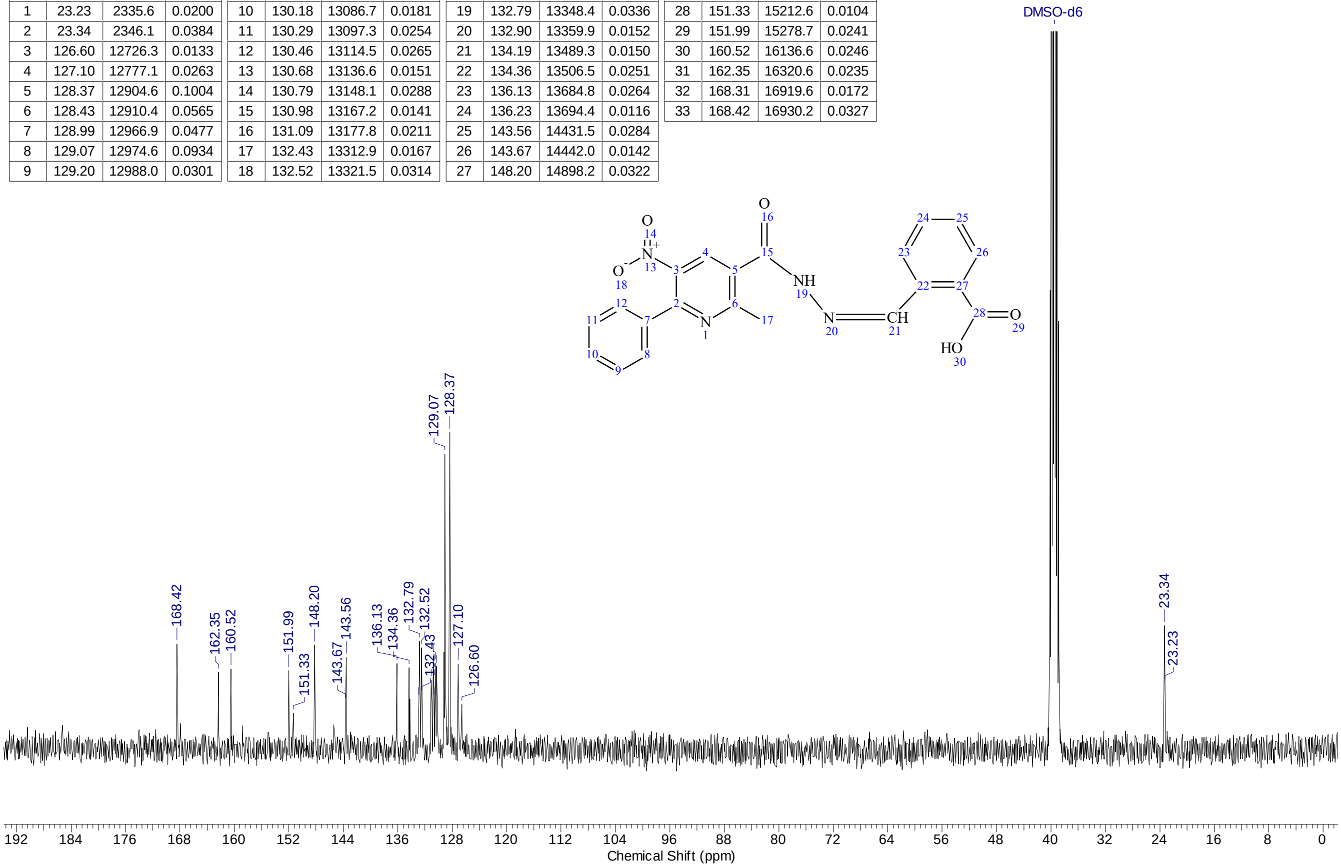

Figure S5.1: ^{1}H NMR (400 MHz, DMSO-d_6) and ^{13}C NMR (100 MHz, DMSO-d_6) spectra of **7**

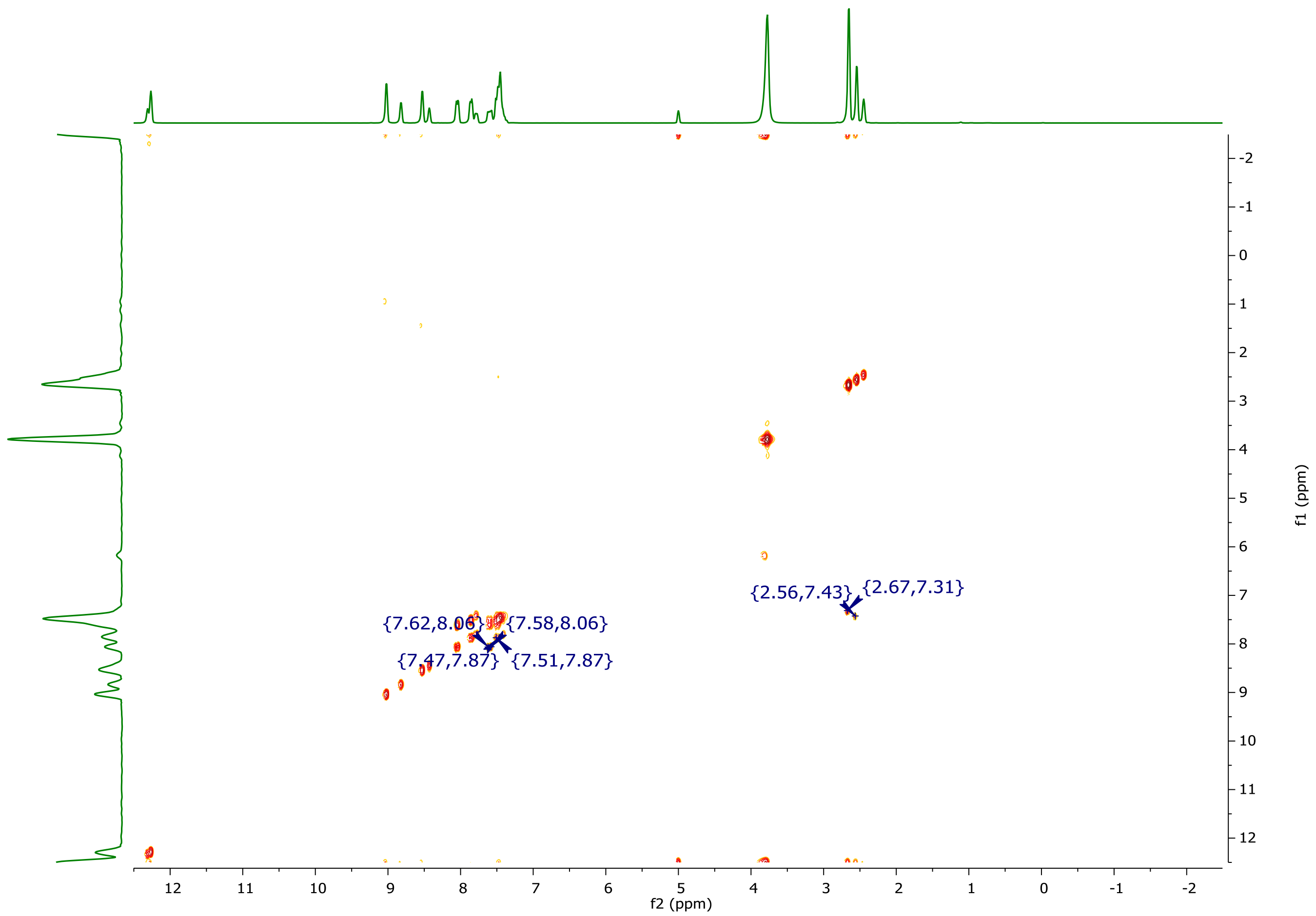

Figure S5.2: ^{1}H-^{1}H COSY spectra of **7**

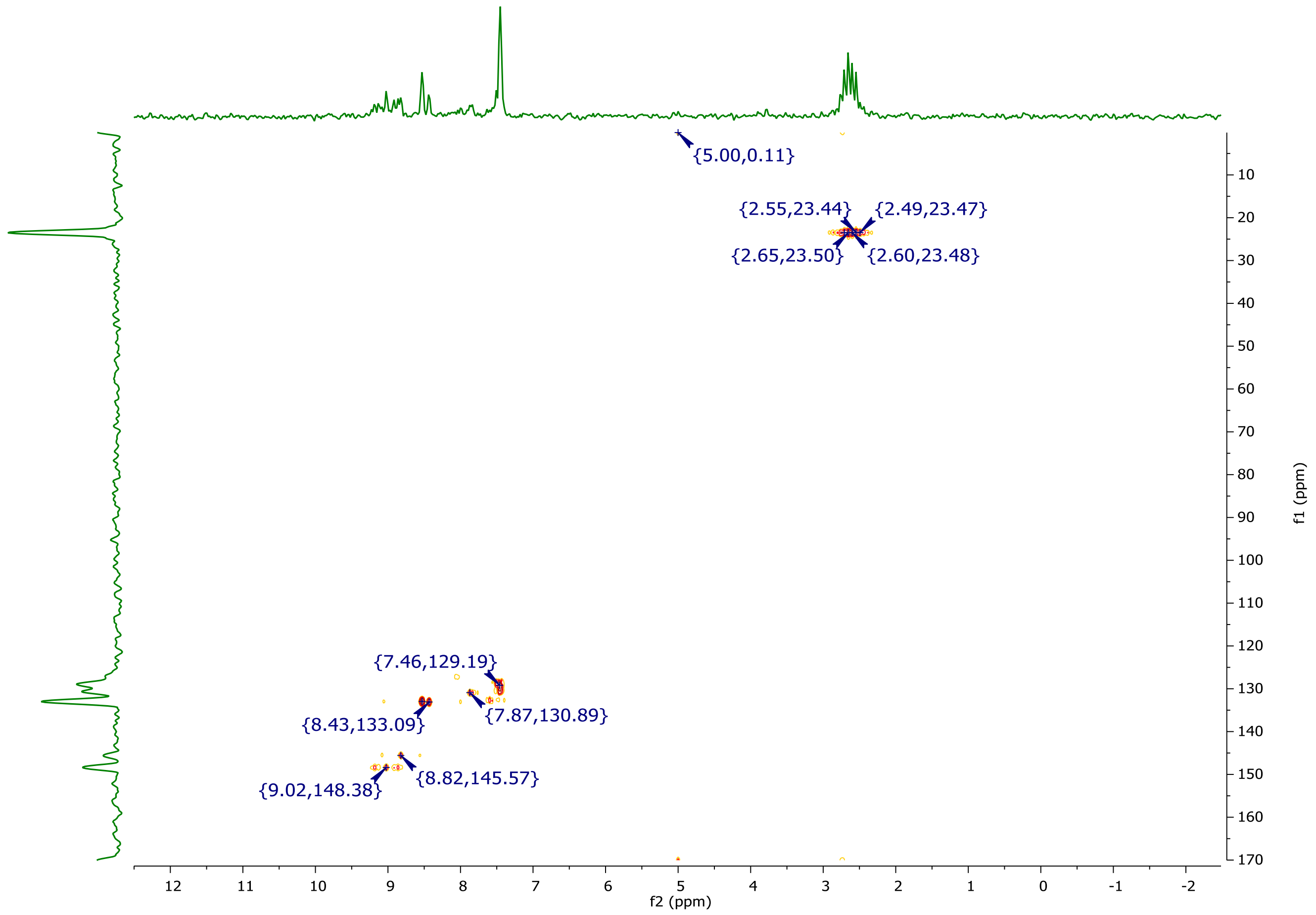

Figure S5.3: HMQC spectra of **7**

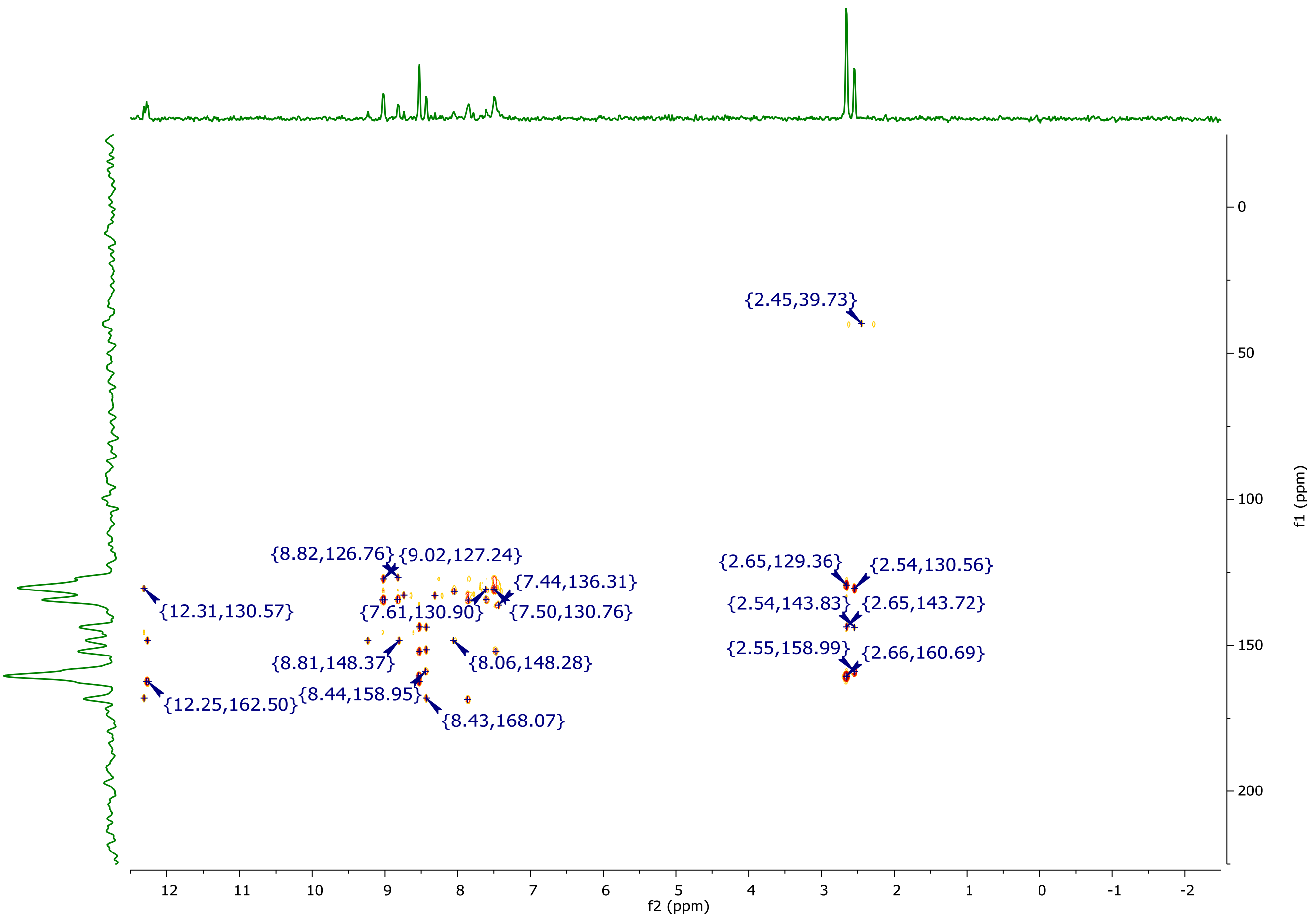

Figure S5.4: HMBC spectra of **7**

No.	(ppm)	(Hz)	Height
1	2.49	995.5	0.2972
2	2.59	1035.8	0.1507
3	2.72	1088.4	0.2117
4	7.19	2873.0	0.0570
5	7.31	2920.7	0.1489
6	7.52	3006.8	0.5127

No.	(ppm)	(Hz)	Height
7	7.96	3180.8	0.0673
8	8.34	3334.2	0.0456
9	8.53	3408.8	0.1090
10	8.65	3456.5	0.0742
11	12.23	4889.8	0.0602

No.	Atom	Exp. Shift (ppm)
1	17	[2.55 .. 2.80]
2	21a	[8.27 .. 8.38]
3	4	[8.45 .. 8.55]
4	21a	[8.59 .. 8.69]
5	19	12.23

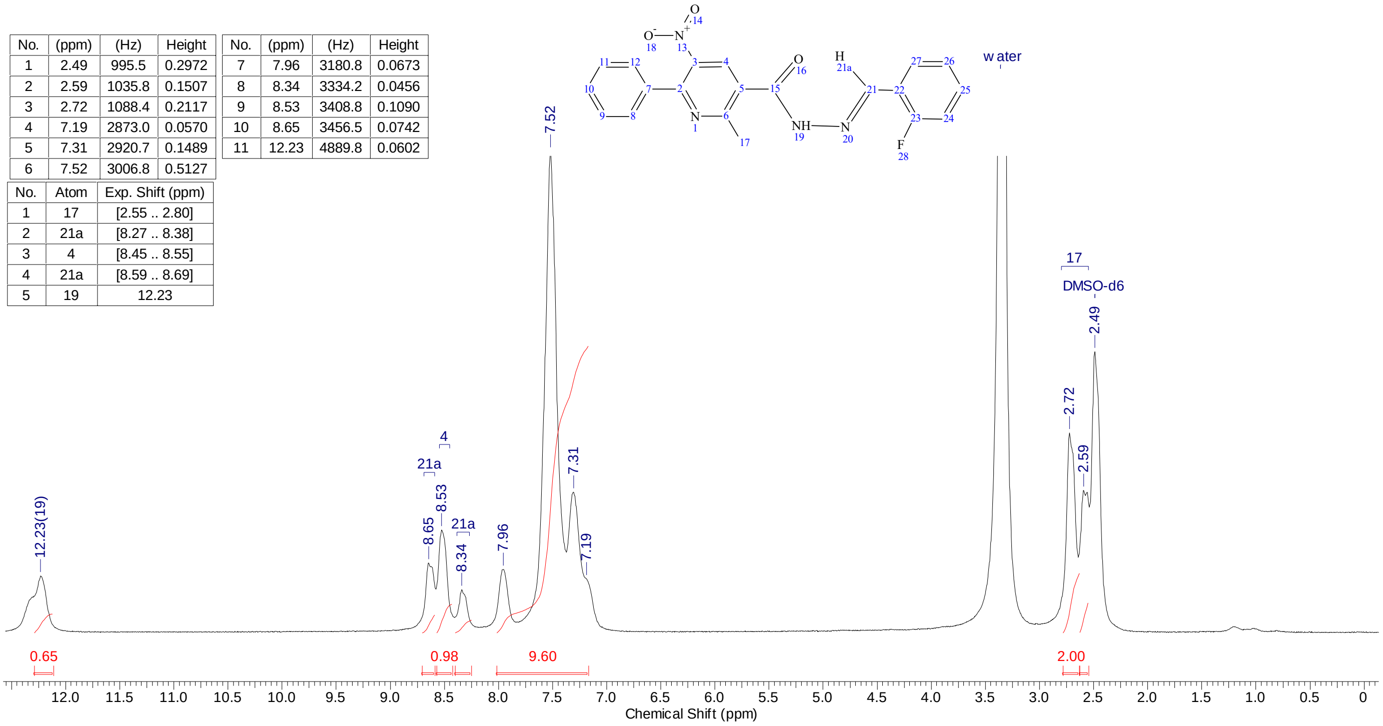

No.	(ppm)	(Hz)	Height
1	23.07	2319.3	0.0243
2	116.03	11664.4	0.0153
3	116.25	11686.4	0.0135
4	121.29	12192.5	0.0104
5	121.50	12213.6	0.0126
6	125.10	12575.9	0.0230
7	128.11	12878.7	0.0484
8	128.74	12942.0	0.0453

No.	(ppm)	(Hz)	Height
9	128.91	12958.3	0.0164
10	129.89	13057.0	0.0175
11	129.98	13066.6	0.0182
12	132.48	13317.7	0.0273
13	132.70	13339.8	0.0150
14	132.79	13348.4	0.0165
15	135.84	13655.1	0.0124
16	138.45	13917.7	0.0103

No.	(ppm)	(Hz)	Height
17	141.53	14227.3	0.0158
18	143.36	14411.3	0.0134
19	151.70	15250.0	0.0095
20	158.54	15937.2	0.0061
21	159.69	16053.2	0.0063
22	160.20	16104.0	0.0141
23	161.64	16248.7	0.0115
24	167.45	16833.4	0.0081

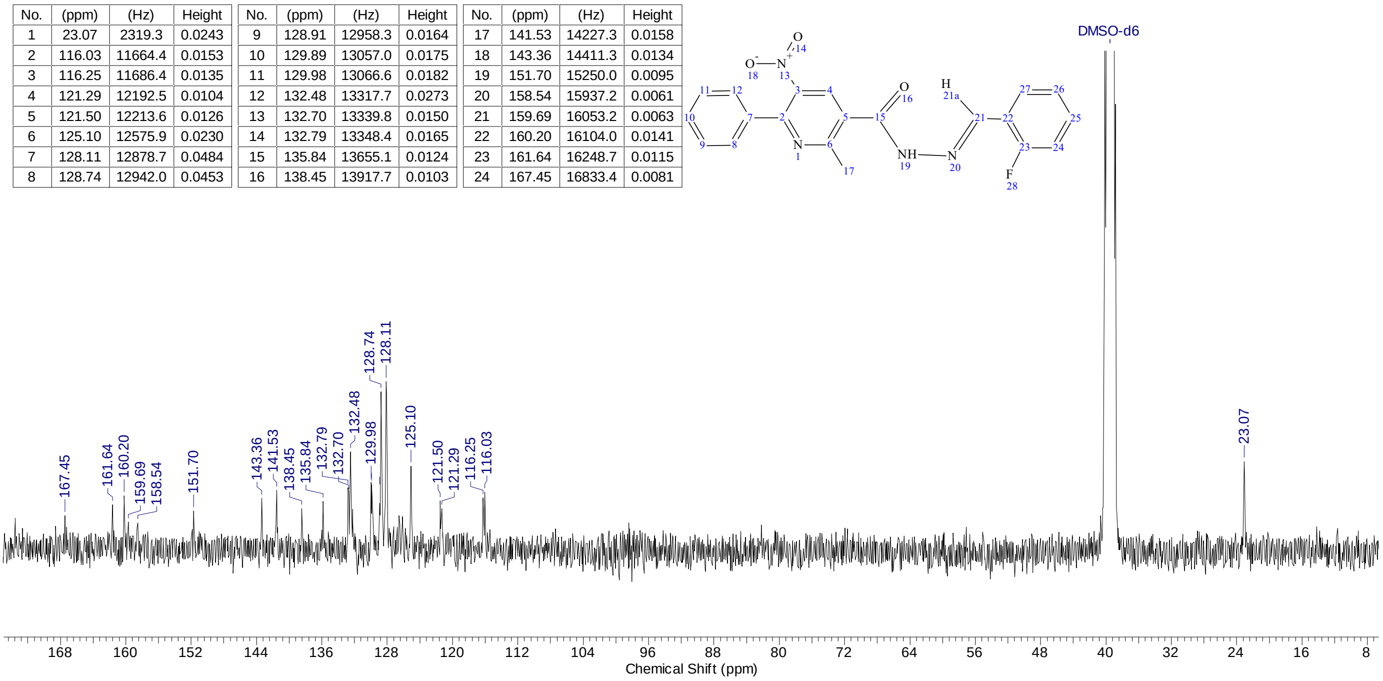

Figure S6: ^{1}H NMR (400 MHz, DMSO-d_6) and ^{13}C NMR (100 MHz, DMSO-d_6) spectra of **8**

No.	(ppm)	(Hz)	Height	No.	(ppm)	(Hz)	Height	No.	(ppm)	(Hz)	Height
1	2.49	995.5	0.0388	8	7.23	2888.6	0.0088	15	7.54	3015.9	0.0591
2	2.57	1029.3	0.0397	9	7.28	2910.1	0.0192	16	7.57	3026.9	0.0478
3	2.58	1033.0	0.0412	10	7.30	2916.5	0.0187	17	8.13	3250.4	0.0133
4	2.70	1077.9	0.0758	11	7.38	2951.3	0.0073	18	8.31	3320.9	0.0253
5	2.71	1081.6	0.0774	12	7.39	2955.9	0.0083	19	8.49	3393.7	0.0141
6	7.18	2870.3	0.0084	13	7.50	2999.0	0.1151	20	8.58	3431.7	0.0264
7	7.20	2878.5	0.0123	14	7.53	3011.3	0.0605	21	12.24	4893.5	0.0033

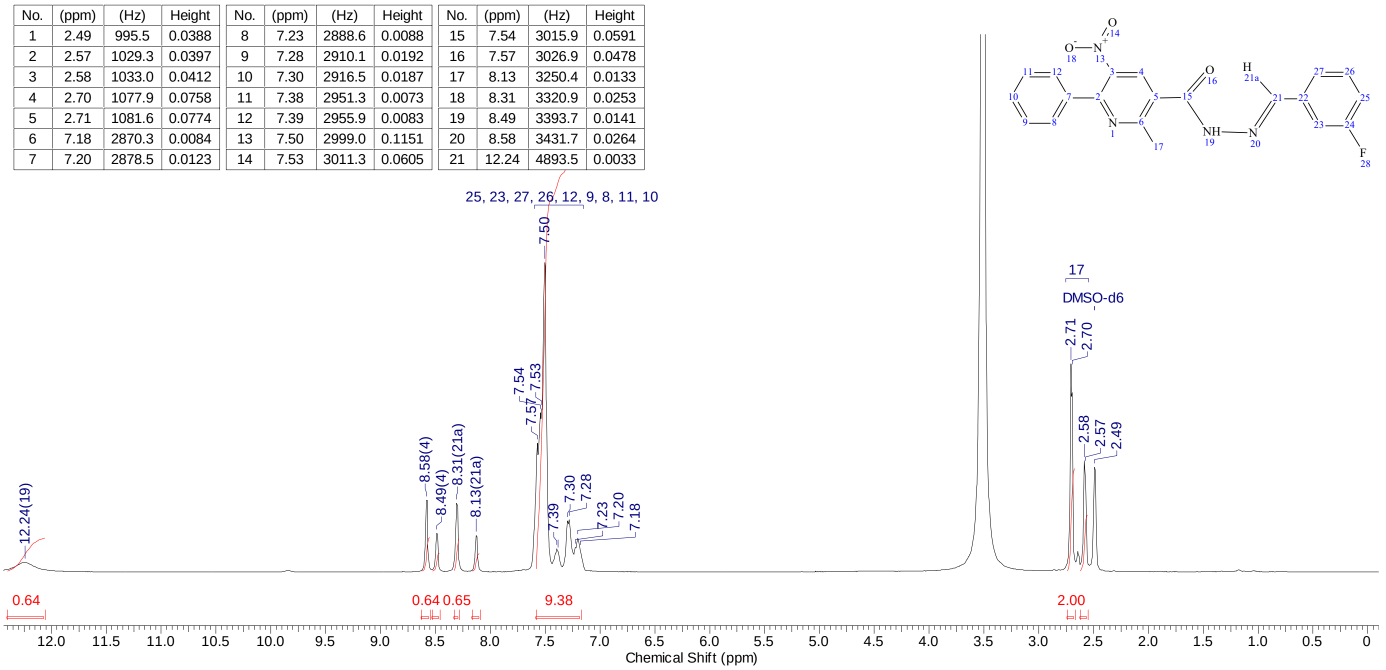

No.	(ppm)	(Hz)	Height	No.	(ppm)	(Hz)	Height	No.	(ppm)	(Hz)	Height	No.	(ppm)	(Hz)	Height
1	23.13	2325.1	0.0105	9	128.30	12897.0	0.0781	17	132.98	13367.6	0.0120	25	151.28	15207.8	0.0075
2	23.21	2333.7	0.0422	10	128.97	12965.0	0.0645	18	136.05	13676.2	0.0192	26	151.96	15275.9	0.0162
3	113.38	11397.9	0.0215	11	129.16	12984.2	0.0215	19	136.14	13685.8	0.0118	27	158.82	15965.0	0.0090
4	113.60	11420.0	0.0264	12	130.06	13074.3	0.0190	20	136.54	13726.0	0.0122	28	160.34	16118.4	0.0159
5	117.10	11771.7	0.0266	13	130.19	13087.7	0.0242	21	136.62	13733.7	0.0068	29	161.29	16214.2	0.0069
6	117.37	11798.5	0.0292	14	131.22	13191.2	0.0253	22	143.52	14427.6	0.0261	30	161.41	16225.7	0.0141
7	123.41	12406.2	0.0089	15	131.32	13200.8	0.0227	23	144.31	14507.2	0.0393	31	162.20	16305.3	0.0186
8	124.00	12464.7	0.0278	16	132.63	13333.1	0.0245	24	147.80	14858.0	0.0239	32	167.74	16862.1	0.0133

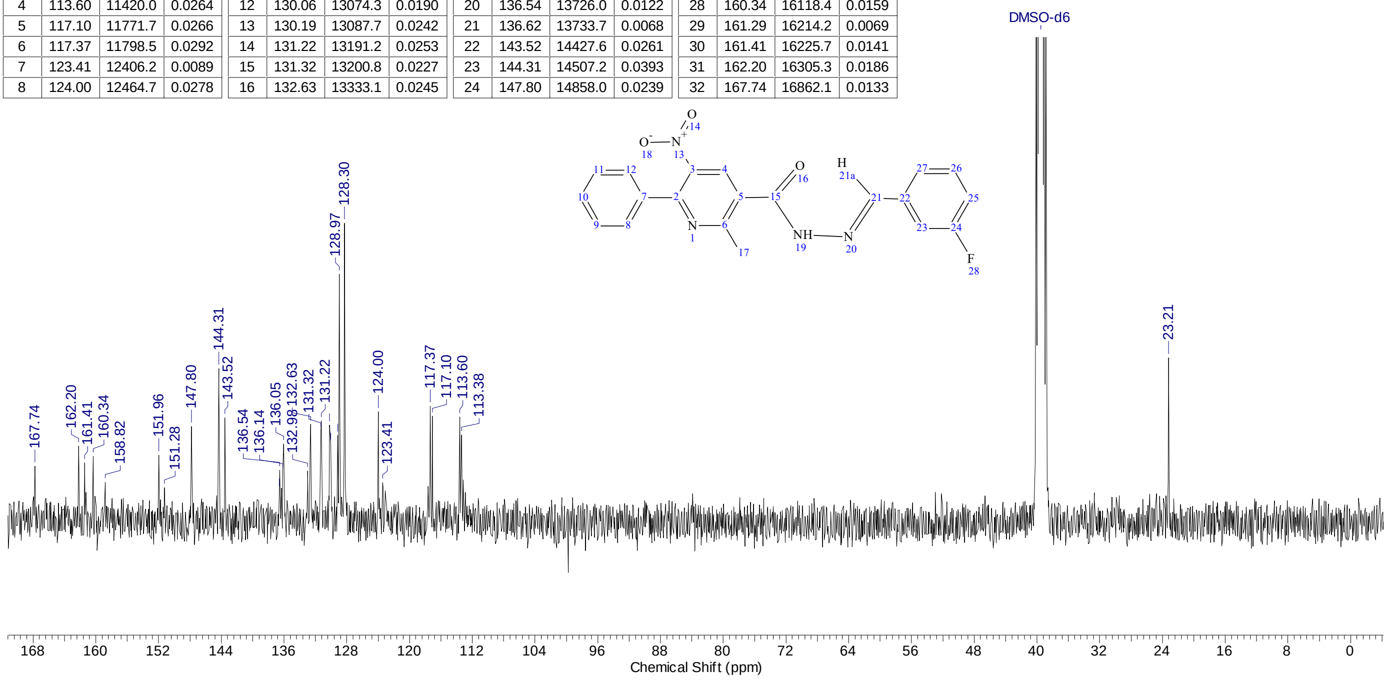

Figure S7: ^{1}H NMR (400 MHz, DMSO-d_6) and ^{13}C NMR (100 MHz, DMSO-d_6) spectra of **9**

No.	(ppm)	(Hz)	Height
1	2.58	1030.3	0.0294
2	2.70	1078.8	0.0570
3	7.19	2873.1	0.0102
4	7.29	2915.6	0.0204
5	7.51	3001.3	0.0831
6	7.81	3123.6	0.0196
7	8.13	3249.5	0.0096
8	8.29	3315.9	0.0194
9	8.47	3385.0	0.0083
10	8.57	3425.8	0.0174
11	12.16	4861.5	0.0014

No.	Atom	Exp. Shift (ppm)
1	17	[2.53 .. 2.77]
2	26	[7.14 .. 7.33]
3	24	[7.14 .. 7.33]
4	11	[7.46 .. 7.56]
5	12	[7.46 .. 7.56]
6	10	[7.46 .. 7.56]
7	8	[7.46 .. 7.56]
8	9	[7.46 .. 7.56]
9	27	[7.56 .. 7.87]
10	23	[7.56 .. 7.87]
11	21a	[8.09 .. 8.36]
12	4	[8.43 .. 8.60]
13	19	12.16

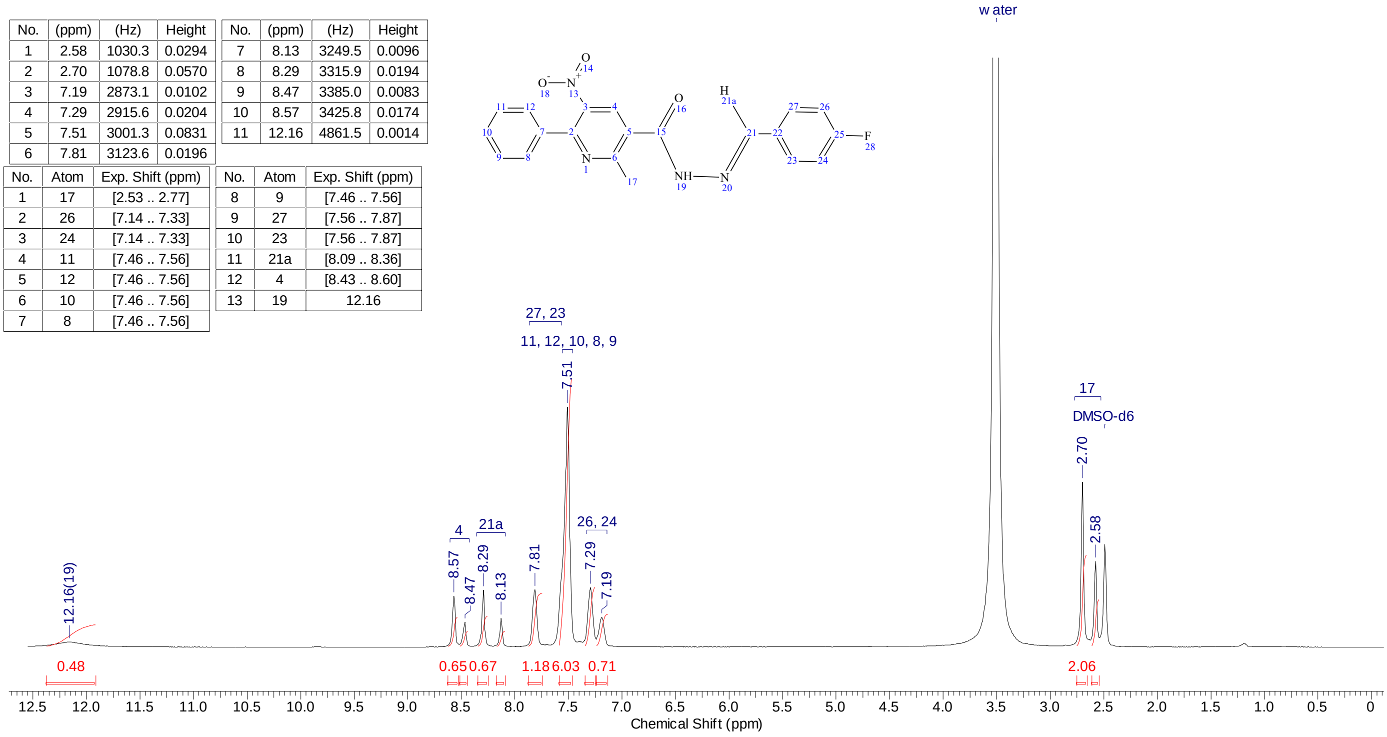

No.	(ppm)	(Hz)	Height
1	23.31	2343.3	0.0305
2	116.26	11687.4	0.0238
3	116.50	11711.3	0.0297
4	128.40	12907.5	0.0524
5	129.11	12978.4	0.0481
6	129.35	13003.3	0.0233
7	129.96	13064.7	0.0261
8	130.04	13072.3	0.0278
9	130.20	13088.6	0.0112
10	130.35	13103.0	0.0171
11	130.56	13124.1	0.0056
12	130.70	13138.5	0.0147
13	132.69	13338.8	0.0188
14	132.95	13364.7	0.0083
15	136.16	13687.7	0.0131
16	136.26	13697.3	0.0051
17	143.64	14439.1	0.0183
18	144.94	14570.4	0.0078
19	148.30	14907.8	0.0210
20	152.09	15289.3	0.0166
21	158.95	15978.4	0.0052
22	160.46	16129.9	0.0174
23	162.30	16314.8	0.0165
24	162.51	16335.9	0.0084

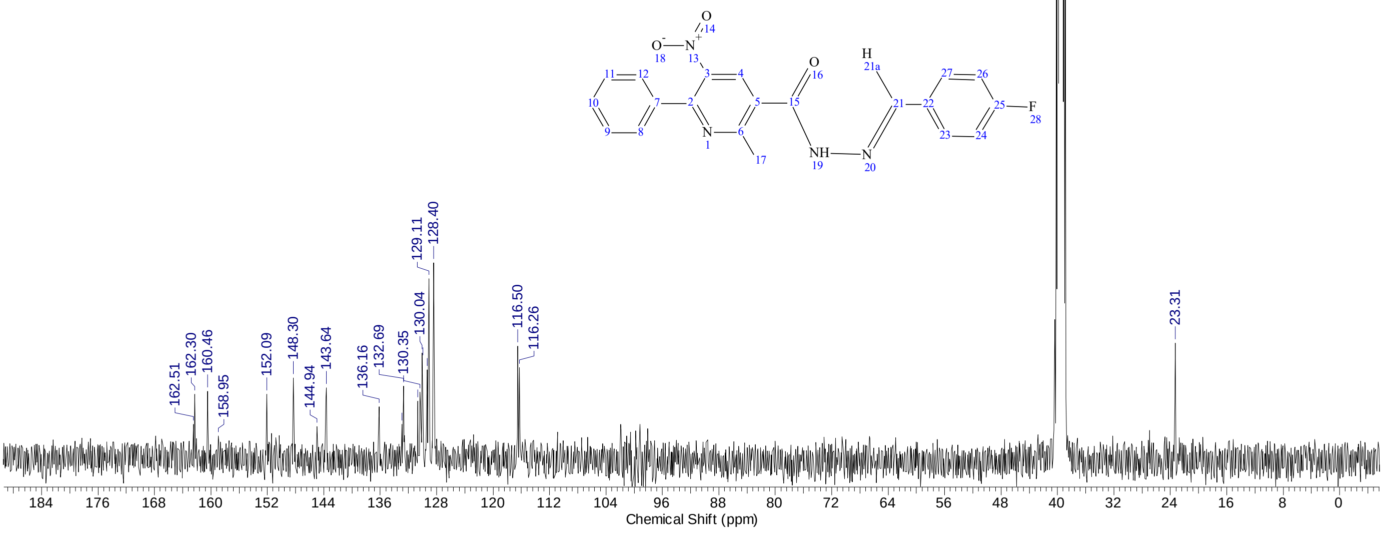

Figure S8: ^{1}H NMR (400 MHz, DMSO-d_6) and ^{13}C NMR (100 MHz, DMSO-d_6) spectra of **10**

No.	(ppm)	(Hz)	Height
1	2.49	995.5	0.2209
2	2.57	1027.1	0.3781
3	2.60	1038.5	0.4387
4	2.70	1080.2	0.2962
5	2.73	1091.6	0.3449
6	3.38	1351.7	1.0000
7	7.19	2874.4	0.0894
8	7.21	2882.7	0.1057
9	7.24	2893.2	0.0545

No.	(ppm)	(Hz)	Height
10	7.32	2926.2	0.1852
11	7.34	2933.9	0.2086
12	7.37	2946.8	0.1610
13	7.39	2952.7	0.1574
14	7.41	2961.0	0.1603
15	7.44	2973.3	0.1318
16	7.51	3000.8	0.8069
17	8.34	3335.6	0.1273
18	8.37	3347.0	0.1491

No.	(ppm)	(Hz)	Height
19	8.44	3375.0	0.1341
20	8.47	3386.4	0.1537
21	8.54	3413.9	0.1024
22	8.57	3425.8	0.1159
23	8.64	3455.6	0.1021
24	8.67	3467.5	0.1179
25	12.38	4950.7	0.0726

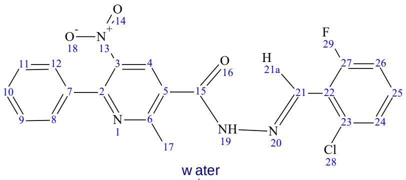

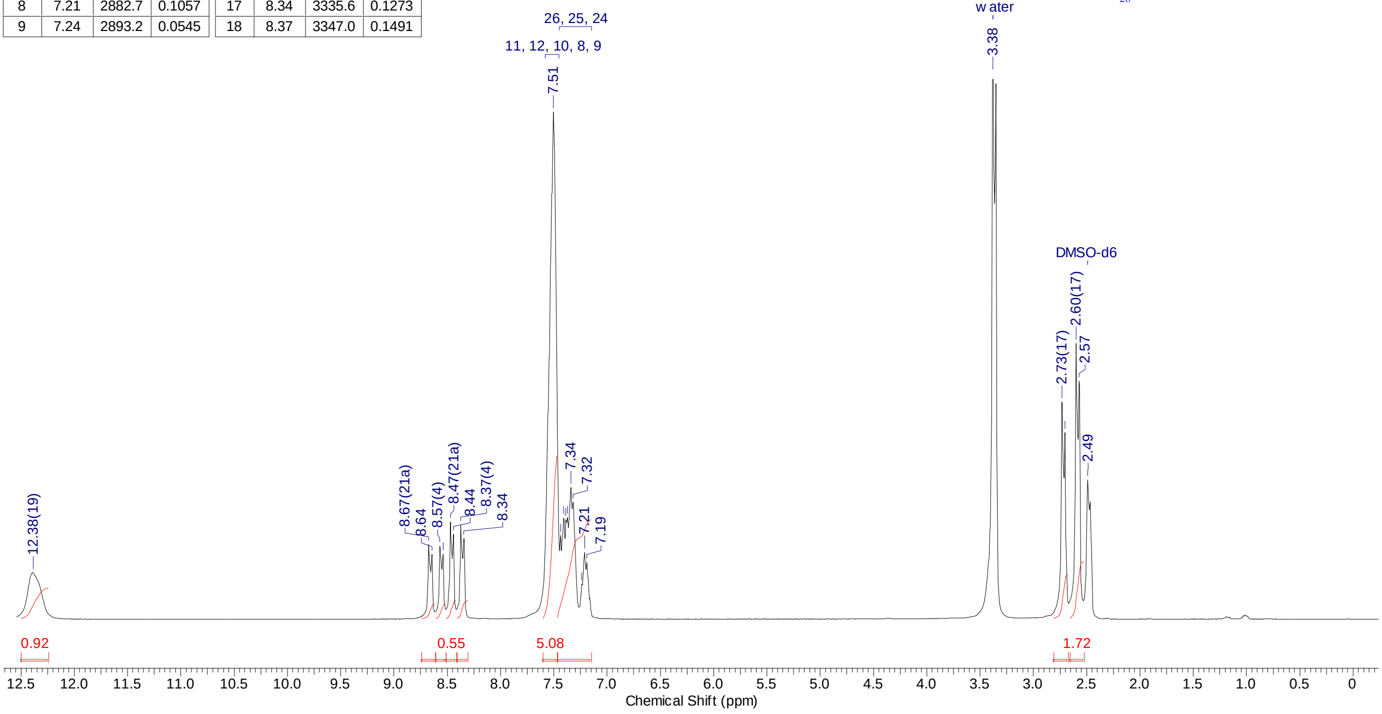

No.	(ppm)	(Hz)	Height
1	22.77	2288.6	0.0181
2	23.06	2318.4	0.0186
3	115.55	11615.5	0.0195
4	115.77	11637.5	0.0151
5	119.74	12037.2	0.0145
6	119.87	12049.7	0.0142
7	126.11	12677.5	0.0163
8	126.30	12696.6	0.0214

No.	(ppm)	(Hz)	Height
9	128.04	12871.1	0.0585
10	128.75	12943.0	0.0703
11	129.95	13062.8	0.0397
12	132.46	13315.8	0.0359
13	132.62	13332.1	0.0258
14	133.32	13402.1	0.0224
15	134.06	13476.8	0.0135
16	135.83	13654.1	0.0193

No.	(ppm)	(Hz)	Height
17	136.00	13671.4	0.0245
18	138.18	13890.9	0.0144
19	138.35	13907.2	0.0159
20	142.17	14291.5	0.0143
21	142.36	14310.7	0.0102
22	143.25	14399.8	0.0303
23	143.34	14409.4	0.0221
24	150.93	15172.3	0.0228

No.	(ppm)	(Hz)	Height
25	151.68	15248.1	0.0187
26	158.54	15937.2	0.0264
27	159.10	15993.8	0.0116
28	160.27	16111.6	0.0192
29	161.68	16252.5	0.0297
30	167.72	16860.2	0.0268

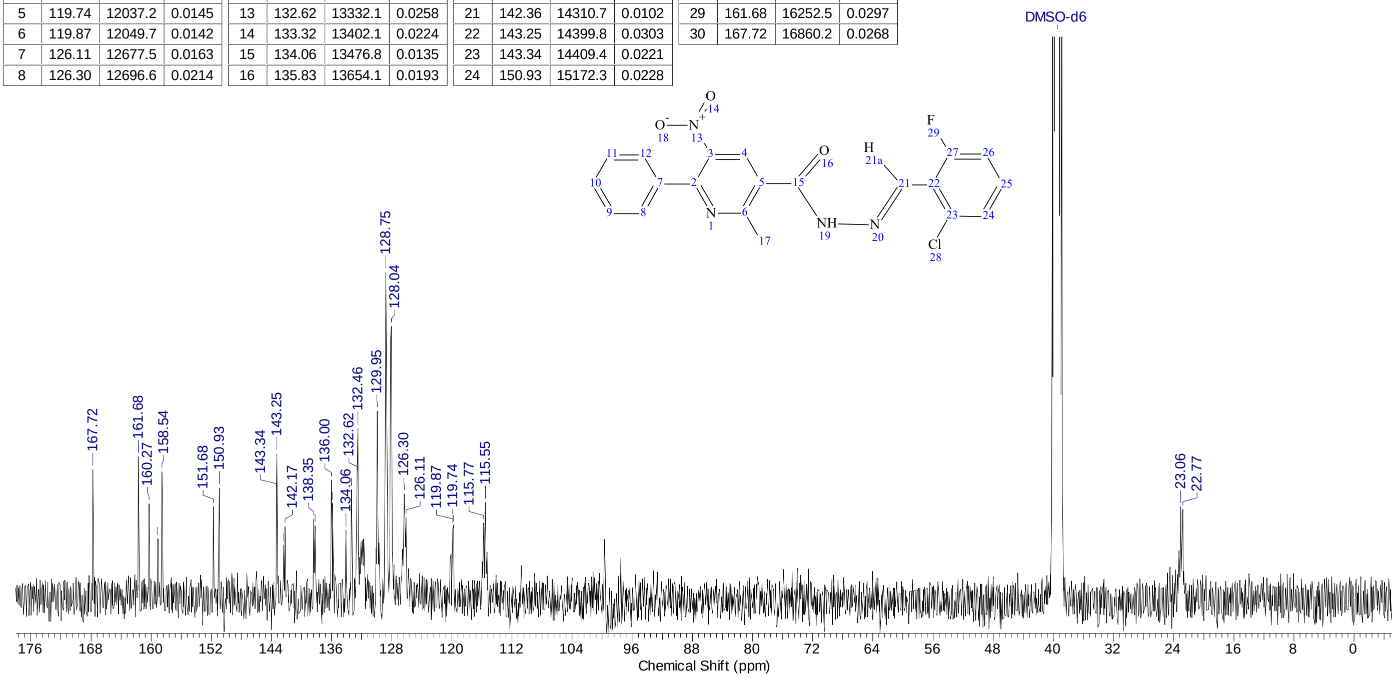

Figure S9: ^{1}H NMR (400 MHz, DMSO-d_6) and ^{13}C NMR (100 MHz, DMSO-d_6) spectra of **11**

No.	(ppm)	(Hz)	Height	No.	(ppm)	(Hz)	Height	No.	(ppm)	(Hz)	Height
1	2.49	995.5	0.2057	10	7.50	2999.4	0.8321	19	8.31	3320.5	0.1777
2	2.57	1027.5	0.2558	11	7.52	3006.8	0.8949	20	8.48	3391.9	0.0906
3	2.59	1035.8	0.2820	12	7.54	3015.9	0.5130	21	8.51	3400.6	0.1008
4	2.70	1078.4	0.4880	13	7.76	3101.1	0.1819	22	8.60	3436.3	0.1708
5	2.72	1087.1	0.5392	14	7.78	3109.4	0.3313	23	8.62	3444.6	0.1875
6	7.41	2963.3	0.1835	15	7.80	3117.1	0.1701	24	12.18	4868.8	0.1227
7	7.43	2971.5	0.1541	16	8.12	3244.9	0.0853	25	12.31	4921.4	0.0546
8	7.45	2977.5	0.1553	17	8.14	3253.1	0.0936				
9	7.47	2986.2	0.2581	18	8.28	3311.8	0.1625				

No.	Atom	Exp. Shift (ppm)	No.	Atom	Exp. Shift (ppm)
1	17	[2.54 .. 2.75]	8	10	[7.38 .. 7.61]
2	12	[7.38 .. 7.61]	9	27	[7.73 .. 7.82]
3	24	[7.38 .. 7.61]	10	23	[7.73 .. 7.82]
4	26	[7.38 .. 7.61]	11	21a	[8.09 .. 8.33]
5	11	[7.38 .. 7.61]	12	4	[8.45 .. 8.64]
6	8	[7.38 .. 7.61]	13	19	[12.12 .. 12.35]
7	9	[7.38 .. 7.61]			

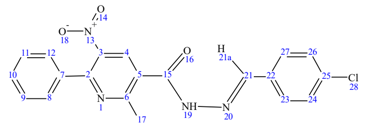

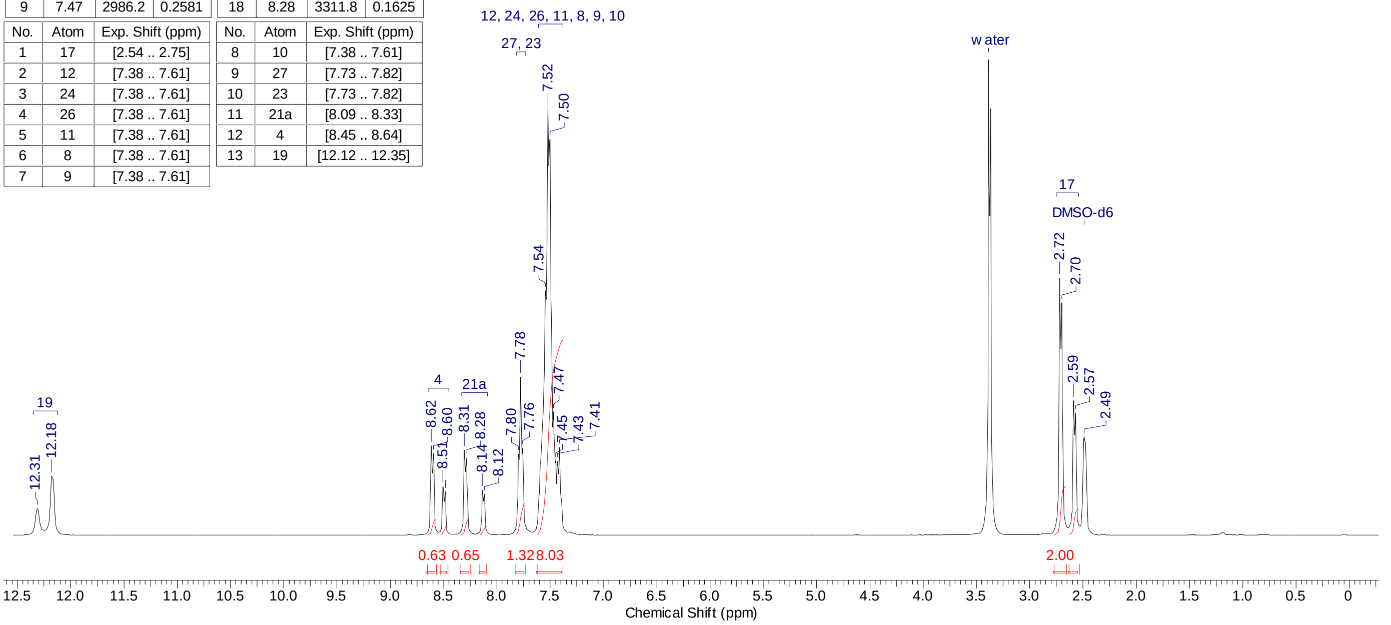

No.	(ppm)	(Hz)	Height	No.	(ppm)	(Hz)	Height	No.	(ppm)	(Hz)	Height
1	23.06	2318.4	0.0932	10	129.95	13062.8	0.0422	19	143.36	14411.3	0.0479
2	128.10	12877.8	0.1390	11	132.44	13313.9	0.0528	20	144.22	14497.6	0.0247
3	128.21	12888.3	0.0780	12	132.60	13329.2	0.0224	21	147.45	14822.5	0.0544
4	128.48	12915.2	0.0591	13	132.72	13341.7	0.0289	22	151.00	15179.1	0.0159
5	128.64	12931.5	0.0816	14	132.85	13355.1	0.0360	23	151.60	15239.4	0.0326
6	128.73	12941.0	0.1390	15	134.67	13538.2	0.0199	24	158.49	15932.4	0.0170
7	128.98	12966.0	0.1560	16	134.97	13567.9	0.0311	25	160.09	16093.4	0.0353
8	129.04	12971.7	0.1639	17	135.87	13658.0	0.0333	26	161.74	16259.3	0.0387
9	129.80	13048.4	0.0262	18	136.01	13672.3	0.0195	27	167.44	16831.5	0.0226

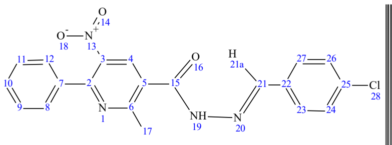

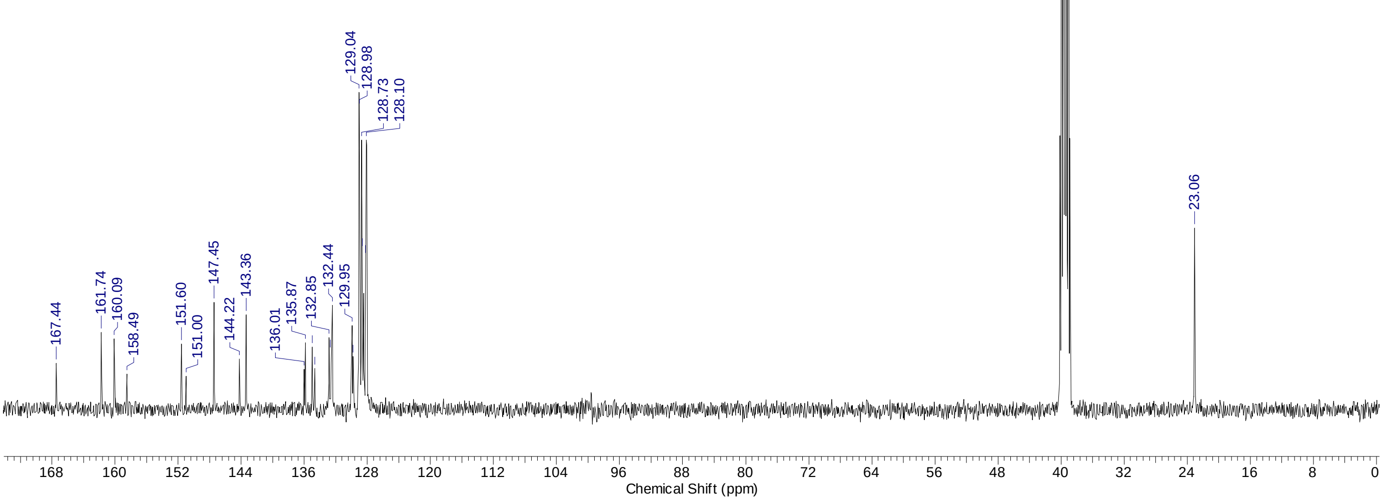

Figure S10.1: ^{1}H NMR (400 MHz, DMSO-d_6) and ^{13}C NMR (100 MHz, DMSO-d_6) spectra of **12**

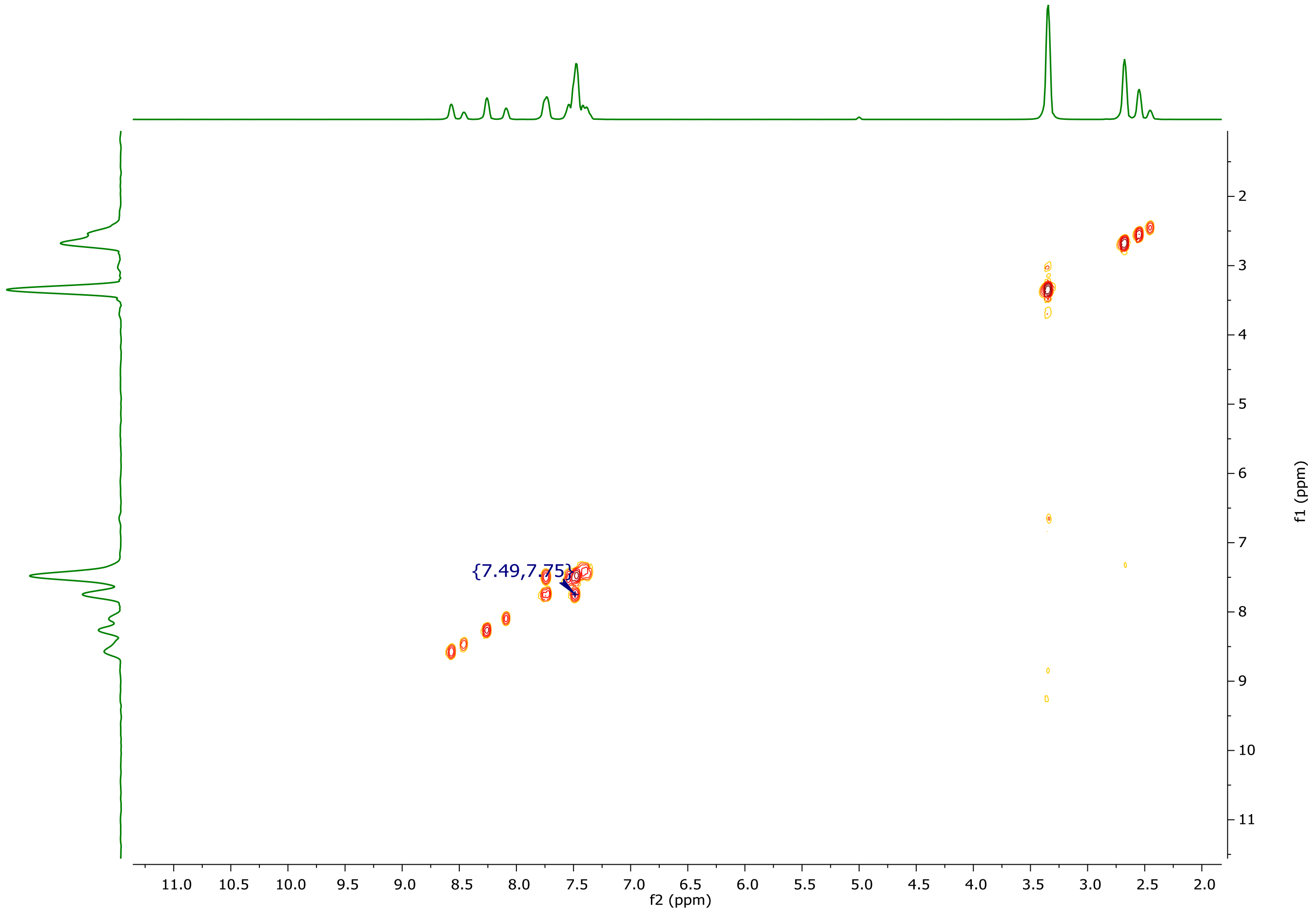

Figure S10.2: ^{1}H-^{1}H COSY spectra of **12**

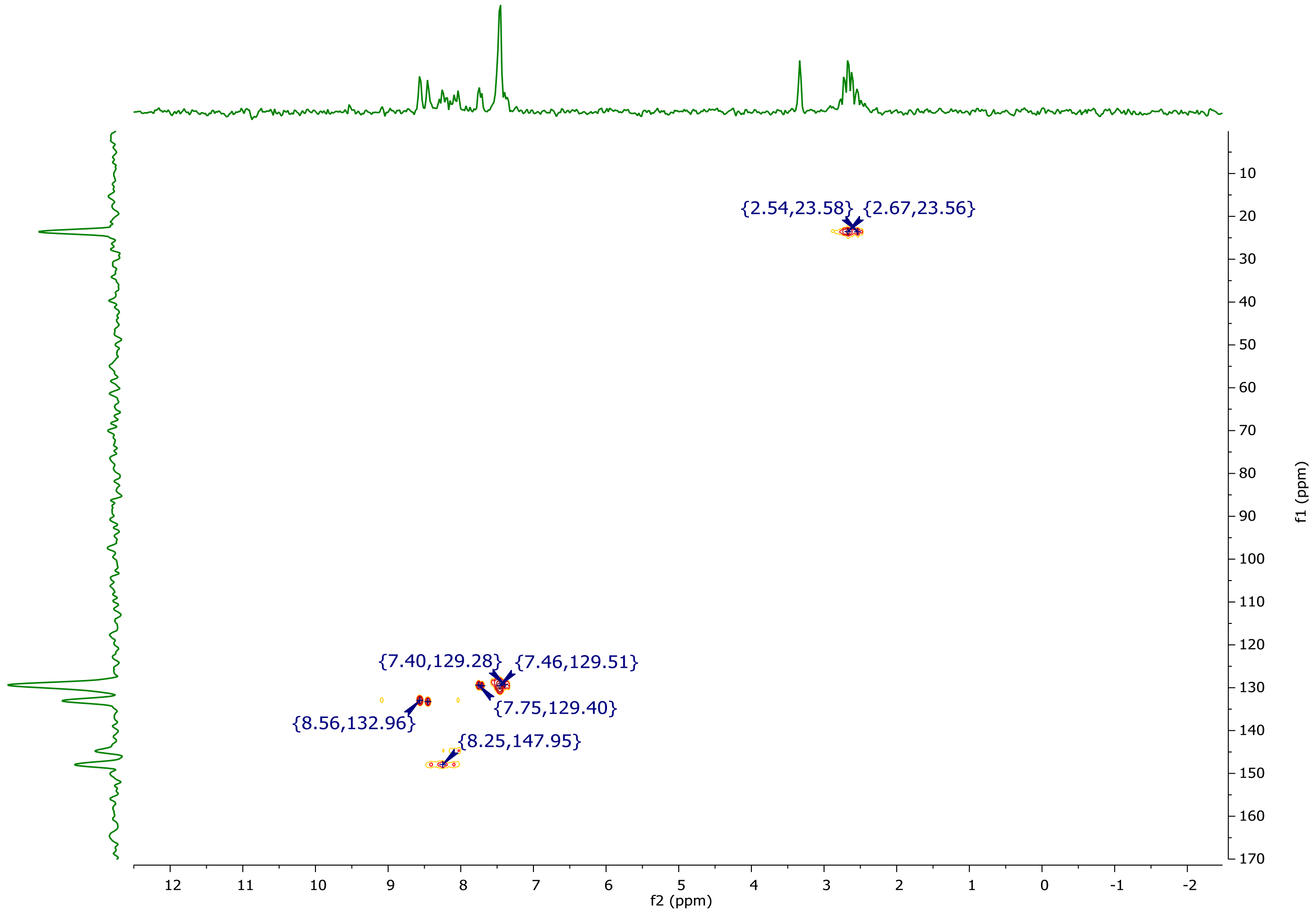

Figure S10.3: HMQC spectra of **12**

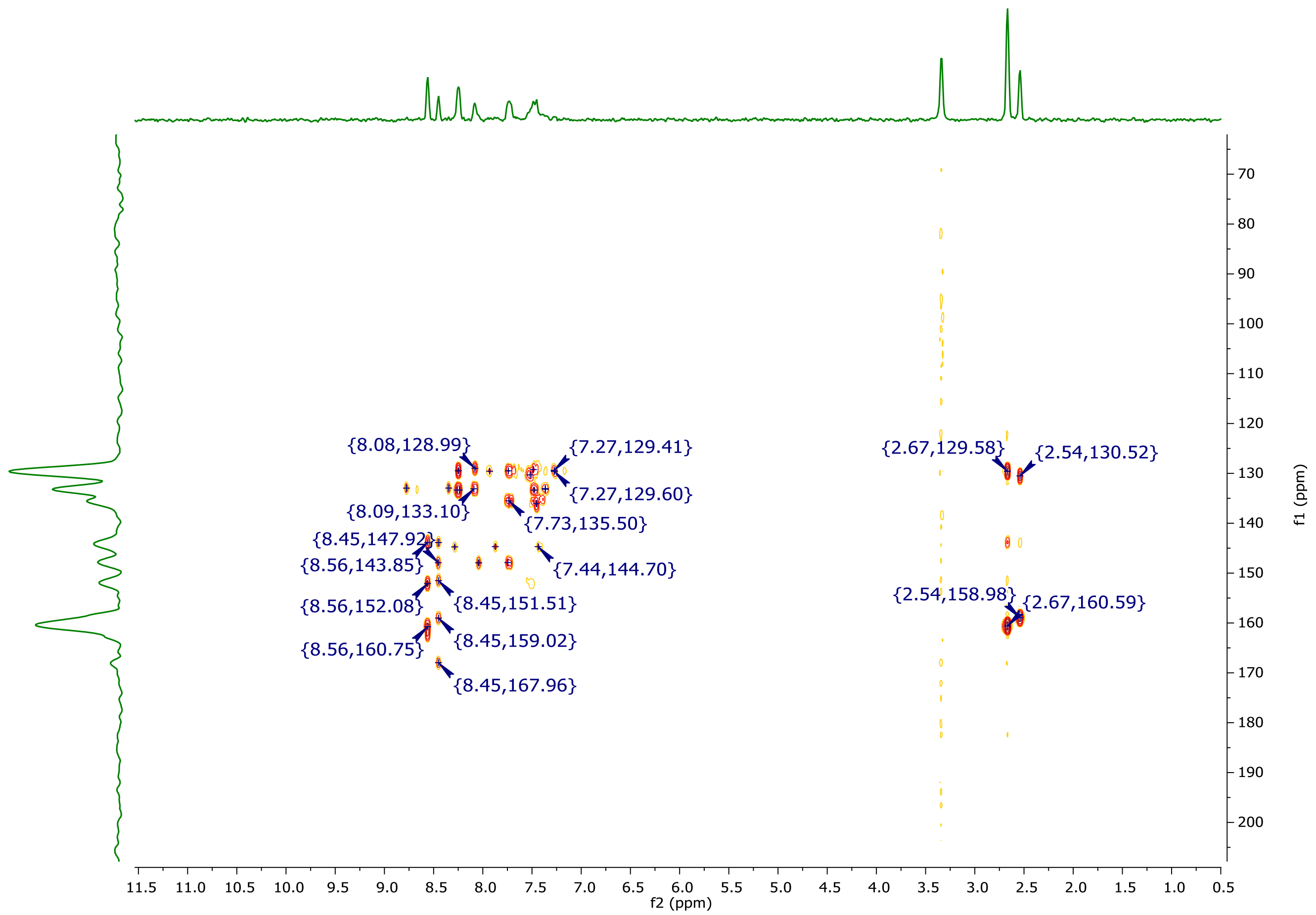

Figure S10.4: HMBC spectra of **12**

No.	(ppm)	(Hz)	Height
1	2.59	1036.2	0.4187
2	2.60	1039.9	0.4136
3	2.72	1087.5	0.6840
4	2.73	1091.2	0.7410
5	7.39	2956.4	0.2457
6	7.52	3004.5	1.0000
7	7.56	3021.4	0.4027

No.	(ppm)	(Hz)	Height
8	7.56	3024.2	0.3870
9	7.59	3035.2	0.2455
10	7.60	3038.8	0.2321
11	7.70	3078.7	0.4137
12	8.12	3247.2	0.1316
13	8.13	3250.8	0.1292
14	8.30	3320.0	0.2253

No.	(ppm)	(Hz)	Height
15	8.31	3323.7	0.2174
16	8.53	3408.8	0.1558
17	8.56	3421.2	0.2791
18	8.63	3451.4	0.2545
19	8.64	3455.5	0.2681
20	8.67	3467.9	0.4103
21	12.39	4954.4	0.0697

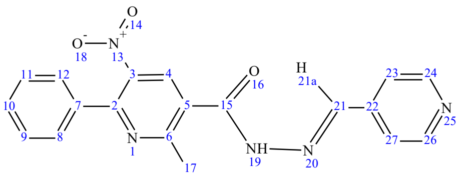

No.	Atom	Exp. Shift (ppm)
1	17	[2.56 .. 2.79]
2	27	[7.36 .. 7.43]
3	23	[7.36 .. 7.43]
4	11	[7.49 .. 7.60]
5	12	[7.49 .. 7.60]
6	10	[7.49 .. 7.60]
7	8	[7.49 .. 7.60]
8	9	[7.49 .. 7.60]
9	27	[7.67 .. 7.73]

No.	Atom	Exp. Shift (ppm)
10	23	[7.67 .. 7.73]
11	21a	[8.09 .. 8.36]
12	4	[8.51 .. 8.54]
13	26	[8.55 .. 8.58]
14	24	[8.55 .. 8.58]
15	4	[8.62 .. 8.65]
16	26	[8.66 .. 8.69]
17	24	[8.66 .. 8.69]
18	19	12.39

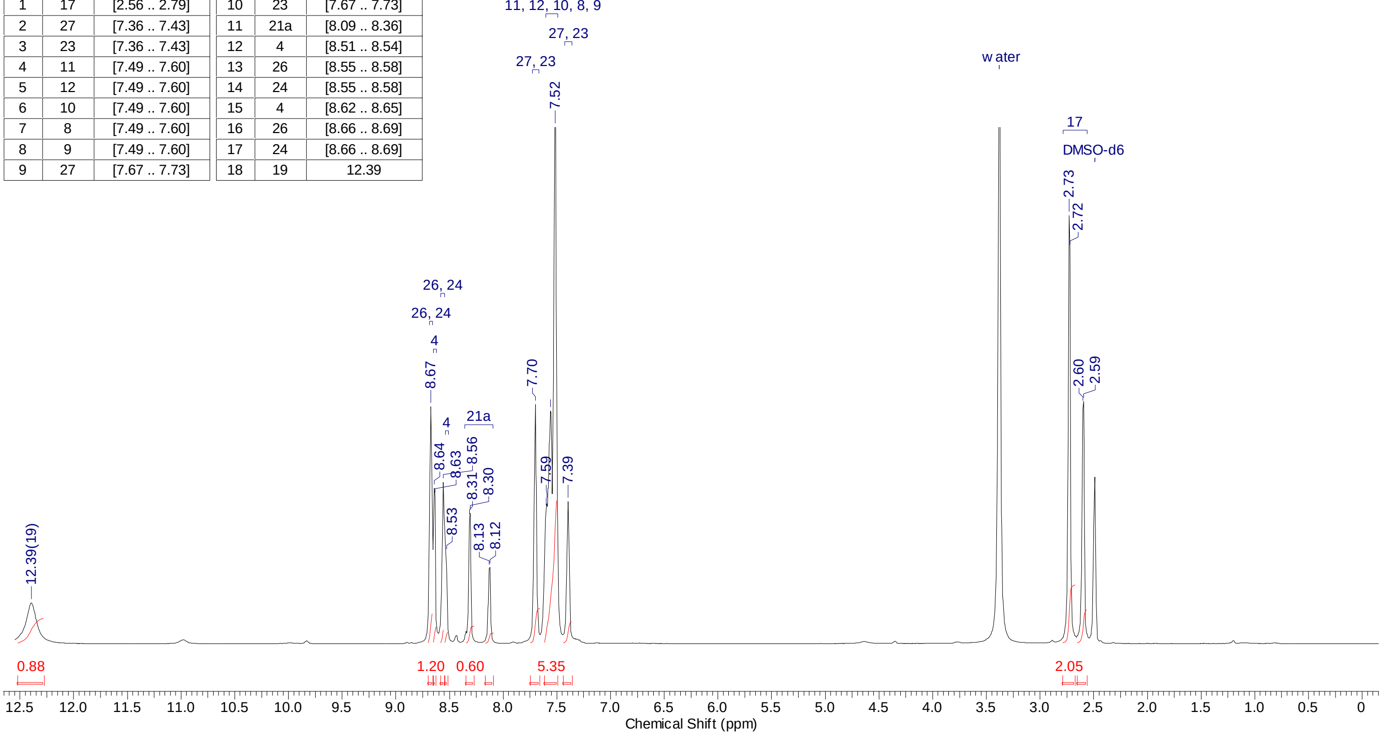

No.	(ppm)	(Hz)	Height
1	23.03	2315.5	0.0858
2	120.71	12134.0	0.0569
3	121.14	12178.1	0.1111
4	128.08	12874.9	0.1569
5	128.18	12885.4	0.0843
6	128.60	12927.6	0.0888
7	128.70	12937.2	0.1531
8	128.82	12949.7	0.0402
9	129.64	13032.1	0.0234

No.	(ppm)	(Hz)	Height
10	129.77	13045.5	0.0275
11	129.93	13060.8	0.0469
12	132.49	13318.7	0.0582
13	132.81	13351.3	0.0293
14	135.82	13653.2	0.0347
15	135.98	13669.5	0.0213
16	140.77	14150.6	0.0225
17	141.07	14181.3	0.0372
18	143.04	14378.7	0.0269

No.	(ppm)	(Hz)	Height
19	143.32	14407.5	0.0716
20	146.40	14717.1	0.0540
21	150.35	15113.9	0.1260
22	151.12	15191.5	0.0176
23	151.72	15251.9	0.0323
24	160.12	16096.3	0.0331
25	161.98	16283.2	0.0400
26	167.72	16860.2	0.0211

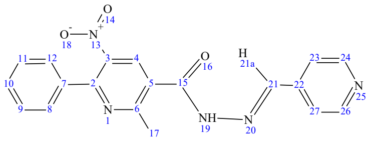

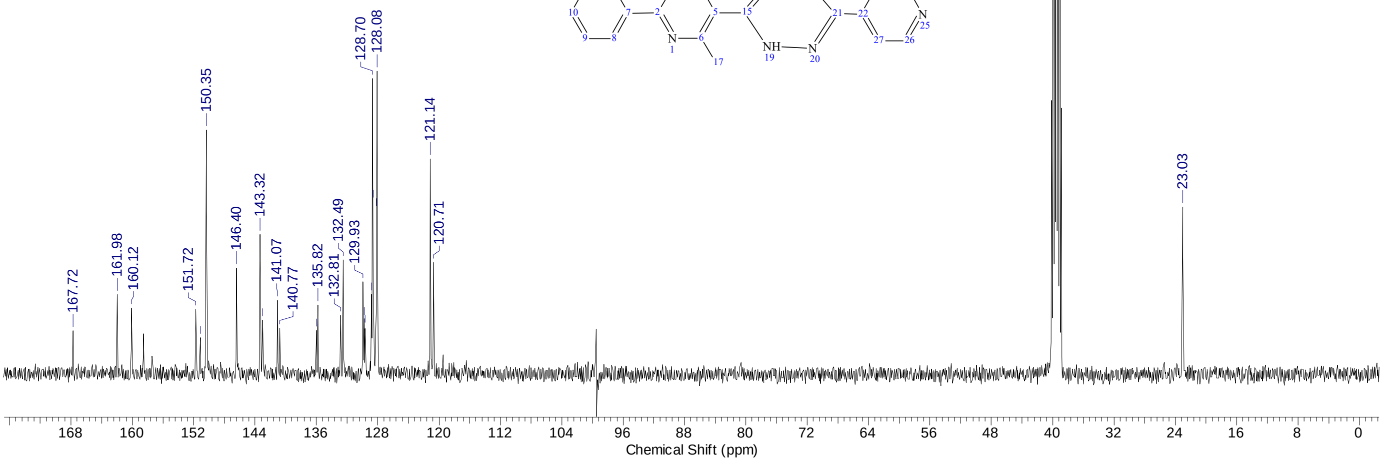

Figure S11.1: ^{1}H NMR (400 MHz, DMSO-d_6) and ^{13}C NMR (100 MHz, DMSO-d_6) spectra of **13**

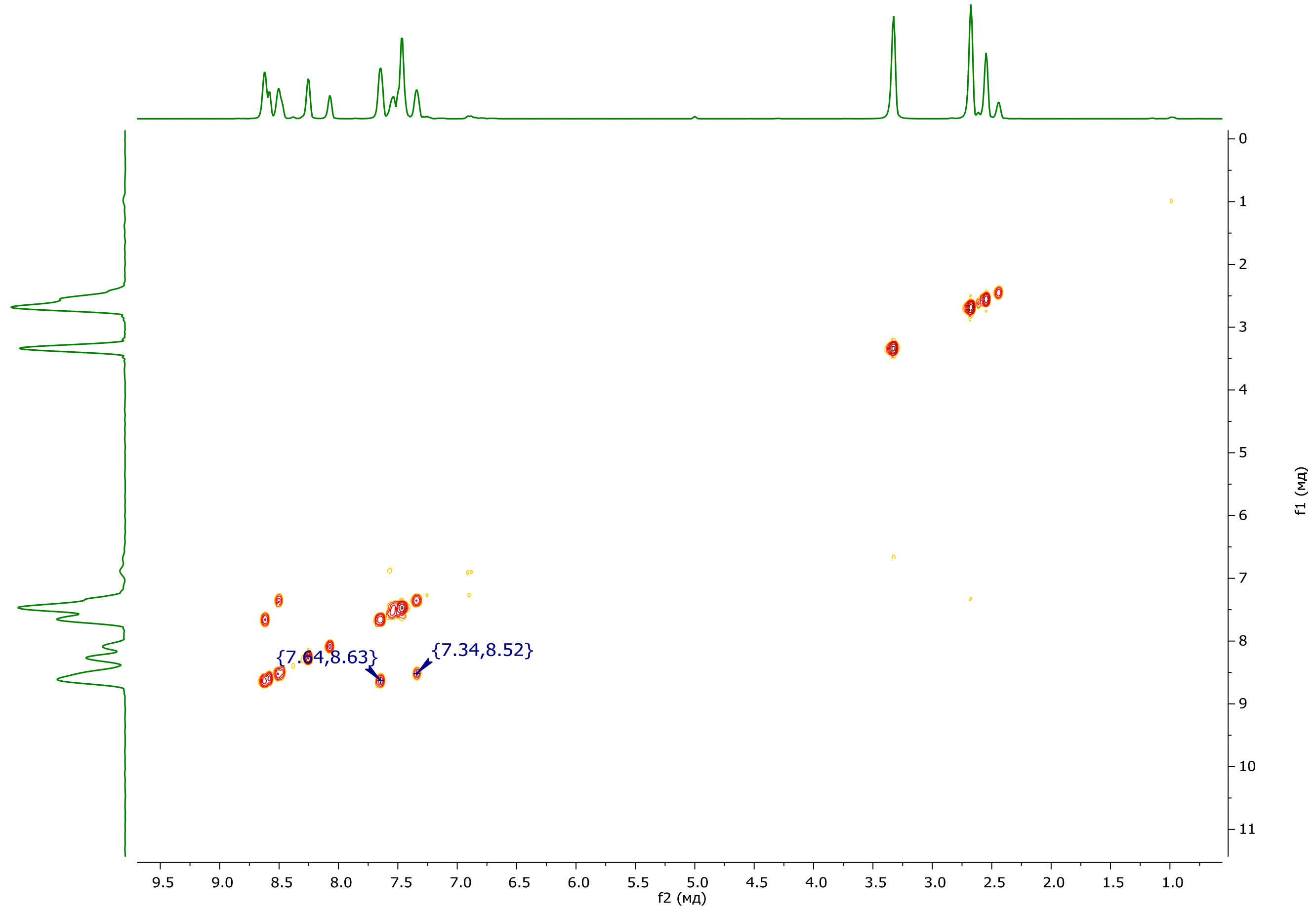

Figure S11.2: ^{1}H-^{1}H COSY spectra of **13**

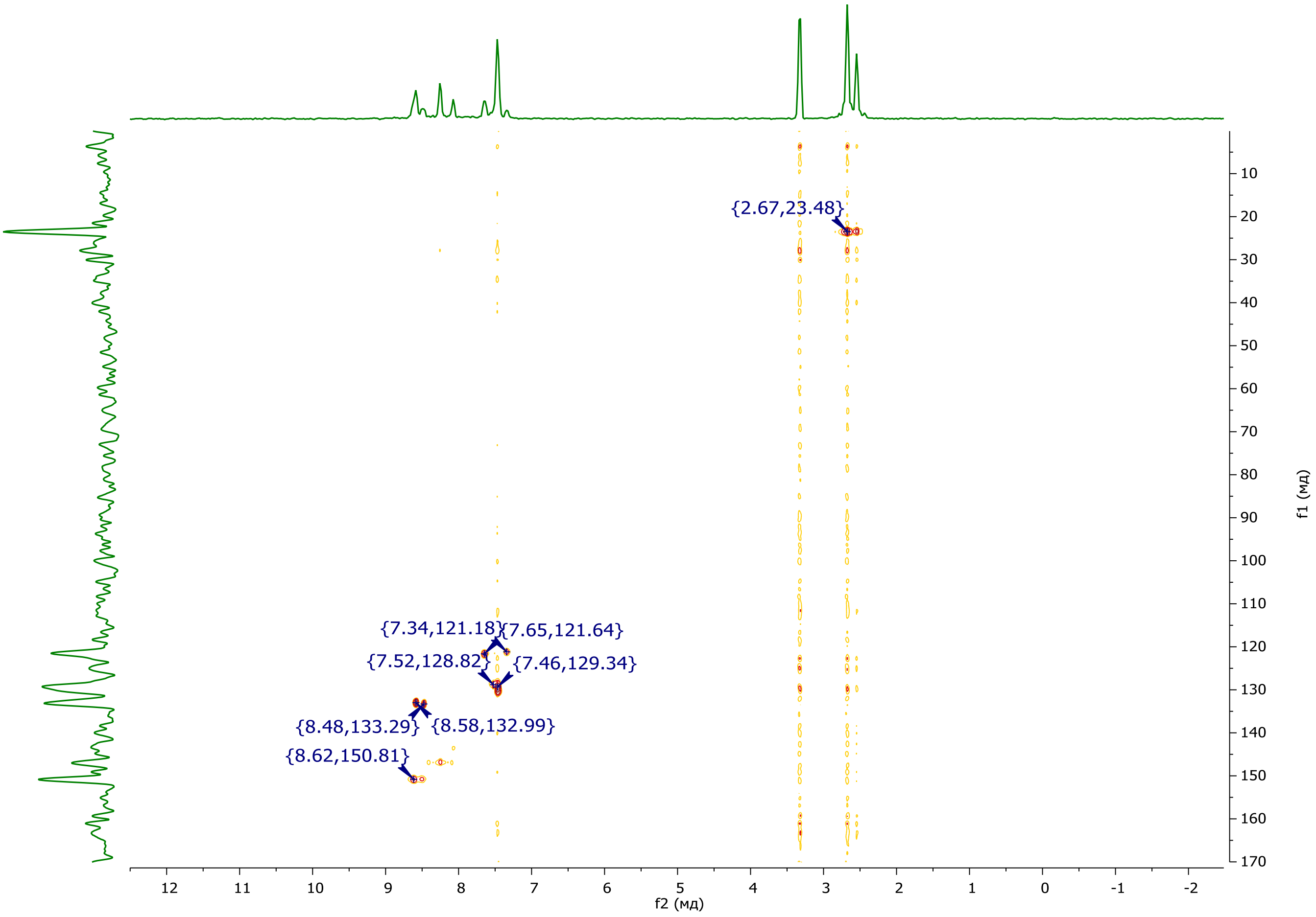

Figure S11.3: HMQC spectra of **13**

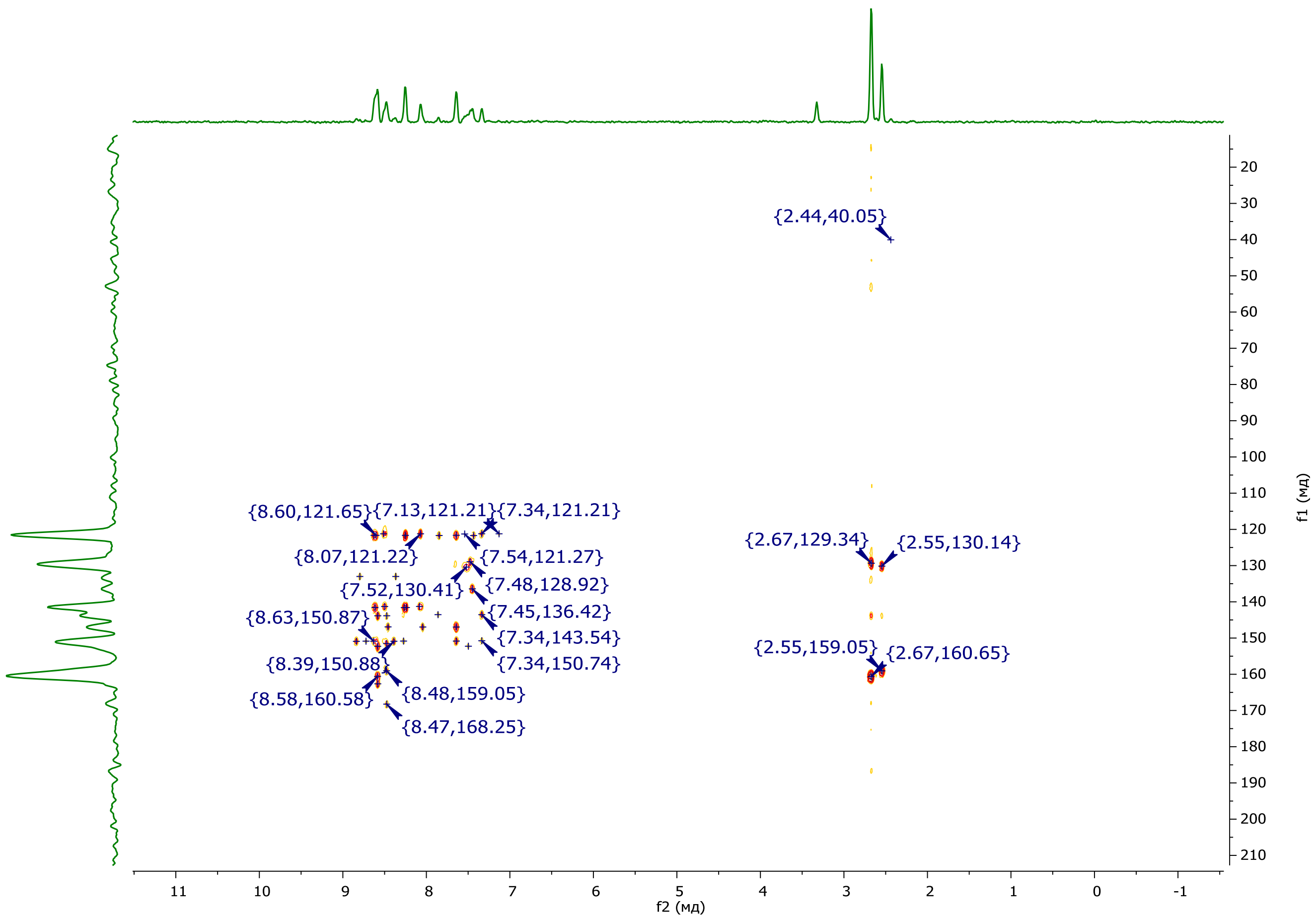

Figure S11.4: HMBC spectra of **13**

3 DFT calculations

3.1 Analysis of thermodynamic data for configurations and conformations

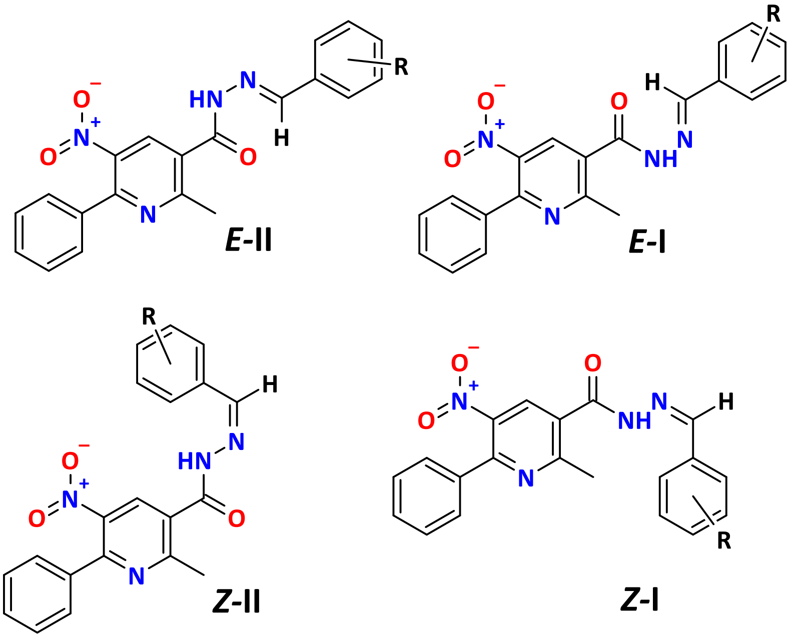

Figure S12: 2D structures of possible configurations and conformations for **4–13**

Table S.1: Gibbs free energy, Δ*G* (kcal/mol) differences in values for corresponding configurations and conformations of **4–13**.

	Z-I – *E*-I	*Z*-II – *E*-II	*Z*-I – *Z*-II	*E*-I – *E*-II
**4**	9.7122	8.4928	1.5048	0.2854
**5**	9.8790	8.6727	1.4655	0.2591
**6**	6.2813	6.0992	0.5161	0.3340
**7**	7.9526	6.3745	1.8893	0.3112
**8**	3.5000	3.2907	0.4714	0.2621
**9**	4.5344	4.5057	0.4162	0.3875
**10**	4.3476	4.2507	0.4082	0.3114
**11**	1.7511	1.3521	0.7207	0.3218
**12**	4.5131	4.3250	0.4978	0.3097
**13**	4.8315	4.7394	0.4351	0.3431

* *{Value*1*}* > *{Value*2*}*.

Table S.2: Conformational constants, *K* for II→I-transition and equilibrium content of **4–13**.

	$K_{II(Z)\rightarrow I(Z)}$*	$K_{II(E)\rightarrow I(E)}$*	*Z*-II (%)	*Z*-I (%)	*E*-II (%)	*E*-I (%)
**4**	0.0789	0.6177	92.7	7.3	61.8	38.2
**5**	0.0843	0.6457	92.2	7.8	60.8	39.2
**6**	0.4185	0.5691	70.5	29.5	63.7	36.3
**7**	0.0412	0.5915	96.0	4.0	62.8	37.2
**8**	0.4513	0.6425	68.9	31.1	60.9	39.1
**9**	0.4954	0.5199	66.9	33.1	65.8	34.2
**10**	0.5021	0.5912	66.6	33.4	62.8	37.2
**11**	0.2963	0.5810	77.1	22.9	63.3	36.7
**12**	0.4316	0.5929	69.9	30.1	62.8	37.2
**13**	0.4798	0.5604	67.6	32.4	64.1	35.9

* On a rough assumption, value of *K* can be obtained from $\Delta G = -RT\ln K$.

3.2 NMR summary data for configurations and conformations

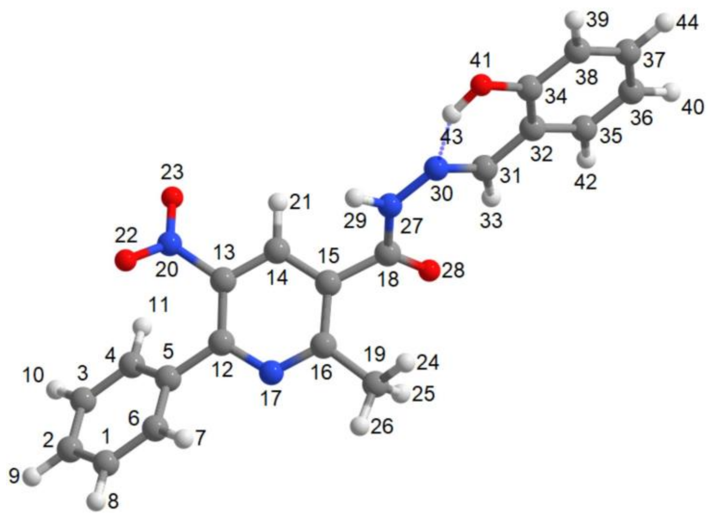

Figure S13: Optimized geometry of **4**, obtained using B3LYP-D3(BJ)/def2-TZVPP level of theory in DMSO with the help of the CPCM continuum solvation model

Table S.3: ^{1}H and ^{13}C NMR chemical shifts (TMS, ppm) for **4**, obtained using GIAO//B3LYP-D3(BJ)/def2-TZVPP level of theory in DMSO with the help of the CPCM continuum solvation model.

Atom	δ (Z-I)	δ (Z-II)	δ (E-I)	δ (E-II)	Atom	δ (Z-I)	δ (Z-II)	δ (E-I)	δ (E-II)
7 H	7.76	8.41	8.40	8.41	1 C	135.71	136.83	136.81	136.80
8 H	7.84	8.09	8.10	8.10	2 C	138.58	138.47	138.23	138.25
9 H	8.00	8.00	8.00	8.00	3 C	136.80	135.73	135.69	135.63
10 H	8.09	7.84	7.85	7.85	4 C	137.32	136.78	136.60	136.89
11 H	8.44	7.77	7.76	7.76	5 C	145.75	145.95	146.17	146.46
21 H	8.74	8.73	8.67	8.67	6 C	136.71	137.24	137.03	137.11
24 H	3.28	2.56	2.90	2.79	12 C	165.16	165.32	165.16	165.56
25 H	2.93	2.92	3.16	3.26	13 C	153.64	153.24	154.01	152.68
26 H	3.33	2.52	2.78	2.68	14 C	145.42	140.11	143.77	140.88
29 H	9.23	9.37	9.01	9.15	15 C	136.70	135.09	138.48	136.77
33 H	8.84	8.86	10.84	10.81	16 C	170.59	174.48	170.29	173.99
39 H	7.33	7.38	7.34	7.34	18 C	173.41	172.39	175.37	175.38
40 H	7.46	7.48	7.36	7.36	19 C	29.90	28.01	27.59	28.24
42 H	7.89	7.93	7.85	7.85	31 C	170.98	173.68	164.73	164.43
43 H	6.83	7.04	11.96	11.99	32 C	130.20	129.63	125.95	125.97
44 H	7.87	7.94	7.81	7.81	34 C	164.87	165.57	170.49	170.50
					35 C	140.62	141.99	141.11	141.05
					36 C	126.40	126.32	125.38	125.35
					37 C	141.23	141.90	140.74	140.71
					38 C	123.64	124.75	122.54	122.58

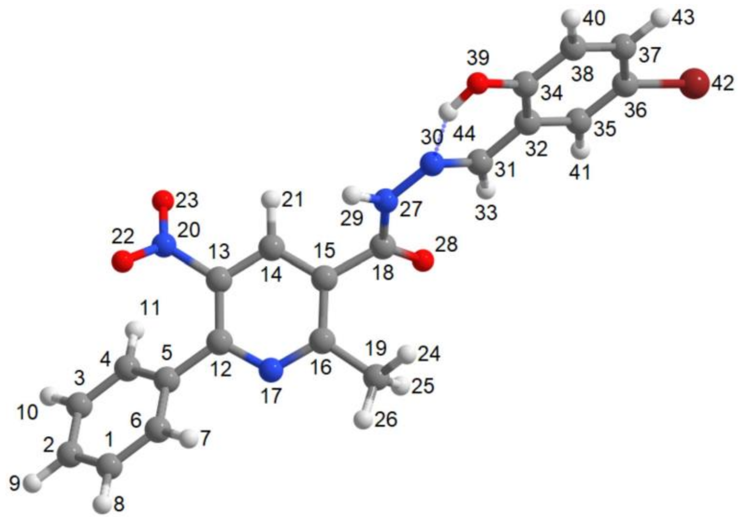

Figure S14: Optimized geometry of **5**, obtained using B3LYP-D3(BJ)/def2-TZVPP level of theory in DMSO with the help of the CPCM continuum solvation model

Table S.4: ^{1}H and ^{13}C NMR chemical shifts (TMS, ppm) for **5**, obtained using GIAO//B3LYP-D3(BJ)/def2-TZVPP level of theory in DMSO with the help of the CPCM continuum solvation model.

Atom	δ (*Z*-I)	δ (*Z*-II)	δ (*E*-I)	δ (*E*-II)	Atom	δ (*Z*-I)	δ (*Z*-II)	δ (*E*-I)	δ (*E*-II)
7 H	7.76	8.42	8.42	8.41	1 C	135.70	136.80	136.78	136.82
8 H	7.83	8.09	8.10	8.09	2 C	138.58	138.56	138.30	138.28
9 H	8.00	8.00	8.00	8.00	3 C	136.80	135.64	135.70	135.68
10 H	8.09	7.83	7.84	7.85	4 C	137.37	136.84	136.82	136.67
11 H	8.42	7.79	7.74	7.78	5 C	145.70	145.59	146.35	146.14
21 H	8.74	8.70	8.78	8.55	6 C	136.69	137.39	136.99	137.26
24 H	3.25	2.66	2.88	2.76	12 C	165.34	165.03	165.73	164.97
25 H	2.91	2.93	3.18	3.22	13 C	153.67	153.36	153.38	153.16
26 H	3.33	2.57	2.78	2.67	14 C	145.49	140.28	144.36	140.33
29 H	9.27	9.40	9.08	9.15	15 C	136.51	135.10	138.01	136.98
33 H	8.76	8.73	10.81	10.78	16 C	170.62	174.54	170.36	173.47
40 H	7.29	7.34	7.29	7.29	18 C	173.55	172.37	175.74	175.95
41 H	7.87	7.91	7.83	7.83	19 C	29.96	27.88	27.67	27.99
43 H	7.80	7.89	7.75	7.75	31 C	169.61	170.86	162.86	162.98
44 H	6.78	6.89	12.03	12.03	32 C	132.02	131.76	127.82	127.78
					34 C	163.83	164.85	169.49	169.51
					35 C	142.31	143.76	142.88	142.91
					36 C	137.06	136.65	135.46	135.47
					37 C	143.64	144.39	143.06	143.11
					38 C	125.95	126.51	124.74	124.75

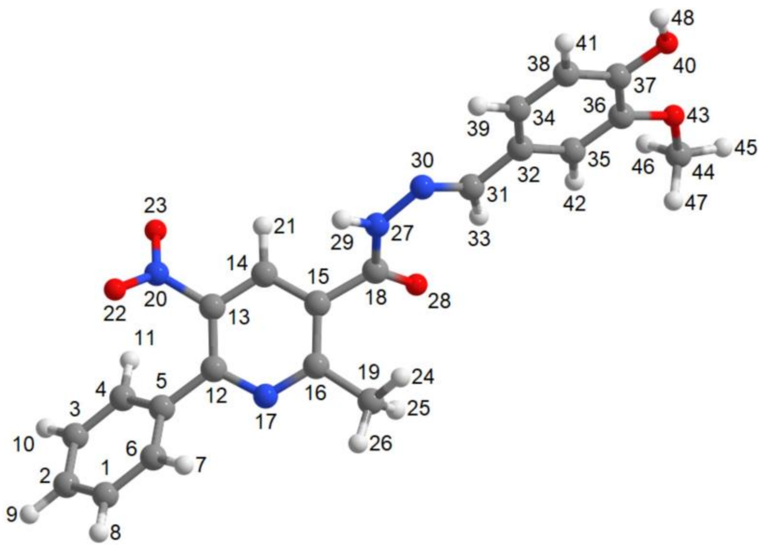

Figure S15: Optimized geometry of **6**, obtained using B3LYP-D3(BJ)/def2-TZVPP level of theory in DMSO with the help of the CPCM continuum solvation model

Table S.5: ^{1}H and ^{13}C NMR chemical shifts (TMS, ppm) for **6**, obtained using GIAO//B3LYP-D3(BJ)/def2-TZVPP level of theory in DMSO with the help of the CPCM continuum solvation model.

Atom	δ (Z-I)	δ (Z-II)	δ (E-I)	δ (E-II)	Atom	δ (Z-I)	δ (Z-II)	δ (E-I)	δ (E-II)
7 H	7.75	8.33	8.43	8.40	1 C	135.74	136.76	136.78	136.81
8 H	7.84	8.09	8.10	8.09	2 C	138.48	137.94	138.27	138.16
9 H	8.00	7.98	8.00	7.99	3 C	136.81	135.58	135.67	135.63
10 H	8.10	7.82	7.84	7.84	4 C	136.96	136.56	136.91	136.61
11 H	8.47	7.74	7.76	7.78	5 C	145.90	146.52	146.20	146.17
21 H	8.96	8.66	8.83	8.52	6 C	137.03	137.00	137.00	137.27
24 H	3.34	2.87	2.95	2.76	12 C	164.82	164.96	165.10	164.39
25 H	2.96	3.04	3.20	3.21	13 C	153.56	153.31	153.57	153.30
26 H	3.31	2.62	2.81	2.66	14 C	145.49	140.35	144.67	139.92
29 H	9.17	9.03	9.05	9.05	15 C	136.32	137.51	138.53	137.86
33 H	8.27	8.34	10.89	10.84	16 C	170.96	174.20	169.87	173.40
39 H	8.21	8.51	7.99	7.99	18 C	168.57	169.45	175.12	175.84
41 H	7.13	7.29	7.25	7.25	19 C	29.59	28.35	27.93	28.05
42 H	7.60	7.62	7.42	7.42	31 C	166.70	169.28	160.85	160.74
45 H	4.10	4.11	4.29	4.29	32 C	135.28	134.91	136.29	136.22
46 H	3.62	4.31	3.95	3.94	34 C	133.75	134.18	122.08	122.11
47 H	4.25	3.61	3.95	3.96	35 C	136.79	137.43	122.17	122.07
48 H	5.62	5.74	5.44	5.45	36 C	155.82	155.91	156.67	156.71
					37 C	163.27	163.73	158.44	158.44
					38 C	122.52	123.11	122.13	122.05
					44 C	65.42	65.22	59.02	58.98

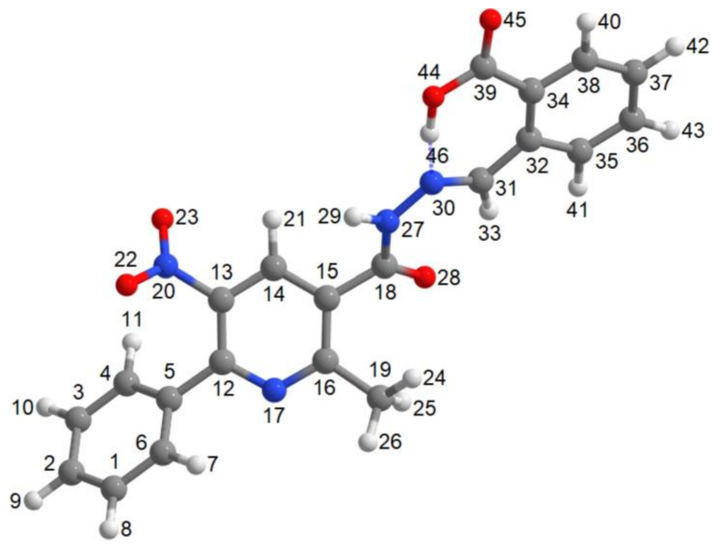

Figure S16: Optimized geometry of **7**, obtained using B3LYP-D3(BJ)/def2-TZVPP level of theory in DMSO with the help of the CPCM continuum solvation model

Table S.6: ^{1}H and ^{13}C NMR chemical shifts (TMS, ppm) for **7**, obtained using GIAO//B3LYP-D3(BJ)/def2-TZVPP level of theory in DMSO with the help of the CPCM continuum solvation model.

Atom	δ (*Z*-I)	δ (*Z*-II)	δ (*E*-I)	δ (*E*-II)	Atom	δ (*Z*-I)	δ (*Z*-II)	δ (*E*-I)	δ (*E*-II)
7 H	8.40	8.29	8.47	8.43	1 C	136.79	136.74	136.81	136.85
8 H	8.08	8.06	8.10	8.10	2 C	138.46	137.93	138.55	138.49
9 H	7.98	7.97	8.01	8.01	3 C	135.59	135.69	135.70	135.70
10 H	7.82	7.82	7.85	7.85	4 C	136.74	136.37	137.00	136.69
11 H	7.71	7.69	7.77	7.80	5 C	145.88	146.75	145.97	145.85
21 H	8.87	8.88	8.82	8.63	6 C	137.17	136.87	137.18	137.40
24 H	3.48	2.53	2.91	2.84	12 C	165.22	165.27	165.74	165.13
25 H	3.15	2.93	3.23	3.28	13 C	153.58	153.17	153.36	153.39
26 H	2.90	2.51	2.81	2.73	14 C	145.64	142.09	144.56	140.66
29 H	9.40	8.84	9.21	9.31	15 C	134.82	136.33	137.26	135.81
33 H	8.93	9.02	10.93	10.98	16 C	171.68	173.53	170.63	173.97
40 H	8.11	8.17	9.10	9.11	18 C	167.65	169.96	175.93	176.06
41 H	8.02	8.01	8.20	8.20	19 C	29.77	28.07	27.76	28.25
42 H	7.94	7.95	8.09	8.09	31 C	167.26	171.08	162.82	162.48
43 H	8.24	8.24	8.21	8.21	32 C	142.36	141.56	142.17	142.27
46 H	8.14	8.03	16.68	16.74	34 C	137.34	137.88	137.93	137.83
					35 C	137.94	137.82	146.30	146.25
					36 C	141.48	141.79	141.32	141.34
					37 C	137.90	138.15	139.50	139.44
					38 C	138.97	138.66	144.69	144.73
					39 C	180.47	181.31	177.12	177.04

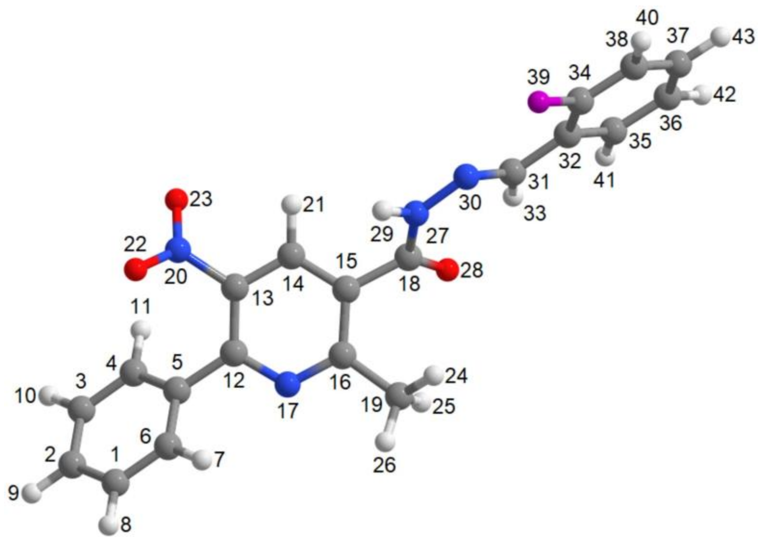

Figure S17: Optimized geometry of **8**, obtained using B3LYP-D3(BJ)/def2-TZVPP level of theory in DMSO with the help of the CPCM continuum solvation model

Table S.7: ^{1}H and ^{13}C NMR chemical shifts (TMS, ppm) for **8**, obtained using GIAO//B3LYP-D3(BJ)/def2-TZVPP level of theory in DMSO with the help of the CPCM continuum solvation model.

Atom	δ (*Z*-I)	δ (*Z*-II)	δ (*E*-I)	δ (*E*-II)	Atom	δ (*Z*-I)	δ (*Z*-II)	δ (*E*-I)	δ (*E*-II)
7 H	7.75	8.33	8.42	8.41	1 C	135.66	136.73	136.77	136.81
8 H	7.83	8.06	8.10	8.10	2 C	138.38	138.12	138.22	138.21
9 H	8.00	7.96	8.00	8.00	3 C	136.77	135.65	135.67	135.66
10 H	8.09	7.81	7.84	7.85	4 C	137.13	136.75	136.84	136.69
11 H	8.44	7.70	7.75	7.77	5 C	146.05	146.49	146.42	146.31
21 H	8.85	8.65	8.78	8.57	6 C	136.78	136.92	136.97	137.19
24 H	3.43	1.31	2.90	2.76	12 C	165.18	164.97	165.55	164.92
25 H	2.88	2.51	3.19	3.20	13 C	153.63	152.78	153.43	153.08
26 H	3.20	2.16	2.77	2.67	14 C	145.39	139.55	144.34	140.33
29 H	9.51	9.14	9.18	9.25	15 C	135.99	136.40	138.65	137.64
33 H	8.48	8.93	10.89	10.84	16 C	171.75	173.86	170.31	173.41
40 H	7.46	7.54	7.62	7.62	18 C	168.00	167.05	175.87	176.15
41 H	7.95	7.96	8.00	8.00	19 C	28.97	27.85	27.70	27.96
42 H	7.73	7.82	7.73	7.73	31 C	159.42	173.95	160.97	161.32
43 H	7.94	7.95	7.90	7.90	32 C	133.73	131.32	131.37	131.27
					34 C	171.57	169.74	173.55	173.50
					35 C	139.90	138.85	143.38	143.48
					36 C	130.88	132.55	131.72	131.74
					37 C	140.91	140.41	140.32	140.38
					38 C	122.36	123.38	123.87	123.91

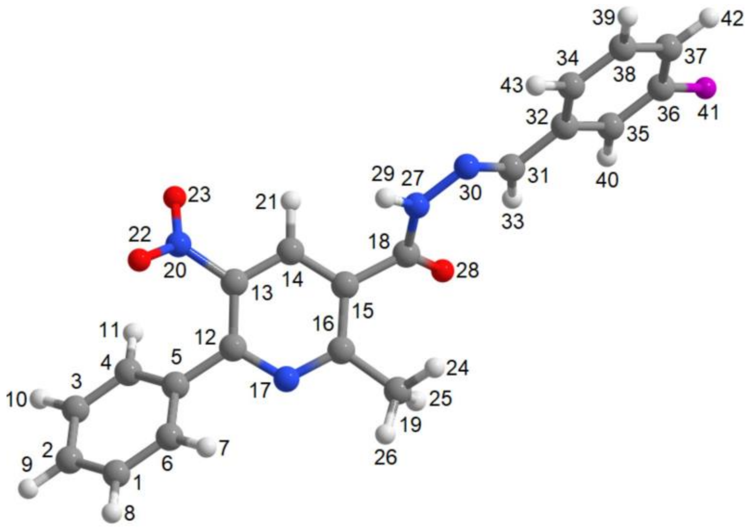

Figure S18: Optimized geometry of **9**, obtained using B3LYP-D3(BJ)/def2-TZVPP level of theory in DMSO with the help of the CPCM continuum solvation model

Table S.8: ^{1}H and ^{13}C NMR chemical shifts (TMS, ppm) for **9**, obtained using GIAO//B3LYP-D3(BJ)/def2-TZVPP level of theory in DMSO with the help of the CPCM continuum solvation model.

Atom	δ (Z-I)	δ (Z-II)	δ (E-I)	δ (E-II)	Atom	δ (Z-I)	δ (Z-II)	δ (E-I)	δ (E-II)
7 H	7.74	8.39	8.44	8.40	1 C	135.71	136.76	136.78	136.81
8 H	7.83	8.09	8.10	8.10	2 C	138.48	138.27	138.35	138.22
9 H	8.00	7.99	8.00	8.00	3 C	136.79	135.56	135.67	135.65
10 H	8.09	7.84	7.85	7.85	4 C	137.07	136.76	136.89	136.73
11 H	8.46	7.78	7.76	7.78	5 C	145.92	146.07	146.13	146.31
21 H	8.83	8.71	8.83	8.60	6 C	136.88	137.24	137.05	137.20
24 H	3.30	2.75	2.95	2.77	12 C	164.98	164.76	165.36	165.11
25 H	2.94	2.97	3.22	3.23	13 C	153.56	153.27	153.52	153.04
26 H	3.33	2.55	2.82	2.67	14 C	145.31	140.25	144.73	140.52
29 H	9.28	9.27	9.24	9.27	15 C	136.39	135.99	138.03	137.32
33 H	8.49	8.56	10.87	10.73	16 C	170.97	174.86	170.00	173.62
39 H	7.80	7.92	7.91	7.90	18 C	168.85	168.92	175.72	176.17
40 H	7.61	7.67	7.66	7.65	19 C	29.30	28.86	27.90	28.04
42 H	7.52	7.65	7.56	7.57	31 C	166.63	170.81	159.59	159.42
43 H	8.33	8.65	8.35	8.36	32 C	145.91	144.21	147.46	147.52
					34 C	130.10	131.29	125.96	126.09
					35 C	124.54	126.54	125.75	125.50
					36 C	175.50	175.38	175.70	175.78
					37 C	124.31	125.80	124.19	124.24
					38 C	137.99	138.69	138.47	138.35

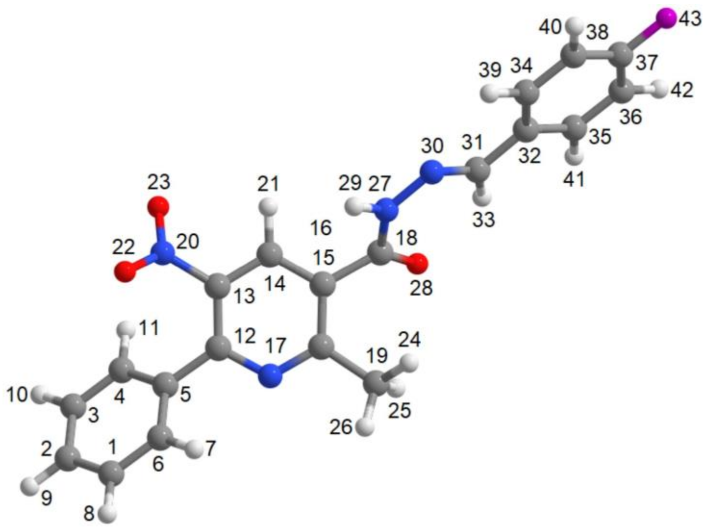

Figure S19: Optimized geometry of **10**, obtained using B3LYP-D3(BJ)/def2-TZVPP level of theory in DMSO with the help of the CPCM continuum solvation model

Table S.9: ^{1}H and ^{13}C NMR chemical shifts (TMS, ppm) for **10**, obtained using GIAO//B3LYP-D3(BJ)/def2-TZVPP level of theory in DMSO with the help of the CPCM continuum solvation model.

Atom	δ (*Z*-I)	δ (*Z*-II)	δ (*E*-I)	δ (*E*-II)	Atom	δ (*Z*-I)	δ (*Z*-II)	δ (*E*-I)	δ (*E*-II)
7 H	8.35	8.37	8.43	8.41	1 C	136.78	136.77	136.77	136.81
8 H	8.09	8.09	8.10	8.09	2 C	137.96	138.14	138.26	138.25
9 H	7.99	7.98	8.00	8.00	3 C	135.62	135.60	135.68	135.68
10 H	7.85	7.83	7.84	7.85	4 C	136.49	136.57	136.88	136.62
11 H	7.77	7.77	7.75	7.78	5 C	146.54	146.05	146.36	146.14
21 H	8.55	8.61	8.78	8.56	6 C	136.96	137.23	136.96	137.26
24 H	2.79	2.80	2.88	2.77	12 C	164.95	164.50	165.45	164.68
25 H	3.21	2.94	3.20	3.24	13 C	153.73	153.46	153.40	153.32
26 H	2.78	2.60	2.77	2.67	14 C	142.73	140.10	144.32	140.16
29 H	8.94	9.18	9.12	9.21	15 C	139.10	137.00	138.57	137.35
33 H	8.49	8.51	10.82	10.85	16 C	169.72	174.20	170.26	173.57
39 H	8.72	8.80	8.61	8.61	18 C	168.89	169.36	175.70	176.03
40 H	7.71	7.60	7.59	7.59	19 C	26.74	28.46	27.75	28.11
41 H	8.04	8.00	7.99	7.99	31 C	168.79	168.84	159.48	159.21
42 H	7.63	7.63	7.58	7.58	32 C	139.15	139.36	140.88	140.90
					34 C	138.97	138.50	133.18	133.17
					35 C	144.25	143.26	142.63	142.63
					36 C	121.81	122.04	122.02	122.03
					37 C	177.00	176.74	177.34	177.33
					38 C	122.34	122.50	122.96	122.95

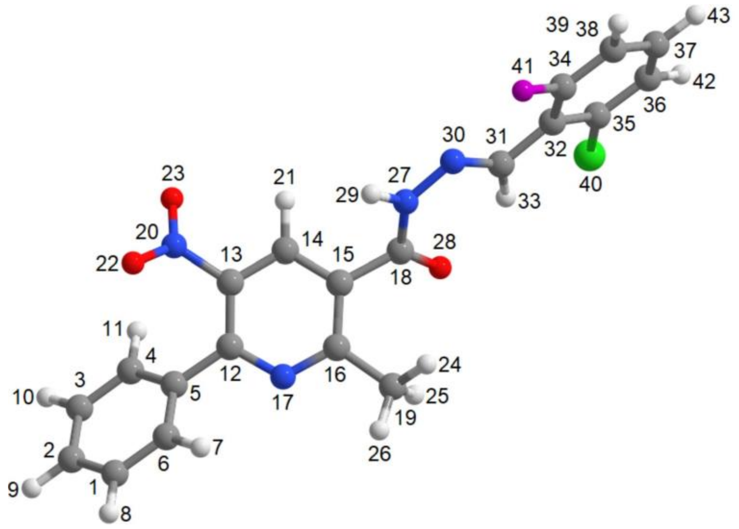

Figure S20: Optimized geometry of **11**, obtained using B3LYP-D3(BJ)/def2-TZVPP level of theory in DMSO with the help of the CPCM continuum solvation model

Table S.10: ^{1}H and ^{13}C NMR chemical shifts (TMS, ppm) for **11**, obtained using GIAO//B3LYP-D3(BJ)/def2-TZVPP level of theory in DMSO with the help of the CPCM continuum solvation model.

Atom	δ (*Z*-I)	δ (*Z*-II)	δ (*E*-I)	δ (*E*-II)	Atom	δ (*Z*-I)	δ (*Z*-II)	δ (*E*-I)	δ (*E*-II)
7 H	8.29	8.32	8.43	8.42	1 C	137.96	138.07	138.30	138.32
8 H	8.06	8.07	8.10	8.10	2 C	135.64	135.64	135.72	135.65
9 H	7.97	7.97	8.00	8.00	3 C	136.46	136.54	136.74	136.84
10 H	7.82	7.81	7.85	7.85	4 C	146.59	146.58	146.16	146.21
11 H	7.71	7.67	7.76	7.78	5 C	136.96	136.94	137.05	137.20
21 H	8.47	8.63	8.73	8.66	6 C	165.21	165.56	165.29	165.26
24 H	2.45	2.64	2.90	2.80	12 C	153.56	153.02	153.75	152.95
25 H	3.28	3.02	3.20	3.27	13 C	142.90	142.06	144.08	140.68
26 H	2.57	2.55	2.79	2.69	14 C	138.79	136.27	138.37	136.72
29 H	8.92	9.09	9.32	9.46	15 C	170.30	174.10	170.22	174.04
33 H	8.72	8.69	11.39	11.34	16 C	170.07	169.92	176.09	176.15
39 H	7.52	7.55	7.52	7.52	18 C	27.82	28.12	27.61	28.27
42 H	7.78	7.77	7.77	7.77	19 C	165.89	164.06	156.51	155.90
43 H	7.88	7.81	7.82	7.82	31 C	131.80	131.22	129.50	129.83
					32 C	169.27	170.05	174.37	173.90
					34 C	150.32	149.91	152.99	152.72
					35 C	133.94	134.00	133.72	133.63
					36 C	140.16	140.08	139.96	139.86
					37 C	122.11	121.72	122.72	122.64
					38 C	161.64	161.66	161.77	161.76

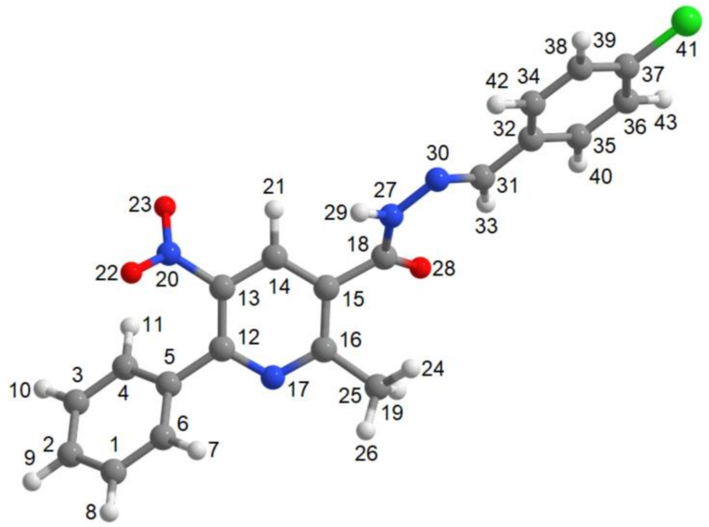

Figure S21: Optimized geometry of **12**, obtained using B3LYP-D3(BJ)/def2-TZVPP level of theory in DMSO with the help of the CPCM continuum solvation model

Table S.11: ^{1}H and ^{13}C NMR chemical shifts (TMS, ppm) for **12**, obtained using GIAO//B3LYP-D3(BJ)/def2-TZVPP level of theory in DMSO with the help of the CPCM continuum solvation model.

Atom	δ (*Z*-I)	δ (*Z*-II)	δ (*E*-I)	δ (*E*-II)	Atom	δ (*Z*-I)	δ (*Z*-II)	δ (*E*-I)	δ (*E*-II)
7 H	7.76	8.39	8.43	8.41	1 C	135.71	136.76	136.76	136.81
8 H	7.84	8.09	8.10	8.09	2 C	138.53	138.21	138.26	138.26
9 H	8.00	7.98	8.00	8.00	3 C	136.82	135.66	135.67	135.66
10 H	8.09	7.83	7.84	7.85	4 C	137.08	136.79	136.84	136.64
11 H	8.48	7.74	7.75	7.78	5 C	145.75	146.39	146.35	146.14
21 H	8.84	8.70	8.77	8.57	6 C	136.91	137.01	137.01	137.27
24 H	3.30	2.50	2.88	2.77	12 C	164.80	165.09	165.49	164.76
25 H	2.96	2.83	3.20	3.25	13 C	153.71	152.96	153.45	153.27
26 H	3.34	2.47	2.77	2.67	14 C	145.30	140.03	144.40	140.22
29 H	9.29	9.19	9.17	9.25	15 C	136.53	136.10	138.46	137.15
33 H	8.47	8.63	10.82	10.83	16 C	170.88	174.65	170.26	173.63
39 H	7.74	7.83	7.86	7.86	18 C	168.92	168.59	175.86	176.11
40 H	7.90	7.91	7.92	7.92	19 C	29.48	28.72	27.75	28.14
42 H	8.53	8.69	8.53	8.53	31 C	166.36	171.99	159.42	159.05
43 H	7.91	7.97	7.88	7.88	32 C	141.41	140.66	143.15	143.20
					34 C	136.67	135.95	131.95	131.95
					35 C	141.48	141.03	141.48	141.41
					36 C	136.23	136.92	136.43	136.41
					37 C	152.58	153.01	152.51	152.49
					38 C	135.79	136.62	136.73	136.74

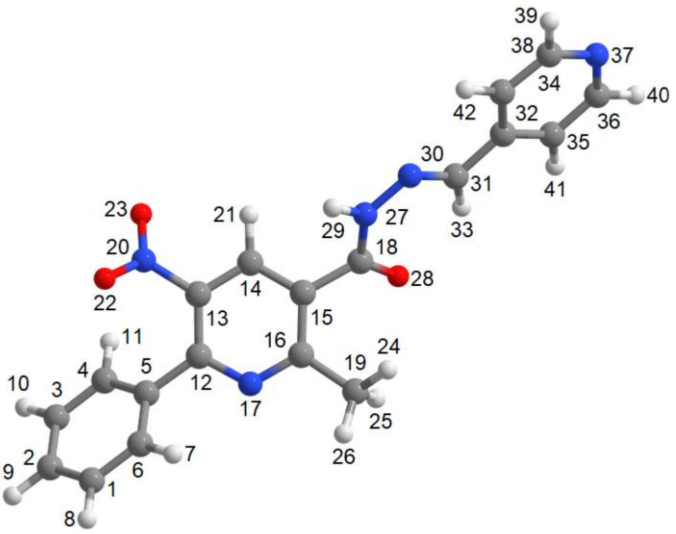

Figure S22: Optimized geometry of **13**, obtained using B3LYP-D3(BJ)/def2-TZVPP level of theory in DMSO with the help of the CPCM continuum solvation model

Table S.12: ^{1}H and ^{13}C NMR chemical shifts (TMS, ppm) for **13**, obtained using GIAO//B3LYP-D3(BJ)/def2-TZVPP level of theory in DMSO with the help of the CPCM continuum solvation model.

Atom	δ (*Z*-I)	δ (*Z*-II)	δ (*E*-I)	δ (*E*-II)	Atom	δ (*Z*-I)	δ (*Z*-II)	δ (*E*-I)	δ (*E*-II)
7 H	7.74	8.34	8.42	8.42	1 C	135.69	136.79	136.80	136.82
8 H	7.83	8.08	8.10	8.10	2 C	138.44	138.08	138.32	138.32
9 H	7.99	7.98	8.00	8.00	3 C	136.80	135.58	135.71	135.62
10 H	8.08	7.82	7.85	7.85	4 C	137.14	136.75	136.73	136.88
11 H	8.42	7.71	7.76	7.77	5 C	145.86	146.45	146.12	146.36
21 H	8.68	8.69	8.72	8.69	6 C	136.71	136.94	137.04	137.20
24 H	3.20	2.55	2.89	2.78	12 C	164.98	165.51	165.39	165.57
25 H	2.90	2.79	3.20	3.28	13 C	153.77	152.89	153.82	152.71
26 H	3.31	2.48	2.80	2.68	14 C	144.91	140.22	144.27	140.72
29 H	9.29	9.37	9.36	9.49	15 C	136.58	136.26	138.25	136.49
33 H	8.60	8.65	10.77	10.78	16 C	170.84	174.57	170.16	174.10
39 H	8.98	9.09	9.05	9.05	18 C	169.13	168.99	176.50	176.43
40 H	9.19	9.25	9.12	9.11	19 C	28.67	28.42	27.66	28.29
41 H	7.70	7.73	7.81	7.80	31 C	166.82	169.11	158.75	158.32
42 H	8.18	8.31	8.33	8.33	32 C	151.70	150.69	152.56	152.58
					34 C	126.18	126.78	123.50	123.51
					35 C	129.85	130.61	133.18	133.13
					36 C	160.16	160.40	160.01	160.00
					38 C	158.76	159.39	158.80	158.83

3.3 Cartesian coordinates of optimized geometries

Table S.13: Cartesian coordinates of optimized geometries for configurations and conformations of **4**, obtained using B3LYP-D3(BJ)/def2-TZVPP level of theory in DMSO with the help of the CPCM continuum solvation model.

Atom	*Z*-I			*Z*-II			*E*-I			*E*-II		
type	*x*	*y*	*z*	*x*	*y*	*z*	*x*	*y*	*z*	*x*	*y*	*z*
C	-4.11301	-0.36300	-0.42648	-3.30842	1.26046	1.23836	-4.43832	-0.24139	-0.57425	-4.36604	0.80151	0.37261
C	-4.51171	0.96441	-0.55593	-4.33321	0.39284	0.87208	-4.89789	1.04195	-0.29324	-5.06751	-0.35892	0.05897
C	-3.55944	1.97925	-0.53410	-4.04630	-0.73718	0.11152	-4.01299	2.00702	0.17929	-4.37767	-1.49626	-0.35185
C	-2.21415	1.66950	-0.38488	-2.74326	-0.99181	-0.29357	-2.67761	1.68741	0.38360	-2.99428	-1.46966	-0.46319
C	-1.81096	0.34069	-0.23463	-1.71180	-0.12171	0.06706	-2.21260	0.39957	0.10818	-2.28610	-0.30630	-0.15384
C	-2.77028	-0.67434	-0.26132	-2.00165	0.99971	0.84733	-3.09978	-0.55945	-0.38451	-2.98075	0.82570	0.27717
H	-2.46890	-1.71009	-0.17724	-1.20185	1.66818	1.13327	-2.73761	-1.55386	-0.60439	-2.43136	1.72309	0.52454
H	-4.84711	-1.15677	-0.45846	-3.52650	2.13933	1.83021	-5.12208	-0.99465	-0.94211	-4.89750	1.68734	0.69377
H	-5.55913	1.20602	-0.67824	-5.35023	0.59303	1.18189	-5.93962	1.29055	-0.44618	-6.14616	-0.37927	0.13999
H	-3.86453	3.01223	-0.63494	-4.83638	-1.42335	-0.16232	-4.36160	3.01019	0.38477	-4.91655	-2.40570	-0.58126
H	-1.47048	2.45370	-0.37192	-2.52780	-1.88116	-0.87096	-1.99323	2.44732	0.73713	-2.46433	-2.36162	-0.77025
C	-0.37321	0.02684	-0.10606	-0.31276	-0.40031	-0.31705	-0.78563	0.05457	0.28166	-0.80988	-0.27561	-0.21802
C	0.15755	-0.92362	0.77619	0.08613	-0.84912	-1.58037	-0.01275	0.42553	1.39142	-0.04800	-0.82015	-1.25825
C	1.48825	-1.27849	0.70050	1.39070	-1.25435	-1.80421	1.35140	0.21051	1.39883	1.32907	-0.91604	-1.14385
C	2.31579	-0.64201	-0.21440	2.31333	-1.13664	-0.78032	1.93711	-0.42570	0.31625	1.94639	-0.39829	-0.01951
C	1.76007	0.39433	-0.99195	1.88501	-0.58549	0.44513	1.10414	-0.87794	-0.72109	1.14453	0.23931	0.94785
N	0.45415	0.66901	-0.93180	0.60790	-0.26203	0.64336	-0.20448	-0.60818	-0.71953	-0.18286	0.26205	0.83348
C	3.74074	-1.11089	-0.21199	3.72383	-1.60139	-0.95680	3.41532	-0.67480	0.38527	3.42410	-0.54553	0.18010
C	2.55527	1.27916	-1.90438	2.82621	-0.32446	1.58020	1.60563	-1.69274	-1.87602	1.72251	0.92769	2.14512
N	-0.63505	-1.53133	1.84720	-0.81561	-0.85425	-2.73385	-0.60469	0.98773	2.60711	-0.64050	-1.25612	-2.52353
H	1.89114	-2.02614	1.36689	1.66490	-1.64247	-2.77528	1.94482	0.51045	2.25021	1.89972	-1.37769	-1.93690
O	-0.45796	-2.71933	2.07923	-1.55838	0.10372	-2.88454	-1.65491	0.50994	3.00849	-1.55867	-0.59674	-2.98723
O	-1.40224	-0.81272	2.46949	-0.74470	-1.80243	-3.50405	0.00629	1.88536	3.17143	-0.15598	-2.24011	-3.06716
H	3.49776	1.57807	-1.45145	3.75932	0.11408	1.22992	2.45850	-2.30981	-1.60093	2.65642	1.43363	1.90595
H	1.96934	2.16541	-2.13295	3.07772	-1.25805	2.08520	1.91655	-1.03874	-2.69460	1.93899	0.19884	2.92823
H	2.77835	0.77537	-2.84739	2.35413	0.34517	2.29425	0.80123	-2.32518	-2.24462	1.00308	1.64558	2.53134
N	4.37211	-1.14903	-1.44585	4.34962	-1.10161	-2.08479	4.10848	-0.22966	-0.69793	4.16526	-0.16411	-0.89816
O	4.28233	-1.48038	0.81301	4.28058	-2.32319	-0.15151	3.92507	-1.20402	1.36496	3.89305	-0.97811	1.22489
H	3.74442	-1.19486	-2.23691	3.73978	-0.78946	-2.82848	3.61001	0.29731	-1.39716	3.69076	0.28398	-1.66562
N	5.46938	-1.99442	-1.67223	5.51396	-1.68925	-2.60307	5.45456	-0.24110	-0.93145	5.52093	-0.16110	-1.06353
C	6.55439	-1.82394	-1.01156	6.58290	-1.64887	-1.89612	6.27679	-0.90797	-0.19570	6.31961	-0.74670	-0.23811
C	7.03212	-0.81703	-0.06088	6.95358	-1.01925	-0.62525	7.69846	-0.87818	-0.48092	7.75231	-0.71096	-0.46186
H	7.28092	-2.60588	-1.21932	7.39337	-2.21172	-2.35241	5.94748	-1.49994	0.64741	5.96148	-1.27564	0.63465
C	6.72898	0.55656	-0.09631	6.45544	0.19230	-0.10263	8.25396	-0.12400	-1.54083	8.34250	-0.03646	-1.55627
C	7.94292	-1.26854	0.90916	7.97280	-1.67362	0.09067	8.56063	-1.62555	0.33619	8.58968	-1.37186	0.45012
C	8.48895	-0.41594	1.84981	8.44579	-1.19685	1.29771	9.92735	-1.63629	0.12283	9.96438	-1.37483	0.29607
C	8.15817	0.93894	1.81189	7.92299	-0.00879	1.80854	10.45846	-0.88615	-0.92792	10.52967	-0.70380	-0.78977
C	7.29362	1.41957	0.84354	6.94517	0.67788	1.11119	9.63225	-0.13663	-1.75177	9.72873	-0.04036	-1.70690
H	7.04505	2.47074	0.78855	6.54353	1.60971	1.48542	10.03779	0.44722	-2.56713	10.16016	0.48153	-2.55052
H	9.17291	-0.79419	2.59653	9.21819	-1.73302	1.83083	10.57551	-2.21823	0.76290	10.59270	-1.89014	1.00892
O	5.94962	1.13003	-1.04687	5.54720	0.97928	-0.72901	7.48572	0.61927	-2.36484	7.60034	0.62090	-2.47186
H	8.20460	-2.31897	0.91537	8.38257	-2.58570	-0.32430	8.13061	-2.19978	1.14730	8.13402	-1.88568	1.28739
H	5.41597	0.46201	-1.50662	5.06708	0.47887	-1.41210	6.54741	0.51450	-2.07793	6.65175	0.52054	-2.21893
H	8.58433	1.62387	2.53282	8.28618	0.38811	2.74718	11.52584	-0.88502	-1.10570	11.60360	-0.69777	-0.92223

Table S.14: Cartesian coordinates of optimized geometries for configurations and conformations of **5**, obtained using B3LYP-D3(BJ)/def2-TZVPP level of theory in DMSO with the help of the CPCM continuum solvation model.

Atom	*Z*-I			*Z*-II			*E*-I			*E*-II		
type	*x*	*y*	*z*	*x*	*y*	*z*	*x*	*y*	*z*	*x*	*y*	*z*
C	-4.06540	-0.79788	-0.47116	-3.20241	1.60507	1.06967	-4.31998	-0.06590	-0.67775	-4.17376	1.07278	0.50672
C	-4.44362	0.52738	-0.66763	-4.27512	0.77277	0.76349	-4.73273	1.21882	-0.33715	-4.95117	-0.03146	0.16983
C	-3.47831	1.52990	-0.67519	-4.04701	-0.45029	0.13927	-3.82010	2.11961	0.20491	-4.33973	-1.19911	-0.27808
C	-2.14018	1.21001	-0.48758	-2.75448	-0.83422	-0.18985	-2.50407	1.73359	0.41912	-2.95837	-1.25874	-0.40248
C	-1.75807	-0.11564	-0.26978	-1.67446	0.00002	0.10872	-2.08585	0.44428	0.08231	-2.17401	-0.15189	-0.06996
C	-2.73025	-1.11861	-0.26788	-1.90726	1.21750	0.75265	-2.99995	-0.44926	-0.47926	-2.79086	1.01056	0.39739
H	-2.44418	-2.15297	-0.13032	-1.07092	1.85929	0.99087	-2.67345	-1.44453	-0.74598	-2.18224	1.86383	0.66170
H	-4.80973	-1.58265	-0.47976	-3.37424	2.55599	1.55599	-5.02576	-0.76907	-1.09925	-4.64434	1.98202	0.85618
H	-5.48546	0.77664	-0.81875	-5.28370	1.07306	1.01448	-5.75956	1.51908	-0.49812	-6.02815	0.01540	0.26117
H	-3.76739	2.56108	-0.82806	-4.87534	-1.10807	-0.08704	-4.13221	3.12413	0.45708	-4.93804	-2.06550	-0.52592
H	-1.38631	1.98450	-0.49582	-2.58688	-1.79478	-0.65845	-1.79696	2.44278	0.82906	-2.49087	-2.17534	-0.73736
C	-0.32744	-0.43945	-0.09531	-0.28703	-0.40516	-0.19831	-0.67705	0.03425	0.25944	-0.69949	-0.21000	-0.15093
C	0.17331	-1.34818	0.84629	0.11234	-1.02998	-1.38468	0.09293	0.31432	1.39835	0.01454	-0.78020	-1.21031
C	1.50296	-1.71414	0.82362	1.39844	-1.52210	-1.52343	1.45212	0.06885	1.40006	1.38711	-0.94554	-1.12702
C	2.35782	-1.12997	-0.10063	2.30609	-1.32394	-0.49803	2.03275	-0.51511	0.28587	2.04839	-0.47612	-0.00690
C	1.82952	-0.13395	-0.94674	1.88168	-0.60965	0.64096	1.19518	-0.89948	-0.77539	1.29785	0.17846	0.98905
N	0.52491	0.15199	-0.93408	0.62047	-0.19783	0.76276	-0.10506	-0.59334	-0.76906	-0.02816	0.27245	0.90036
C	3.78097	-1.59616	-0.03046	3.69178	-1.88071	-0.58142	3.50716	-0.78321	0.34726	3.52275	-0.68517	0.15916
C	2.65630	0.69755	-1.88095	2.80889	-0.26956	1.76663	1.67949	-1.68051	-1.96063	1.93519	0.80792	2.18899
N	-0.65081	-1.89794	1.92521	-0.76861	-1.14866	-2.54928	-0.49595	0.80343	2.64599	-0.62673	-1.17637	-2.46493
H	1.88265	-2.42823	1.53869	1.67223	-2.04002	-2.43207	2.04621	0.30496	2.27058	1.92145	-1.41877	-1.93867
O	-0.48537	-3.07339	2.22137	-1.44996	-0.18201	-2.85418	-1.58747	0.36545	2.97756	-1.47711	-0.43594	-2.93506
O	-1.43189	-1.14676	2.48879	-0.74396	-2.20224	-3.17121	0.15772	1.59850	3.30822	-0.24788	-2.21490	-2.99032
H	3.56763	1.04722	-1.40039	3.78573	0.04028	1.39913	2.51897	-2.32543	-1.70972	2.88729	1.27388	1.93950
H	2.06942	1.55305	-2.20389	2.96243	-1.14236	2.40325	2.00248	-1.00321	-2.75534	2.13307	0.05080	2.94999
H	2.94131	0.13133	-2.76992	2.37318	0.52590	2.36539	0.86160	-2.28176	-2.35122	1.26157	1.55112	2.60827
N	4.44981	-1.70624	-1.24225	4.37412	-1.54044	-1.73631	4.20645	-0.31957	-0.72623	4.26412	-0.22957	-0.89079
O	4.29348	-1.90295	1.02900	4.18508	-2.54614	0.30928	4.01373	-1.34054	1.31227	3.98979	-1.21687	1.15705
H	3.84626	-1.81356	-2.04644	3.80696	-1.24011	-2.51753	3.71447	0.21764	-1.42232	3.79110	0.27801	-1.62166
N	5.55815	-2.55448	-1.37676	5.49186	-2.25647	-2.18277	5.54664	-0.36224	-0.97164	5.61738	-0.22549	-1.05674
C	6.61582	-2.33357	-0.68979	6.54792	-2.28592	-1.45823	6.37374	-0.99805	-0.21592	6.41691	-0.84402	-0.25819
C	7.03902	-1.25408	0.20963	6.96195	-1.63259	-0.21129	7.79052	-0.99330	-0.53527	7.85018	-0.78564	-0.48500
H	7.35970	-3.11574	-0.81835	7.30754	-2.94321	-1.87339	6.05676	-1.54155	0.66346	6.06390	-1.41194	0.59147
C	6.74121	0.11066	0.04636	6.56957	-0.35950	0.24774	8.32609	-0.30658	-1.64911	8.43221	-0.05597	-1.54713
C	7.89089	-1.63218	1.25942	7.91153	-2.34680	0.54066	8.65985	-1.69815	0.30982	8.68734	-1.48125	0.39928
C	8.37561	-0.69289	2.14445	8.40311	-1.84203	1.72436	10.01496	-1.71779	0.05217	10.05596	-1.45088	0.22959
C	8.06531	0.65546	1.99611	7.99359	-0.59728	2.19243	10.54618	-1.04344	-1.04354	10.63296	-0.73435	-0.81522
C	7.25645	1.04628	0.94480	7.08735	0.13332	1.44751	9.69992	-0.34248	-1.88723	9.81896	-0.04118	-1.69632
O	6.01691	0.60464	-0.98520	5.74458	0.47570	-0.42330	7.54785	0.39099	-2.49826	7.68642	0.63592	-2.42919
H	7.01560	2.08998	0.79728	6.76737	1.11099	1.78030	10.09538	0.18730	-2.74302	10.25011	0.52121	-2.51321
H	8.15189	-2.67539	1.36928	8.24438	-3.30910	0.17753	8.25131	-2.22100	1.16341	8.24351	-2.03750	1.21327
Br	9.51015	-1.24611	3.58436	9.67882	-2.85550	2.73039	11.18631	-2.68374	1.22138	11.18073	-2.40677	1.45159
H	8.45520	1.39156	2.68385	8.38331	-0.19839	3.11765	11.60871	-1.06275	-1.23817	11.70577	-0.71481	-0.94156
H	5.49866	-0.09666	-1.41205	5.25152	0.00488	-1.11761	6.61423	0.31152	-2.18668	6.73790	0.51768	-2.18117

Table S.15: Cartesian coordinates of optimized geometries for configurations and conformations of **6**, obtained using B3LYP-D3(BJ)/def2-TZVPP level of theory in DMSO with the help of the CPCM continuum solvation model.

Atom	*Z*-I			*Z*-II			*E*-I			*E*-II		
type	*x*	*y*	*z*	*x*	*y*	*z*	*x*	*y*	*z*	*x*	*y*	*z*
C	-3.21560	-0.62929	-0.06170	-2.56379	1.82983	0.73414	-4.30653	0.01463	-0.68856	-4.13310	1.04213	0.58686
C	-3.60615	0.61741	-0.54225	-3.61262	0.91593	0.69318	-4.71258	1.27159	-0.24990	-4.92456	-0.06227	0.28511
C	-2.64235	1.54461	-0.92798	-3.36975	-0.40152	0.31485	-3.79394	2.12482	0.35526	-4.32991	-1.24199	-0.15363
C	-1.29348	1.22799	-0.83397	-2.08699	-0.80107	-0.03475	-2.47872	1.71822	0.53408	-2.95163	-1.31374	-0.30380
C	-0.89606	-0.01417	-0.33464	-1.03194	0.11311	0.00103	-2.06720	0.45607	0.09961	-2.15321	-0.20677	-0.00746
C	-1.86782	-0.94279	0.04616	-1.27693	1.42868	0.39958	-2.98762	-0.38845	-0.52436	-2.75309	0.96818	0.45085
H	-1.57383	-1.91980	0.40622	-0.45859	2.13421	0.43365	-2.66646	-1.36208	-0.86678	-2.13368	1.82169	0.68793
H	-3.96048	-1.35930	0.22513	-2.74738	2.85470	1.02780	-5.01689	-0.65161	-1.15956	-4.59033	1.96073	0.92956
H	-4.65691	0.86284	-0.62025	-4.61391	1.22733	0.95946	-5.73881	1.58721	-0.38331	-5.99919	-0.00608	0.39670
H	-2.94133	2.51431	-1.30289	-4.17869	-1.11941	0.29558	-4.10046	3.10847	0.68485	-4.93883	-2.10842	-0.37406
H	-0.54145	1.94379	-1.13445	-1.90422	-1.82971	-0.31627	-1.76776	2.39202	0.99407	-2.49719	-2.23965	-0.63089
C	0.54025	-0.35150	-0.26380	0.34847	-0.30462	-0.32577	-0.66027	0.02633	0.24177	-0.68061	-0.27516	-0.11694
C	1.14012	-1.03750	0.80202	0.69642	-1.09376	-1.42615	0.11857	0.22822	1.39103	0.00942	-0.87107	-1.17774
C	2.43692	-1.49213	0.69419	1.99027	-1.56624	-1.57299	1.47417	-0.03289	1.37004	1.38406	-1.03430	-1.12285
C	3.17573	-1.20135	-0.44510	2.94919	-1.19631	-0.64750	2.04699	-0.55294	0.21960	2.07166	-0.53924	-0.03004
C	2.57133	-0.38566	-1.42322	2.56572	-0.33748	0.40155	1.19929	-0.85807	-0.85996	1.34226	0.13288	0.97001
N	1.28792	-0.02500	-1.31724	1.30134	0.06334	0.53846	-0.09828	-0.54028	-0.82640	0.01432	0.22677	0.90965
C	4.57942	-1.73514	-0.44905	4.34261	-1.74498	-0.74412	3.52195	-0.83133	0.27427	3.55285	-0.73716	0.10564
C	3.27626	0.15640	-2.63156	3.54176	0.17197	1.41708	1.66235	-1.56643	-2.09857	2.00503	0.78181	2.14595
N	0.46485	-1.23984	2.08485	-0.25250	-1.42062	-2.49213	-0.45784	0.65076	2.66862	-0.66056	-1.29892	-2.40684
H	2.89184	-2.04237	1.50366	2.23203	-2.20392	-2.41160	2.07502	0.13972	2.25056	1.89981	-1.52449	-1.93650
O	0.64376	-2.30445	2.66060	-1.01867	-0.54826	-2.87200	-1.54707	0.19719	2.98667	-1.51541	-0.56648	-2.88150
O	-0.20886	-0.32165	2.52803	-0.19983	-2.54725	-2.96782	0.20351	1.40834	3.36627	-0.29982	-2.35553	-2.90859
H	4.32267	0.37422	-2.43768	4.47983	0.47293	0.95452	2.49170	-2.24102	-1.89830	2.95339	1.24027	1.87024
H	2.76830	1.06106	-2.95653	3.77688	-0.61217	2.13861	1.99036	-0.84392	-2.85073	2.21600	0.03691	2.91549
H	3.23052	-0.55993	-3.45616	3.10818	1.01638	1.94628	0.83065	-2.12517	-2.52174	1.34169	1.53382	2.56601
N	5.11946	-2.00357	-1.67332	4.93916	-1.53846	-1.94970	4.22062	-0.39794	-0.80778	4.26602	-0.28915	-0.96280
O	5.17800	-1.92703	0.60099	4.87267	-2.32772	0.19246	4.02090	-1.37102	1.25674	4.03684	-1.25392	1.10623
H	4.47155	-2.15836	-2.43076	4.35076	-1.22988	-2.70908	3.71242	0.08309	-1.53127	3.75829	0.18219	-1.69404
N	6.27481	-2.76216	-1.84852	6.02968	-2.28516	-2.41007	5.56338	-0.43726	-1.08082	5.61996	-0.24281	-1.17498
C	7.38088	-2.37904	-1.32165	7.14894	-2.21524	-1.78763	6.39448	-0.95487	-0.24907	6.43756	-0.79099	-0.34969
C	7.80511	-1.17859	-0.60571	7.65209	-1.38957	-0.69403	7.82533	-0.97595	-0.53167	7.87912	-0.70897	-0.55630
H	8.17643	-3.10406	-1.47703	7.88489	-2.90383	-2.19654	6.07557	-1.38389	0.69167	6.09886	-1.32152	0.53051
C	7.16106	0.06334	-0.66422	7.10453	-0.16467	-0.29345	8.38488	-0.48733	-1.71350	8.46412	-0.03689	-1.63089
C	8.98552	-1.27658	0.14434	8.81804	-1.83541	-0.05564	8.67089	-1.50812	0.45511	8.70679	-1.32819	0.39398
C	9.49522	-0.19551	0.84019	9.39702	-1.12241	0.97792	10.04599	-1.55006	0.27775	10.08868	-1.28049	0.28346
C	8.83999	1.04332	0.76656	8.82669	0.09495	1.38121	10.59599	-1.05209	-0.91867	10.66440	-0.59665	-0.80417
C	7.67834	1.15801	0.00413	7.69046	0.56800	0.72275	9.75834	-0.52956	-1.89896	9.84462	0.01384	-1.74790
H	6.26630	0.18832	-1.25062	6.22859	0.22798	-0.78383	7.74906	-0.07532	-2.48338	7.84159	0.44755	-2.36876
O	9.38286	2.08718	1.44082	9.41374	0.75689	2.40726	11.94738	-1.10814	-1.05684	12.02154	-0.56576	-0.87531
H	7.18385	2.11955	-0.05883	7.26163	1.51439	1.02883	10.19998	-0.15238	-2.81369	10.30592	0.53534	-2.57816
H	9.52120	-2.21590	0.19594	9.28311	-2.76477	-0.35863	8.23201	-1.88387	1.36747	8.24919	-1.84448	1.22480
O	10.66799	-0.32417	1.54285	10.49473	-1.62895	1.62830	10.93181	-2.04070	1.18400	10.95843	-1.84876	1.15950
C	10.49887	-0.42650	2.96598	11.74067	-1.00906	1.26964	10.41560	-2.55317	2.41162	10.41566	-2.55497	2.27419
H	11.49687	-0.49906	3.39065	12.50980	-1.50027	1.86015	11.27883	-2.88730	2.97982	11.26874	-2.92869	2.83291
H	9.92619	-1.32277	3.21403	11.72565	0.05695	1.49801	9.74422	-3.39552	2.23317	9.79700	-3.39158	1.94369
H	9.99322	0.45446	3.36253	11.94177	-1.15606	0.20639	9.88681	-1.77710	2.96862	9.82318	-1.89170	2.90754
H	8.84460	2.87811	1.31363	8.94244	1.57987	2.58762	12.20144	-0.73322	-1.90872	12.29417	-0.06996	-1.65689

Table S.16: Cartesian coordinates of optimized geometries for configurations and conformations of **7**, obtained using B3LYP-D3(BJ)/def2-TZVPP level of theory in DMSO with the help of the CPCM continuum solvation model.

Atom	*Z*-I			*Z*-II			*E*-I			*E*-II		
type	*x*	*y*	*z*	*x*	*y*	*z*	*x*	*y*	*z*	*x*	*y*	*z*
C	-4.29184	-0.02854	0.33296	-4.17625	0.01301	0.18484	-4.33268	-0.07812	-0.69944	-4.22356	0.97055	0.63135
C	-4.86465	0.84067	1.25694	-4.56115	-1.17455	-0.43063	-4.78230	1.15758	-0.24338	-4.99046	-0.08629	0.14963
C	-4.05341	1.54630	2.14114	-3.59356	-2.04582	-0.92287	-3.90065	2.02239	0.39922	-4.36695	-1.18639	-0.43230
C	-2.67648	1.37279	2.11275	-2.24696	-1.72570	-0.81306	-2.57874	1.64851	0.59760	-2.98397	-1.22443	-0.54521
C	-2.09686	0.49790	1.19069	-1.85622	-0.53373	-0.19893	-2.12334	0.40777	0.14538	-2.20989	-0.16407	-0.06845
C	-2.91335	-0.19220	0.29206	-2.82939	0.32768	0.31058	-3.00695	-0.44824	-0.51552	-2.83914	0.92828	0.53286
H	-2.46289	-0.86266	-0.42612	-2.52536	1.24658	0.79204	-2.65130	-1.40458	-0.87197	-2.23896	1.74447	0.90950
H	-4.91844	-0.57790	-0.35683	-4.92473	0.69293	0.56926	-5.01412	-0.75287	-1.19997	-4.70352	1.82704	1.08552
H	-5.93817	0.97201	1.28470	-5.61021	-1.42260	-0.52209	-5.81366	1.44820	-0.39264	-6.06875	-0.05544	0.23176
H	-4.49257	2.23492	2.85037	-3.88701	-2.97697	-1.38870	-4.24127	2.99002	0.74223	-4.95710	-2.01766	-0.79384
H	-2.05203	1.93627	2.79332	-1.49998	-2.41380	-1.18694	-1.89683	2.33251	1.08543	-2.50754	-2.09115	-0.98380
C	-0.63004	0.33499	1.11729	-0.42518	-0.20046	-0.03299	-0.70783	0.01433	0.30205	-0.73469	-0.20305	-0.14068
C	0.21365	0.21431	2.22980	0.53482	-0.32080	-1.04255	0.05379	0.22819	1.46119	-0.00764	-0.64168	-1.25250
C	1.58258	0.24936	2.07150	1.88045	-0.15680	-0.76127	1.41790	0.01265	1.45036	1.36522	-0.79970	-1.17872
C	2.12875	0.34255	0.79776	2.25365	0.19378	0.52415	2.01294	-0.47793	0.29906	2.01613	-0.45183	-0.00795
C	1.23628	0.34232	-0.29506	1.24242	0.39809	1.48352	1.18551	-0.80657	-0.78926	1.25116	0.07079	1.05349
N	-0.08638	0.36360	-0.09971	-0.03983	0.18159	1.18898	-0.12017	-0.52880	-0.76494	-0.07531	0.15931	0.96490
C	3.63074	0.39530	0.76426	3.69928	0.32803	0.88709	3.49105	-0.72067	0.34346	3.49270	-0.65243	0.13406
C	1.65511	0.31185	-1.73413	1.53433	0.87005	2.87456	1.68446	-1.49543	-2.02446	1.86683	0.55939	2.32800
N	-0.29713	-0.00454	3.58532	0.17325	-0.57144	-2.43863	-0.54837	0.61748	2.73769	-0.63522	-0.89933	-2.55043
H	2.23348	0.17979	2.92943	2.61516	-0.29798	-1.54064	2.00529	0.19945	2.33729	1.90656	-1.17649	-2.03491
O	-1.24221	-0.76577	3.72682	-0.80440	0.00502	-2.89154	-1.62650	0.12707	3.03777	-1.47226	-0.10501	-2.95079
O	0.27342	0.56660	4.50474	0.89420	-1.31947	-3.08532	0.08118	1.38777	3.45036	-0.25686	-1.88010	-3.17661
H	2.52221	-0.32205	-1.89604	2.33093	1.61218	2.88821	2.54041	-2.13498	-1.82092	2.81419	1.06543	2.14953
H	1.89602	1.31905	-2.08654	1.86173	0.03275	3.49344	1.98578	-0.75884	-2.77373	2.06770	-0.28169	2.99401
H	0.82276	-0.04849	-2.33322	0.63145	1.29058	3.31012	0.87930	-2.08902	-2.45150	1.17609	1.23585	2.82511
N	4.19694	0.99239	-0.33287	4.42337	1.10069	0.01498	4.17741	-0.19422	-0.71364	4.21652	-0.14311	-0.90817
O	4.29307	-0.02769	1.69921	4.17963	-0.21846	1.86638	4.01580	-1.31537	1.27367	3.98450	-1.22312	1.09593
H	3.59492	1.58550	-0.88245	3.90232	1.77257	-0.52986	3.67999	0.37987	-1.37629	3.73150	0.37352	-1.62500
N	5.49555	1.48360	-0.35273	5.70686	1.56166	0.30858	5.50685	-0.25479	-0.99107	5.55826	-0.17551	-1.12008
C	6.48565	0.71821	-0.09548	6.62884	0.69702	0.49110	6.34881	-0.92615	-0.29863	6.39157	-0.74923	-0.33530
C	6.58295	-0.72952	0.17404	6.60575	-0.77382	0.35941	7.78259	-0.98428	-0.59761	7.84058	-0.75947	-0.55763
H	7.43422	1.24819	-0.07024	7.57869	1.12741	0.79683	6.03287	-1.47530	0.57591	6.05449	-1.24204	0.56441
C	5.91640	-1.73882	-0.54242	6.03503	-1.46973	-0.72141	8.50926	-0.48930	-1.71821	8.60378	-0.30793	-1.67250
C	7.42223	-1.09695	1.22898	7.18433	-1.50220	1.40034	8.49726	-1.64534	0.41592	8.52948	-1.31043	0.53653
C	7.55589	-2.42284	1.61209	7.15430	-2.88918	1.40652	9.87190	-1.80357	0.37922	9.90991	-1.40263	0.58099
C	6.85905	-3.41393	0.92989	6.54846	-3.57310	0.35884	10.57916	-1.30348	-0.70343	10.65106	-0.94657	-0.49833
C	6.04875	-3.06893	-0.14143	5.99578	-2.86377	-0.69798	9.89516	-0.66861	-1.72941	9.99491	-0.41953	-1.60052
C	5.09170	-1.51023	-1.76483	5.48287	-0.81786	-1.94633	7.99484	0.20992	-2.96370	8.12725	0.28051	-2.98827
H	5.52692	-3.83360	-0.69873	5.54749	-3.38679	-1.53067	10.43941	-0.29357	-2.58185	10.56659	-0.07821	-2.44913
H	7.95186	-0.32340	1.76913	7.63507	-0.96764	2.22591	7.94353	-2.03676	1.25835	7.95035	-1.66776	1.37697
H	6.95770	-4.45110	1.21906	6.51785	-4.65394	0.35616	11.65388	-1.40971	-0.75837	11.73091	-1.00342	-0.49118
H	8.20269	-2.68061	2.43958	7.60010	-3.43285	2.22823	10.37934	-2.31231	1.18731	10.39557	-1.82683	1.44903
O	5.53714	-0.62370	-2.67182	6.17551	0.19833	-2.48774	6.75327	0.65382	-3.02258	6.86896	0.64541	-3.14607
O	4.07285	-2.12034	-1.99383	4.47091	-1.19270	-2.49290	8.73300	0.38613	-3.91719	8.91073	0.43981	-3.90806
H	6.39989	-0.25893	-2.43357	7.02131	0.34475	-2.04273	6.18709	0.38623	-2.22473	6.27264	0.40244	-2.36157

Table S.17: Cartesian coordinates of optimized geometries for configurations and conformations of **8**, obtained using B3LYP-D3(BJ)/def2-TZVPP level of theory in DMSO with the help of the CPCM continuum solvation model.

Atom	*Z*-I			*Z*-II			*E*-I			*E*-II		
type	*x*	*y*	*z*	*x*	*y*	*z*	*x*	*y*	*z*	*x*	*y*	*z*
C	-4.28293	-0.24638	-0.26130	-3.29514	1.26516	1.32586	-4.33021	-0.10665	-0.63570	-4.21882	0.78771	0.50596
C	-4.62487	1.09792	-0.37884	-4.35956	0.46866	0.91357	-4.75790	1.16933	-0.28126	-4.95508	-0.34541	0.17256
C	-3.62902	2.07015	-0.36845	-4.12123	-0.64963	0.11958	-3.85236	2.07994	0.25622	-4.30259	-1.48374	-0.29250
C	-2.29620	1.70098	-0.24279	-2.82691	-0.96304	-0.27225	-2.52821	1.71223	0.45227	-2.92191	-1.48520	-0.43756
C	-1.94968	0.35526	-0.10445	-1.75553	-0.16486	0.13552	-2.09509	0.43146	0.10182	-2.17886	-0.34921	-0.10881
C	-2.95268	-0.61686	-0.11935	-1.99774	0.94472	0.94791	-3.00236	-0.47182	-0.45513	-2.83623	0.78348	0.37597
H	-2.69446	-1.66490	-0.04376	-1.16744	1.55673	1.27120	-2.66443	-1.46028	-0.73266	-2.25977	1.65945	0.63806
H	-5.05166	-1.00698	-0.28464	-3.47519	2.13440	1.94419	-5.03037	-0.81731	-1.05406	-4.72120	1.67426	0.86921
H	-5.66252	1.38580	-0.48316	-5.36946	0.71440	1.21378	-5.79100	1.45519	-0.42778	-6.03158	-0.34383	0.28026
H	-3.89032	3.11590	-0.46019	-4.94294	-1.28095	-0.19086	-4.17640	3.07788	0.51931	-4.86818	-2.37266	-0.53746
H	-1.51847	2.45157	-0.23921	-2.64965	-1.84268	-0.87716	-1.82684	2.42874	0.85923	-2.42161	-2.37930	-0.78541
C	-0.52539	-0.02462	-0.00298	-0.36671	-0.51702	-0.22778	-0.67836	0.04084	0.26025	-0.70413	-0.34786	-0.20971
C	-0.02379	-1.00117	0.86942	0.03787	-0.93774	-1.49937	0.10102	0.32342	1.39224	0.01841	-0.87886	-1.28386
C	1.28580	-1.42094	0.76574	1.31289	-1.43996	-1.70162	1.46328	0.09432	1.37707	1.39698	-0.99773	-1.21514
C	2.12466	-0.82583	-0.16639	2.20665	-1.44627	-0.64620	2.03929	-0.47505	0.25287	2.05345	-0.51895	-0.09623
C	1.60461	0.23696	-0.93176	1.78497	-0.90756	0.58632	1.19452	-0.86133	-0.80184	1.29072	0.10110	0.91229
N	0.31410	0.57584	-0.84627	0.53165	-0.48858	0.76166	-0.10953	-0.57173	-0.77889	-0.03885	0.14893	0.83849
C	3.53609	-1.33546	-0.18397	3.57322	-2.04391	-0.78602	3.51766	-0.73189	0.29970	3.53502	-0.69344	0.06009
C	2.41737	1.07529	-1.87288	2.70130	-0.77312	1.76331	1.67549	-1.62745	-1.99808	1.91734	0.74259	2.11143
N	-0.82254	-1.56805	1.95783	-0.82412	-0.81859	-2.67556	-0.47942	0.79652	2.65021	-0.62086	-1.27905	-2.53821
H	1.66808	-2.18505	1.42555	1.58957	-1.80518	-2.68064	2.06360	0.33023	2.24327	1.93952	-1.44282	-2.03703
O	-0.68499	-2.75937	2.20137	-1.51591	0.18323	-2.78152	-1.56368	0.34634	2.98940	-1.51143	-0.57025	-2.98221
O	-1.55601	-0.81702	2.58300	-0.77553	-1.71392	-3.50899	0.17391	1.59160	3.31282	-0.20098	-2.28700	-3.09137
H	3.44056	1.20037	-1.53237	3.71628	-0.52345	1.46190	2.52065	-2.26919	-1.75826	2.85971	1.22742	1.86173
H	1.94119	2.04707	-1.97877	2.74894	-1.71711	2.30912	1.99018	-0.94104	-2.78824	2.12972	-0.01117	2.87195
H	2.44818	0.61526	-2.86428	2.31765	-0.00726	2.43325	0.85850	-2.22958	-2.38918	1.22949	1.47171	2.53269
N	4.15751	-1.31776	-1.39956	4.28055	-1.59606	-1.85784	4.20414	-0.25323	-0.77549	4.26218	-0.20481	-0.98502
O	4.05822	-1.77750	0.82988	3.99973	-2.85972	0.02049	4.02973	-1.29440	1.25972	4.01033	-1.23070	1.05205
H	3.56760	-1.26017	-2.21529	3.79589	-1.10260	-2.59163	3.69211	0.29031	-1.45170	3.76572	0.31221	-1.69323
N	5.36158	-1.90223	-1.70653	5.45951	-2.21006	-2.29402	5.54418	-0.26173	-1.05312	5.61753	-0.14437	-1.17204
C	6.39866	-1.81004	-0.95785	6.45537	-2.15542	-1.49715	6.35851	-0.93046	-0.32019	6.40899	-0.79677	-0.40042
C	6.73822	-1.08073	0.26936	6.59113	-1.42218	-0.22641	7.79575	-0.96866	-0.57352	7.86155	-0.76233	-0.54444
H	7.22350	-2.40956	-1.33446	7.32090	-2.72559	-1.82367	6.02377	-1.50773	0.53153	6.04196	-1.41603	0.40714
C	6.26972	0.17997	0.63162	6.29626	-0.06719	-0.10424	8.48429	-0.27144	-1.57314	8.59203	0.00732	-1.45724
C	7.67966	-1.67023	1.12681	7.07470	-2.07966	0.90897	8.57299	-1.77806	0.27132	8.60870	-1.57292	0.32636
C	8.06994	-1.05736	2.30665	7.19874	-1.41682	2.12094	9.94651	-1.88673	0.12282	9.99315	-1.61488	0.28279
C	7.55411	0.19211	2.64217	6.86509	-0.06834	2.21232	10.58914	-1.17806	-0.88730	10.67802	-0.83421	-0.64294
C	6.65414	0.82300	1.79187	6.42004	0.61852	1.08896	9.85214	-0.36355	-1.74066	9.97166	-0.01643	-1.51846
F	5.42504	0.83321	-0.19874	5.90247	0.62615	-1.19787	7.81004	0.53586	-2.42008	7.94926	0.82061	-2.32249
H	6.25720	1.80421	2.01155	6.17525	1.67080	1.12534	10.32448	0.20072	-2.53275	10.47654	0.60313	-2.24660
H	8.08888	-2.63460	0.85528	7.32952	-3.12832	0.83338	8.06948	-2.32743	1.05623	8.07297	-2.17724	1.04698
H	8.78090	-1.54565	2.95840	7.55682	-1.94865	2.99130	10.51326	-2.51983	0.79112	10.53567	-2.25127	0.96784
H	7.86087	0.68385	3.55511	6.96100	0.45531	3.15351	11.66033	-1.25359	-1.01440	11.75824	-0.85632	-0.68713

Table S.18: Cartesian coordinates of optimized geometries for configurations and conformations of **9**, obtained using B3LYP-D3(BJ)/def2-TZVPP level of theory in DMSO with the help of the CPCM continuum solvation model.

Atom	*Z*-I			*Z*-II			*E*-I			*E*-II		
type	*x*	*y*	*z*	*x*	*y*	*z*	*x*	*y*	*z*	*x*	*y*	*z*
C	-4.24397	-0.31009	-0.38879	-3.59565	1.07918	1.19510	-4.31609	0.03055	-0.71948	-4.21220	0.83365	0.43650
C	-4.59711	1.03328	-0.48058	-4.60736	0.29004	0.65576	-4.72083	1.28807	-0.28147	-4.95733	-0.28582	0.07791
C	-3.61430	2.01543	-0.39915	-4.28393	-0.77942	-0.17465	-3.80435	2.13603	0.33431	-4.31103	-1.43317	-0.37358
C	-2.28350	1.65744	-0.22774	-2.95641	-1.05047	-0.47661	-2.49272	1.72355	0.52427	-2.92715	-1.45691	-0.48036
C	-1.92646	0.31209	-0.11481	-1.93742	-0.25903	0.05829	-2.08237	0.46093	0.09016	-2.17512	-0.33449	-0.12634
C	-2.91644	-0.66997	-0.20200	-2.26614	0.80092	0.90622	-3.00043	-0.37826	-0.54436	-2.82670	0.80701	0.34515
H	-2.64948	-1.71711	-0.14785	-1.47670	1.40848	1.32561	-2.68017	-1.35235	-0.88613	-2.24337	1.67242	0.62646
H	-5.00155	-1.07817	-0.46727	-3.84204	1.91058	1.84191	-5.02481	-0.63155	-1.19858	-4.70996	1.72708	0.78908
H	-5.63290	1.31275	-0.62018	-5.64271	0.50365	0.88567	-5.74437	1.60837	-0.42367	-6.03628	-0.26663	0.15513
H	-3.88419	3.06056	-0.47091	-5.06535	-1.40523	-0.58434	-4.10994	3.12006	0.66341	-4.88423	-2.31146	-0.63836
H	-1.51617	2.41634	-0.16870	-2.71359	-1.89264	-1.11100	-1.78313	2.39321	0.99227	-2.43168	-2.35723	-0.81894
C	-0.50372	-0.05671	0.03623	-0.51558	-0.55622	-0.21524	-0.67889	0.02485	0.24341	-0.69852	-0.35561	-0.18976
C	-0.03202	-1.06410	0.88974	-0.00261	-0.90000	-1.47029	0.09122	0.22157	1.39931	0.04246	-0.89001	-1.24964
C	1.28232	-1.47545	0.82162	1.31434	-1.30495	-1.60596	1.44606	-0.04308	1.38843	1.41726	-1.02561	-1.14932
C	2.15640	-0.83808	-0.04811	2.13851	-1.30076	-0.49401	2.02528	-0.56239	0.24108	2.05390	-0.56077	-0.01287
C	1.66322	0.25793	-0.78436	1.59413	-0.87250	0.73405	1.18570	-0.86415	-0.84543	1.27483	0.05990	0.98286
N	0.36777	0.58516	-0.74161	0.30820	-0.53656	0.83805	-0.11080	-0.54245	-0.82136	-0.05209	0.12434	0.87786
C	3.56714	-1.35096	-0.03384	3.56288	-1.75716	-0.58924	3.49815	-0.84357	0.30323	3.53148	-0.74246	0.16698
C	2.51018	1.14475	-1.64804	2.40774	-0.76829	1.98723	1.65704	-1.57194	-2.08115	1.87969	0.68521	2.20139
N	-0.86967	-1.67271	1.92450	-0.78998	-0.79184	-2.70006	-0.49424	0.64204	2.67356	-0.57013	-1.27266	-2.52277
H	1.63988	-2.26651	1.46323	1.67743	-1.60299	-2.57953	2.04068	0.12665	2.27378	1.97269	-1.47194	-1.96180
O	-0.74105	-2.87258	2.12670	-1.50852	0.18643	-2.83779	-1.58718	0.19054	2.98138	-1.44499	-0.55297	-2.98016
O	-1.62487	-0.94479	2.55129	-0.65255	-1.67189	-3.53952	0.16269	1.39721	3.37789	-0.14364	-2.27738	-3.07677
H	3.50960	1.28045	-1.24471	3.40187	-0.37375	1.78997	2.48692	-2.24460	-1.87699	2.82674	1.17240	1.97481
H	2.02104	2.11074	-1.74629	2.53734	-1.75605	2.43248	1.98634	-0.84854	-2.83179	2.07894	-0.07842	2.95556
H	2.61004	0.72291	-2.65143	1.89162	-0.12942	2.69936	0.82904	-2.13299	-2.50843	1.18455	1.40917	2.61937
N	4.24505	-1.26644	-1.21854	4.25610	-1.20689	-1.62544	4.20466	-0.40315	-0.77539	4.27293	-0.27241	-0.87729
O	4.04815	-1.83813	0.97887	4.04983	-2.54118	0.21354	3.99510	-1.38692	1.28205	3.99593	-1.26865	1.16944
H	3.68469	-1.19298	-2.05431	3.73056	-0.75282	-2.35686	3.70313	0.08678	-1.49796	3.78720	0.22066	-1.60953
N	5.42451	-1.95200	-1.47998	5.46408	-1.72700	-2.09636	5.54414	-0.44697	-1.03459	5.62516	-0.25244	-1.06381
C	6.44741	-1.79095	-0.72793	6.48559	-1.71500	-1.32630	6.36804	-0.98417	-0.21212	6.42332	-0.84660	-0.25533
C	6.73992	-0.87899	0.38681	6.74870	-1.12028	-0.00916	7.80186	-0.99502	-0.50691	7.86918	-0.79240	-0.48230
H	7.27194	-2.44493	-1.00086	7.34394	-2.21642	-1.76660	6.05300	-1.43356	0.71995	6.07839	-1.39897	0.60862
C	6.18546	0.39914	0.51383	6.03832	-0.04104	0.53175	8.34682	-0.46713	-1.68473	8.44951	-0.05065	-1.51984
C	7.69333	-1.30628	1.31689	7.85196	-1.62823	0.68740	8.65394	-1.55227	0.45243	8.69347	-1.51501	0.38714
C	8.02913	-0.47015	2.36256	8.18899	-1.07527	1.90611	10.01398	-1.56180	0.21276	10.06094	-1.47935	0.19369
C	7.48838	0.79343	2.51099	7.49557	-0.01631	2.46107	10.57333	-1.04689	-0.94087	10.65419	-0.75535	-0.82257
C	6.56515	1.22483	1.56156	6.41648	0.50282	1.75017	9.71646	-0.49684	-1.89423	9.82546	-0.03552	-1.68292
H	6.13825	2.21472	1.64640	5.86829	1.34173	2.15612	10.13151	-0.08816	-2.80548	10.26750	0.54095	-2.48410
H	8.16033	-2.27800	1.23083	8.43791	-2.44223	0.28258	8.26282	-1.96624	1.37202	8.27559	-2.09445	1.19945
F	8.93641	-0.90541	3.27253	9.25229	-1.58270	2.57691	10.83144	-2.09799	1.15398	10.85309	-2.18662	1.03905
H	7.78763	1.42033	3.33915	7.79800	0.39207	3.41507	11.64389	-1.07548	-1.08715	11.72926	-0.75409	-0.93426
H	5.47355	0.75569	-0.21285	5.20534	0.38446	-0.00350	7.69151	-0.03590	-2.42701	7.81518	0.51200	-2.18864

Table S.19: Cartesian coordinates of optimized geometries for configurations and conformations of **10**, obtained using B3LYP-D3(BJ)/def2-TZVPP level of theory in DMSO with the help of the CPCM continuum solvation model.

Atom	*Z*-I			*Z*-II			*E*-I			*E*-II		
type	*x*	*y*	*z*	*x*	*y*	*z*	*x*	*y*	*z*	*x*	*y*	*z*
C	-3.78469	-3.16285	-1.99013	-3.42351	1.73033	0.95213	-4.31044	-0.04089	-0.71732	-4.18222	1.02023	0.54217
C	-4.51378	-2.19567	-1.30439	-4.49016	0.86906	0.71241	-4.72753	1.23300	-0.34309	-4.95047	-0.07609	0.16113
C	-3.85075	-1.24398	-0.53470	-4.25380	-0.39385	0.17680	-3.82093	2.11766	0.23451	-4.32832	-1.22410	-0.32125
C	-2.46588	-1.26717	-0.43910	-2.95916	-0.78905	-0.13115	-2.50679	1.72601	0.45016	-2.94560	-1.27157	-0.43585
C	-1.73004	-2.23797	-1.12181	-1.88568	0.07372	0.10127	-2.08420	0.44721	0.07996	-2.17027	-0.17236	-0.05977
C	-2.39834	-3.17852	-1.90809	-2.12626	1.33224	0.65687	-2.99227	-0.42964	-0.51663	-2.79813	0.96965	0.44217
H	-1.82725	-3.92529	-2.44164	-1.29497	1.99731	0.84369	-2.66256	-1.41646	-0.80948	-2.19696	1.81694	0.74061
H	-4.29567	-3.90411	-2.58993	-3.60154	2.71229	1.36972	-5.01140	-0.73144	-1.16681	-4.66101	1.91422	0.91880
H	-5.59337	-2.18022	-1.37312	-5.50019	1.17784	0.94675	-5.75287	1.53755	-0.50559	-6.02846	-0.03841	0.24471
H	-4.41155	-0.48101	-0.01171	-5.07714	-1.07340	0.00242	-4.13623	3.11412	0.51333	-4.91936	-2.08487	-0.60381
H	-1.95689	-0.51661	0.15122	-2.78420	-1.77839	-0.53258	-1.80439	2.42322	0.88777	-2.47013	-2.17373	-0.79756
C	-0.25226	-2.25014	-1.06717	-0.49537	-0.34011	-0.18468	-0.67668	0.03292	0.25837	-0.69477	-0.21850	-0.13275
C	0.49714	-2.09041	0.10669	-0.09127	-1.01540	-1.34070	0.08564	0.28662	1.40855	0.02904	-0.74847	-1.20603
C	1.87193	-1.95460	0.05127	1.20230	-1.49444	-1.46136	1.44535	0.04256	1.41304	1.40212	-0.90710	-1.12046
C	2.49917	-2.03183	-1.18007	2.10903	-1.24170	-0.44797	2.03499	-0.51441	0.28978	2.05584	-0.46915	0.01715
C	1.71615	-2.28920	-2.31557	1.67416	-0.49168	0.66235	1.20361	-0.87585	-0.78461	1.29436	0.14644	1.02974
N	0.38544	-2.36907	-2.23361	0.40908	-0.08512	0.76761	-0.09698	-0.57072	-0.77996	-0.03229	0.23288	0.93758
C	3.99561	-1.92631	-1.21692	3.50033	-1.79657	-0.51598	3.51084	-0.78320	0.35511	3.53285	-0.67189	0.18226
C	2.31402	-2.48989	-3.67561	2.59333	-0.10936	1.78090	1.69476	-1.63110	-1.98381	1.91806	0.74224	2.25385
N	-0.11284	-2.12276	1.43736	-0.97582	-1.20610	-2.49226	-0.51220	0.74461	2.66359	-0.60285	-1.10649	-2.47694
H	2.44270	-1.81836	0.95857	1.48599	-2.04390	-2.34793	2.03299	0.25792	2.29326	1.94278	-1.35157	-1.94409
O	-0.99911	-2.93745	1.64533	-1.67853	-0.26954	-2.83989	-1.60620	0.29868	2.97633	-1.44703	-0.34978	-2.93228
O	0.32562	-1.34948	2.27884	-0.93560	-2.28878	-3.06160	0.13603	1.52337	3.35028	-0.22234	-2.13010	-3.02960
H	3.31920	-2.90406	-3.62364	3.57762	0.17107	1.41271	2.52791	-2.28675	-1.73990	2.86875	1.22232	2.02745
H	2.37122	-1.53592	-4.20632	2.73085	-0.95447	2.45774	2.03017	-0.93757	-2.75912	2.11496	-0.03704	2.99237
H	1.67875	-3.15384	-4.25771	2.16114	0.71679	2.33956	0.87700	-2.21750	-2.39657	1.23529	1.46683	2.69077
N	4.45744	-0.83228	-1.87748	4.16968	-1.48788	-1.66234	4.21478	-0.30414	-0.70708	4.27059	-0.20524	-0.86393
O	4.70944	-2.73964	-0.64726	3.97171	-2.46660	0.39213	4.00657	-1.35648	1.31791	3.99731	-1.21169	1.17859
H	3.81517	-0.35508	-2.49238	3.62995	-1.11002	-2.42657	3.71599	0.23757	-1.39436	3.78220	0.28267	-1.59755
N	5.78628	-0.65324	-2.26414	5.28853	-2.18686	-2.12070	5.55596	-0.33915	-0.97249	5.62290	-0.20474	-1.06801
C	6.70030	-0.62050	-1.36631	6.35078	-2.21816	-1.40640	6.37100	-0.97878	-0.21627	6.42362	-0.77393	-0.24323
C	6.68250	-0.61290	0.09864	6.75615	-1.53890	-0.17304	7.80116	-0.99719	-0.52022	7.86694	-0.74679	-0.47599
H	7.69636	-0.55198	-1.79668	7.11658	-2.86135	-1.83337	6.04783	-1.52071	0.66285	6.07815	-1.28634	0.64503
C	5.58316	-0.23409	0.88243	6.23137	-0.31400	0.25869	8.35407	-0.32300	-1.61789	8.44899	-0.08948	-1.56884
C	7.88221	-0.94617	0.74049	7.78796	-2.12988	0.56552	8.64897	-1.71729	0.32844	8.69731	-1.40197	0.43992
C	7.98295	-0.94887	2.12412	8.25793	-1.54873	1.73423	10.01774	-1.77180	0.09719	10.07697	-1.41080	0.27828
C	6.87037	-0.58011	2.85727	7.70312	-0.34609	2.13104	10.52113	-1.09364	-0.99669	10.60930	-0.75224	-0.81384
C	5.67485	-0.20849	2.26399	6.70861	0.29279	1.40868	9.71618	-0.36772	-1.86196	9.82253	-0.08895	-1.74369
H	4.65627	0.06172	0.41844	5.45933	0.17810	-0.31163	7.70948	0.23824	-2.27863	7.81788	0.42346	-2.28004
H	4.83557	0.08835	2.87686	6.31700	1.24129	1.74797	10.15667	0.14748	-2.70408	10.28548	0.41428	-2.58101
H	8.74568	-1.21392	0.14583	8.22138	-3.06059	0.22364	8.23197	-2.23937	1.17983	8.25798	-1.90966	1.28881
H	8.90132	-1.22007	2.62515	9.04230	-2.00691	2.31973	10.68027	-2.32506	0.74763	10.72624	-1.91424	0.98054
F	6.95247	-0.57310	4.20740	8.15512	0.23568	3.26558	11.85414	-1.13603	-1.23557	11.95330	-0.75134	-0.98479

Table S.20: Cartesian coordinates of optimized geometries for configurations and conformations of **11**, obtained using B3LYP-D3(BJ)/def2-TZVPP level of theory in DMSO with the help of the CPCM continuum solvation model.

Atom	*Z*-I			*Z*-II			*E*-I			*E*-II		
type	*x*	*y*	*z*	*x*	*y*	*z*	*x*	*y*	*z*	*x*	*y*	*z*
C	-3.64905	-0.93910	-0.59230	-3.80660	-0.05125	0.31579	-4.34288	-0.10484	-0.61637	-4.22180	0.99647	0.50926
C	-4.38054	0.13779	-0.10017	-4.20133	-1.23064	-0.30895	-4.76222	1.18277	-0.29499	-4.96467	-0.13790	0.19568
C	-3.73471	1.15040	0.60365	-3.24535	-2.06794	-0.87724	-3.84943	2.10252	0.21358	-4.31774	-1.29399	-0.23222
C	-2.36620	1.08053	0.82740	-1.90184	-1.72136	-0.83425	-2.52626	1.73312	0.41338	-2.93574	-1.31179	-0.36052
C	-1.62869	-0.00039	0.33971	-1.50112	-0.53680	-0.21187	-2.10162	0.44078	0.09686	-2.18594	-0.17436	-0.05206
C	-2.27755	-1.00339	-0.38292	-2.46144	0.28956	0.37452	-3.01652	-0.47230	-0.43119	-2.83779	0.97633	0.39609
H	-1.70453	-1.83550	-0.76736	-2.14950	1.20185	0.86329	-2.68504	-1.46986	-0.68266	-2.25626	1.85351	0.64245
H	-4.14600	-1.72785	-1.14112	-4.54598	0.60194	0.75954	-5.04860	-0.82275	-1.01236	-4.72003	1.89662	0.84360
H	-5.44789	0.19093	-0.26871	-5.24864	-1.49899	-0.34876	-5.79434	1.47029	-0.44493	-6.04234	-0.12350	0.28984
H	-4.29573	1.99745	0.97504	-3.54561	-2.99308	-1.35062	-4.16666	3.10913	0.45060	-4.88888	-2.18351	-0.46158
H	-1.86979	1.87858	1.36375	-1.16317	-2.38333	-1.26705	-1.81965	2.45824	0.79521	-2.43951	-2.21874	-0.67982
C	-0.16338	-0.06913	0.52748	-0.07014	-0.17756	-0.11700	-0.68698	0.04560	0.26107	-0.71070	-0.18899	-0.13825
C	0.49584	0.19444	1.73513	0.83817	-0.26771	-1.17745	0.09881	0.36406	1.37850	0.01645	-0.75815	-1.18958
C	1.87636	0.27220	1.77914	2.19336	-0.08937	-0.95943	1.45840	0.12143	1.36991	1.39219	-0.88931	-1.10261
C	2.59752	0.03302	0.62305	2.62805	0.24716	0.31043	2.02447	-0.49602	0.26620	2.04495	-0.38347	0.00735
C	1.89544	-0.33285	-0.53803	1.66440	0.42434	1.32228	1.17387	-0.91044	-0.77314	1.27842	0.27389	0.99011
N	0.56073	-0.35224	-0.55838	0.37190	0.19399	1.08902	-0.12721	-0.60845	-0.75753	-0.05007	0.33181	0.90118
C	4.09566	0.09537	0.70839	4.09260	0.36769	0.59067	3.50070	-0.76184	0.31232	3.52378	-0.56103	0.17473
C	2.59090	-0.73814	-1.80103	2.02060	0.87768	2.70392	1.64516	-1.71930	-1.94497	1.89588	0.94619	2.17710
N	-0.21891	0.34298	3.00425	0.41200	-0.50320	-2.55775	-0.47486	0.89830	2.61485	-0.61283	-1.18682	-2.43957
H	2.37744	0.49146	2.71068	2.89007	-0.20356	-1.77700	2.06356	0.38641	2.22457	1.93420	-1.36873	-1.90538
O	-1.17445	-0.38785	3.21768	-0.60400	0.05128	-2.94885	-1.54325	0.44402	2.99561	-1.49886	-0.48773	-2.90730
O	0.20356	1.17460	3.79701	1.12032	-1.21780	-3.25501	0.16783	1.74817	3.21665	-0.18919	-2.20743	-2.96596
H	3.44404	-1.38061	-1.58990	2.81580	1.62103	2.69061	2.49991	-2.34397	-1.69470	2.83443	1.43418	1.91941
H	2.96438	0.13684	-2.33525	2.37854	0.03177	3.29363	1.93939	-1.06060	-2.76612	2.11499	0.21067	2.95315
H	1.89292	-1.26418	-2.44706	1.13832	1.28854	3.18837	0.82855	-2.34416	-2.29967	1.19940	1.67704	2.58052
N	4.66563	1.01637	-0.12470	4.77129	1.09722	-0.34687	4.18243	-0.31286	-0.78039	4.24964	-0.19800	-0.92385
O	4.72201	-0.60230	1.49041	4.61865	-0.16783	1.55388	4.01733	-1.30357	1.28103	4.00481	-0.99760	1.21172
H	4.05952	1.73950	-0.48448	4.24193	1.76894	-0.88221	3.66264	0.19464	-1.47835	3.75198	0.21576	-1.69582
N	5.97801	1.46051	0.01096	6.11245	1.43406	-0.26188	5.51446	-0.34724	-1.07546	5.59427	-0.23391	-1.15364
C	6.91740	0.60349	-0.08485	6.96474	0.49424	-0.11845	6.33945	-0.94127	-0.29148	6.39376	-0.72761	-0.27969
C	6.84355	-0.81124	-0.49161	6.75902	-0.96439	-0.15388	7.77393	-0.98045	-0.56458	7.83939	-0.76224	-0.49523
H	7.90799	0.99914	0.11764	7.98665	0.83359	0.01616	6.01146	-1.43818	0.60754	6.03761	-1.14446	0.64914
C	6.27513	-1.18936	-1.70615	6.10045	-1.60030	-1.20508	8.42529	-0.22709	-1.55410	8.52783	0.02059	-1.43428
C	7.41667	-1.83408	0.26947	7.27612	-1.79828	0.84315	8.62048	-1.79145	0.21876	8.65479	-1.58908	0.30254
C	7.38208	-3.16028	-0.13128	7.11690	-3.17428	0.81470	9.99281	-1.84189	0.03444	10.03392	-1.63117	0.17539
C	6.77678	-3.48650	-1.33931	6.43156	-3.75388	-0.24649	10.57610	-1.06778	-0.95937	10.65525	-0.82862	-0.77178
C	6.22315	-2.49803	-2.14317	5.92028	-2.96758	-1.27088	9.78917	-0.25297	-1.76215	9.89956	0.00577	-1.58448
H	5.76655	-2.72565	-3.09556	5.39942	-3.39658	-2.11472	10.21556	0.36204	-2.54167	10.35621	0.64531	-2.32623
Cl	8.17588	-1.42900	1.78607	8.11376	-1.07639	2.19248	7.95174	-2.80341	1.48075	7.93211	-2.63890	1.50202
F	5.77607	-0.22540	-2.50894	5.63687	-0.84820	-2.22658	7.70088	0.58478	-2.34728	7.83416	0.85819	-2.22884
H	7.82070	-3.92394	0.49354	7.51888	-3.77984	1.61322	10.59428	-2.48172	0.66243	10.61093	-2.28693	0.80986
H	6.74501	-4.51840	-1.65968	6.30114	-4.82649	-0.27718	11.64632	-1.10024	-1.10749	11.73087	-0.85393	-0.87562

Table S.21: Cartesian coordinates of optimized geometries for configurations and conformations of **12**, obtained using B3LYP-D3(BJ)/def2-TZVPP level of theory in DMSO with the help of the CPCM continuum solvation model.

Atom	*Z*-I			*Z*-II			*E*-I			*E*-II		
type	*x*	*y*	*z*	*x*	*y*	*z*	*x*	*y*	*z*	*x*	*y*	*z*
C	-4.08469	-0.64509	-0.59093	-3.21678	1.48222	1.36750	-4.32644	-0.06780	-0.70812	-4.19395	1.04388	0.53478
C	-4.45504	0.69663	-0.57641	-4.28958	0.69447	0.96020	-4.75537	1.20312	-0.33745	-4.96520	-0.05437	0.16586
C	-3.48410	1.68230	-0.42444	-4.06330	-0.43168	0.17380	-3.85647	2.09912	0.23468	-4.34656	-1.20939	-0.30430
C	-2.14795	1.32955	-0.28784	-2.77249	-0.76157	-0.21577	-2.53801	1.72160	0.44894	-2.96413	-1.26192	-0.41901
C	-1.77366	-0.01582	-0.28126	-1.69278	0.02803	0.18666	-2.10367	0.44565	0.08250	-2.18578	-0.16077	-0.05495
C	-2.75163	-1.00095	-0.43920	-1.92290	1.14546	0.99177	-3.00398	-0.44254	-0.50902	-2.81002	0.98842	0.43488
H	-2.47086	-2.04557	-0.46693	-1.08603	1.75090	1.31045	-2.66476	-1.42694	-0.79908	-2.20627	1.83711	0.72393
H	-4.83296	-1.41461	-0.72486	-3.38755	2.35758	1.97978	-5.02140	-0.76694	-1.15363	-4.67016	1.94318	0.90188
H	-5.49509	0.97270	-0.68831	-5.29685	0.95335	1.25819	-5.78417	1.49659	-0.49841	-6.04303	-0.01277	0.24940
H	-3.76761	2.72623	-0.41333	-4.89178	-1.05602	-0.13273	-4.18121	3.09337	0.51046	-4.94015	-2.07147	-0.57723
H	-1.38971	2.09116	-0.17303	-2.60441	-1.64677	-0.81511	-1.84151	2.42737	0.88215	-2.49082	-2.16894	-0.77126
C	-0.34646	-0.37709	-0.16108	-0.30808	-0.33779	-0.17770	-0.69228	0.04474	0.25998	-0.71059	-0.21152	-0.12785
C	0.13648	-1.44646	0.60586	0.08912	-0.77034	-1.44737	0.06898	0.30613	1.40928	0.01142	-0.75482	-1.19562
C	1.45640	-1.83280	0.50969	1.36223	-1.27526	-1.65155	1.42984	0.06905	1.41430	1.38403	-0.91611	-1.10869
C	2.32442	-1.11104	-0.29838	2.26435	-1.27175	-0.60247	2.02226	-0.48710	0.29200	2.03913	-0.46827	0.02428
C	1.81791	0.03536	-0.94302	1.84707	-0.72770	0.62967	1.19359	-0.85301	-0.78274	1.27973	0.16007	1.03062
N	0.51742	0.33939	-0.87979	0.59459	-0.30685	0.80767	-0.10886	-0.55612	-0.77788	-0.04664	0.24950	0.93747
C	3.74388	-1.59927	-0.31340	3.63365	-1.85950	-0.75764	3.49754	-0.75423	0.36062	3.51528	-0.67164	0.19010
C	2.65505	1.00531	-1.72304	2.76467	-0.58736	1.80507	1.68983	-1.60431	-1.98221	1.90491	0.76732	2.24831
N	-0.69562	-2.14778	1.58511	-0.77756	-0.65924	-2.62124	-0.53032	0.76361	2.66412	-0.62107	-1.12443	-2.46294
H	1.82371	-2.66976	1.08405	1.63060	-1.65012	-2.62906	2.01637	0.28846	2.29419	1.92284	-1.37094	-1.92783
O	-0.56363	-3.36053	1.67831	-1.46209	0.34648	-2.73612	-1.62054	0.31101	2.98009	-1.46676	-0.37299	-2.92408
O	-1.44748	-1.47993	2.27921	-0.73918	-1.56475	-3.44395	0.11376	1.54861	3.34748	-0.23954	-2.15208	-3.00733
H	3.64788	1.12692	-1.29924	3.77497	-0.32328	1.50170	2.52312	-2.25944	-1.73711	2.85270	1.24992	2.01475
H	2.14839	1.96686	-1.74715	2.82753	-1.53375	2.34519	2.02740	-0.90851	-2.75454	2.10897	-0.00568	2.99139
H	2.77271	0.66856	-2.75623	2.37206	0.16980	2.47953	0.87417	-2.19052	-2.39921	1.22038	1.49183	2.68234
N	4.43324	-1.40249	-1.47702	4.30643	-1.43964	-1.86436	4.20516	-0.27559	-0.70027	4.25249	-0.22447	-0.86616
O	4.22540	-2.15735	0.66238	4.09558	-2.64091	0.06335	3.99110	-1.32783	1.32391	3.98212	-1.19412	1.19398
H	3.88153	-1.27160	-2.31147	3.78667	-0.97366	-2.59225	3.70994	0.26785	-1.38888	3.76417	0.25113	-1.60787
N	5.63175	-2.03956	-1.77747	5.44329	-2.08858	-2.35713	5.54488	-0.32127	-0.96452	5.60302	-0.23074	-1.07067
C	6.64731	-1.90566	-1.00813	6.49150	-2.10974	-1.62351	6.35427	-0.96523	-0.20596	6.40419	-0.78568	-0.23678
C	6.92064	-1.07474	0.16853	6.82268	-1.45062	-0.35440	7.78325	-0.99809	-0.51274	7.84650	-0.76224	-0.47549
H	7.48813	-2.51791	-1.32472	7.29301	-2.70978	-2.04730	6.02758	-1.50076	0.67554	6.06102	-1.28190	0.66131
C	6.27236	0.13417	0.44663	6.29049	-0.21797	0.04178	8.34270	-0.33324	-1.61181	8.42532	-0.12099	-1.57852
C	7.94889	-1.49415	1.01947	7.78628	-2.05981	0.45525	8.62514	-1.72562	0.33432	8.68119	-1.40360	0.44511
C	8.29519	-0.76044	2.14459	8.17853	-1.48753	1.65663	9.99148	-1.79685	0.09877	10.05934	-1.41408	0.27721
C	7.62428	0.42715	2.40028	7.62451	-0.27083	2.02956	10.51601	-1.12979	-0.99858	10.60313	-0.77227	-0.82574
C	6.62463	0.89001	1.55194	6.69320	0.37683	1.22573	9.70306	-0.39578	-1.85817	9.79758	-0.12340	-1.75768
H	6.12596	1.82605	1.75742	6.28619	1.33170	1.52492	10.13368	0.11711	-2.70608	10.24317	0.37213	-2.60817
H	8.48001	-2.41168	0.80207	8.22827	-2.99775	0.14528	8.20587	-2.24183	1.18832	8.24728	-1.89997	1.30358
Cl	8.05154	1.36705	3.80931	8.11428	0.47010	3.53316	12.23414	-1.20967	-1.31115	12.33651	-0.77448	-1.05266
H	5.50092	0.50299	-0.20933	5.56751	0.28914	-0.57783	7.70524	0.23498	-2.27373	7.79341	0.38281	-2.29565
H	9.07861	-1.10034	2.80613	8.91029	-1.97193	2.28651	10.63683	-2.36062	0.75650	10.69902	-1.91149	0.99165

Table S.22: Cartesian coordinates of optimized geometries for configurations and conformations of **13**, obtained using B3LYP-D3(BJ)/def2-TZVPP level of theory in DMSO with the help of the CPCM continuum solvation model.

Atom	*Z*-I			*Z*-II			*E*-I			*E*-II		
type	*x*	*y*	*z*	*x*	*y*	*z*	*x*	*y*	*z*	*x*	*y*	*z*
C	-4.32754	-0.44197	-0.20874	-3.73000	1.39491	0.74261	-4.44453	-0.18288	-0.60838	-4.36415	0.79293	0.40234
C	-4.71215	0.84101	-0.58736	-4.70008	0.43108	0.48393	-4.88997	1.09569	-0.28634	-5.07034	-0.35693	0.06166
C	-3.75078	1.83612	-0.73888	-4.32597	-0.80956	-0.02472	-3.99683	2.03270	0.22569	-4.38543	-1.48682	-0.37702
C	-2.41069	1.55035	-0.51401	-2.99017	-1.08026	-0.28756	-2.66734	1.68909	0.42883	-3.00205	-1.46311	-0.48879
C	-2.02157	0.26940	-0.11609	-2.01324	-0.11504	-0.03400	-2.21655	0.40558	0.11215	-2.28901	-0.31027	-0.15213
C	-2.99009	-0.72639	0.03069	-2.39111	1.12072	0.49466	-3.11162	-0.52456	-0.41999	-2.97887	0.81392	0.30638
H	-2.69987	-1.73087	0.30909	-1.63437	1.86524	0.69842	-2.76011	-1.51524	-0.67145	-2.42573	1.70283	0.57497
H	-5.06831	-1.22330	-0.10485	-4.01558	2.35984	1.13969	-5.13497	-0.91402	-1.00708	-4.89186	1.67279	0.74535
H	-5.75547	1.06267	-0.76827	-5.74213	0.64318	0.68278	-5.92727	1.36282	-0.43826	-6.14898	-0.37502	0.14328
H	-4.04454	2.83464	-1.03373	-5.07383	-1.56804	-0.21315	-4.33454	3.03251	0.46320	-4.92818	-2.38833	-0.62754
H	-1.66016	2.31885	-0.63397	-2.70539	-2.05069	-0.67205	-1.97601	2.42709	0.81383	-2.47569	-2.34960	-0.81701
C	-0.58843	-0.02750	0.08740	-0.58196	-0.40509	-0.26520	-0.79565	0.03683	0.28388	-0.81280	-0.28332	-0.21488
C	-0.07984	-0.80641	1.13632	-0.07535	-1.03997	-1.40415	-0.02421	0.36515	1.40833	-0.05117	-0.81042	-1.26411
C	1.24730	-1.18198	1.14636	1.25319	-1.42854	-1.46023	1.33848	0.14137	1.41170	1.32570	-0.91028	-1.15133
C	2.09159	-0.73148	0.14121	2.08753	-1.12083	-0.40115	1.92369	-0.46435	0.31116	1.94310	-0.41480	-0.01690
C	1.55803	0.14758	-0.82082	1.55048	-0.39723	0.68244	1.08951	-0.88385	-0.73909	1.14163	0.20543	0.96225
N	0.25495	0.44352	-0.83153	0.25631	-0.08279	0.72624	-0.21654	-0.60310	-0.73312	-0.18541	0.23296	0.84710
C	3.52041	-1.17631	0.24285	3.51264	-1.58199	-0.38821	3.40142	-0.71518	0.37913	3.42023	-0.56515	0.18287
C	2.37251	0.82598	-1.88163	2.38790	0.05806	1.83702	1.58574	-1.67531	-1.91232	1.71890	0.87008	2.17324
N	-0.89465	-1.19858	2.28839	-0.88519	-1.27714	-2.60080	-0.61549	0.88794	2.64170	-0.64381	-1.22356	-2.53714
H	1.63470	-1.79611	1.94541	1.61737	-1.95297	-2.33206	1.93187	0.41099	2.27305	1.89524	-1.35883	-1.95273
O	-0.74088	-2.32756	2.73424	-1.68213	-0.41520	-2.93881	-1.67903	0.41601	3.01410	-1.55435	-0.54928	-2.99417
O	-1.65737	-0.36831	2.75937	-0.69073	-2.31594	-3.21836	0.00904	1.74728	3.24910	-0.16694	-2.20468	-3.09245
H	3.37785	1.05683	-1.54136	3.36434	0.40959	1.50996	2.43412	-2.30452	-1.65118	2.64932	1.38674	1.94316
H	1.86879	1.74157	-2.18175	2.55895	-0.77077	2.52613	1.90076	-1.00511	-2.71625	1.94198	0.12542	2.93936
H	2.45529	0.18872	-2.76583	1.87069	0.85223	2.36933	0.77690	-2.29332	-2.29527	0.99627	1.57496	2.57678
N	4.14553	-1.43618	-0.94756	4.21864	-1.25469	-1.51037	4.10133	-0.25763	-0.70073	4.16788	-0.16496	-0.88983
O	4.05736	-1.32926	1.32937	3.98300	-2.19712	0.55716	3.90909	-1.25082	1.35430	3.88627	-1.01079	1.22155
H	3.54768	-1.61793	-1.74011	3.69575	-0.94759	-2.31680	3.59565	0.25316	-1.40694	3.68558	0.26118	-1.66514
N	5.33213	-2.14798	-1.05203	5.41833	-1.86022	-1.87451	5.44016	-0.26530	-0.94524	5.51651	-0.16736	-1.07050
C	6.36514	-1.76285	-0.40635	6.41105	-1.80343	-1.07262	6.26590	-0.85234	-0.16042	6.31340	-0.66790	-0.20048
C	6.62485	-0.54926	0.38708	6.61352	-1.06133	0.18295	7.69957	-0.81328	-0.45832	7.75824	-0.63419	-0.43934
H	7.20898	-2.44152	-0.49939	7.27045	-2.37699	-1.41017	5.95938	-1.38066	0.73217	5.97366	-1.12061	0.72109
C	6.15642	0.71273	0.02332	6.07320	0.20185	0.42692	8.24324	-0.14591	-1.55992	8.34236	-0.05568	-1.57009
C	7.45110	-0.63743	1.50625	7.46275	-1.60162	1.14771	8.58432	-1.46368	0.40290	8.61148	-1.19956	0.50935
C	7.72874	0.51277	2.23151	7.69503	-0.89085	2.31712	9.94634	-1.42077	0.13497	9.98302	-1.16477	0.29429
N	7.26918	1.72196	1.89664	7.16979	0.31278	2.56108	10.46901	-0.78322	-0.91520	10.54441	-0.61209	-0.78363
C	6.50611	1.80570	0.80151	6.38354	0.84151	1.61689	9.61584	-0.16126	-1.74087	9.72144	-0.07118	-1.69291
H	6.15578	2.79487	0.53088	5.97658	1.82464	1.82220	10.05561	0.35147	-2.58840	10.19195	0.37313	-2.56233
H	8.35323	0.45936	3.11565	8.33659	-1.30536	3.08586	10.64496	-1.92030	0.79612	10.65722	-1.59979	1.02295
H	7.86282	-1.58839	1.81632	7.92952	-2.56542	0.99507	8.21725	-1.99380	1.27167	8.21276	-1.65953	1.40377
H	5.54484	0.85118	-0.85460	5.42905	0.68599	-0.29093	7.60648	0.37684	-2.25825	7.73128	0.39894	-2.33586
